# Estimating global numbers of fishes caught from the wild annually from 2000 to 2019

**DOI:** 10.1017/awf.2024.7

**Published:** 2024-02-08

**Authors:** Alison Mood, Phil Brooke

**Affiliations:** 1 Fishcount.org.uk; 2 Compassion in World Farming International, River Court, Mill Lane, Godalming, GU7 1EZ, UK

**Keywords:** Animal welfare, capture fisheries, estimated numbers, fish capture, fish slaughter, fish welfare

## Abstract

Finfishes are caught from the wild for food, feed (often in the form of fishmeal and oil) and bait. According to the Food and Agriculture Organisation of the United Nations (FAO), between 74 and 83 million tonnes (averaging 77 million tonnes) were caught annually in 2000–2019. Although fishes are now widely recognised as sentient beings, capture is still quantified as biomass rather than number of individuals (in contrast to wild-caught marine mammals and crocodiles; and farmed mammals and birds). Here, we estimate global numbers of wild-caught finfishes using FAO capture production (landing) tonnages (2000–2019 data) and estimates of mean individual weight at capture, based on internet-sourced capture and market weights. We estimate that between 1,100 and 2,200 billion (1.1–2.2 × 10^12^), or 1.1–2.2 trillion, wild finfishes were caught annually, on average, during 2000–2019. Anchoveta (*Engraulis ringens*) comprised 28%, by estimate midpoint. Estimated numbers in 2019, totalling 980–1,900 billion, were lower due to reduced anchoveta landings, but still represented 87.5% of vertebrate numbers killed for food or feed, as obtained or estimated from FAO data. These figures exclude unrecorded capture such as illegal fishing, discards and ghost fishing. Estimated finfish numbers used for reduction to fishmeal and oil represented 56% of the total 2010 estimate (1,000–1,900 billion), by midpoint. It is recommended that the FAO reports fish capture numbers. The welfare of wild-caught fishes, which is generally very poor during and after capture, should be addressed as part of sustainable utilisation of aquatic resources.

## Introduction

Wild capture fisheries impact the welfare of fishes during and after capture (van de Vis & Kestin 1996; Gregory 1998; Metcalfe 2009; Hürlimann *et al.* 2014; Veldhuizen *et al.* 2018; Anders *et al.* 2019, 2021; Breen *et al.* 2020). The Food and Agriculture Organisation of the United Nations (FAO) (2021a) reports that global finfish capture production (landings) totalled over 79 million tonnes in 2019, suggesting capture numbers are high. Numbers are important since the magnitude of an animal welfare problem may be measured as the product of the severity, duration and numbers of animals affected (World Society for the Protection of Animals [WSPA] 2003). This study therefore estimates numbers of fishes caught from the wild each year.

Capture fisheries are important for the food, nutrition, and employment of millions of people, according to the FAO (2018), and the sustainable management of fisheries is central to safeguarding food security, livelihoods and natural resources (FAO 2022). Animal welfare is considered an essential component of the sustainable use of animals (Broom 2010), and the FAO has included animal welfare as a key spoke in its guidelines on the Sustainability Assessment of Food and Agriculture Systems (SAFA) (FAO 2014).

Despite wide acceptance of fish sentience, and the importance of animal welfare for sustainability, FAO statistics for fishes are given only in tonnages and not numbers (FAO 2021a). This contrasts with FAO statistics for wild-caught crocodiles and marine mammals (FAO 2021a), which are given in numbers, and farmed birds and mammals, which are given in both (FAO 2023a). Previous studies have estimated farmed fish numbers from FAO production tonnages (Franks *et al.* 2021; Mood *et al.* 2023). The present study converts FAO finfish capture production tonnages to numbers using estimates of mean fish capture weights.

Research evidence confirms that fish species are capable of nociception (detection of painful stimuli) and appear to experience a negative affective state as well (Sneddon 2009). Acceptance of fish sentience is implicit in the farmed fish welfare codes of the World Organisation for Animal Health (OIE) (2023a), and in national legislation across the world, often covering farmed fishes in albeit general animal welfare provisions (Mood *et al.* 2023).

The OIE has published guidelines to protect the welfare of farmed fishes during slaughter (OIE 2023b) last adopted in 2012. Although specifically aimed at farmed fishes, the same principles apply for the humane killing of fishes caught from the wild. OIE guidelines state that fishes should be stunned before killing, to ensure an immediate loss of consciousness, and killed before consciousness is recovered if the stunning is not irreversible (OIE 2023b). However, since most wild-caught fishes are not stunned (van de Vis & Kestin 1996; Metcalfe 2009; Anders *et al.* 2019; Breen *et al.* 2020), current practices in fishing are likely to cause very poor welfare.

The numbers of animals involved are large. Using estimated mean weights (EMWs), derived from internet-sourced fish capture weights and combined with FAO capture production tonnages, Mood and Brooke (2010) estimated that (to two significant figures) between 970 and 2,700 billion (9.7 × 10^11^–2.7 × 10^12^) fishes were caught from the wild, on average, annually between 1999 and 2007 (not peer-reviewed). This was updated to 790–2,300 billion (7.9 × 10^11^–2.3 × 10^12^) annually for 2007–2016 (Mood & Brooke 2019a).

The present study aims to refine and update the earlier estimates for annual wild-caught fish numbers between 2000–2019. These include average annual estimates for:

- Individual species and countries with the highest capture numbers (for recorded landings);
- Fishes used for reduction to fishmeal and oil, based on Cashion *et al.* (2017) and, for comparison, Wijkström (2012);
- Fishes caught within certification schemes, based on Potts *et al.* (2016); and
- Fishes caught in countries where some animal welfare law covers fish slaughter in aquaculture.

Estimated finfish numbers are then compared with numbers of other vertebrates killed for food in 2019, obtained or estimated from FAO data (FAO 2021a,b, 2023a).

## Materials and methods

This study used EMWs for wild-caught finfish species to estimate numbers caught globally from FAO fisheries capture production tonnages, annually over the period from 2000 to 2019. All data were stored on a MySQL database, with calculations performed in MySQL code and Microsoft Excel®.

### FAO production tonnage data

A list of wild-caught finfish species categories, and their capture production tonnage for each country and major fishing area by year from 2000 to 2019, was obtained from the FAO (2021a) using ‘FishStatJ’ software. The FAO assigns capture to the country of the flag flown by the fishing vessel (FAO 2023b). From FishStatJ, data were selected for all countries, all fishing areas and all species in the species main group ‘PISCES’.

The FAO is the only source of global fish capture statistics, which represent a unique global asset for sector analysis and monitoring (FAO 2018). These statistics are primarily based on data submitted by member countries, which may be complemented or replaced with data from other sources, such as regional fisheries bodies with assessment responsibility for a stock (FAO 2018, 2022).

Not all species are reported separately, with some reported by genus, family, order or vague species groupings such as ‘Marine fishes nei’. ‘Nei’ is short for ‘not elsewhere included,’ a term used by FAO in the absence of specific species information. The FAO collaborates with countries to improve the level of species breakdown and quality of their statistics, e.g. supporting projects to improve standardisation of sampling at landing sites (FAO 2018).

However, the FAO capture production database does not include all fishes caught in the wild, as it omits the portion of the catch that is discarded at sea and catches from illegal fishing (FAO 2018), and other unrecorded fishing mortality (discussed later).

FAO data provide the genus, family and taxonomic order, as applicable, for each category of species. The FAO species categories were grouped into taxonomic classes, according to FishBase (Froese & Pauly 2021). The major fishing areas given in the FAO data were each assigned to one of the following groupings, according to name: Atlantic Ocean, Indian Ocean, Mediterranean and Black Sea, Pacific Ocean, Arctic Sea and inland waters.

To investigate changes in landings of anchoveta (*Engraulis ringens*), also called Peruvian anchovy, annual FAO capture production tonnages for this species were averaged over rolling 10-year periods from 1991–2000 to 2010–2019. Anchoveta has a large effect on total numbers, since this is the most numerous species caught (Mood & Brooke 2010).

### Collection of data for EMWs

Internet searches were performed to obtain capture or market sizes for fish species, starting with those with the highest capture production tonnages. In addition, fish size data from Mood and Brooke (2010), obtained in a similar way, were also included.

Searches were performed in Google and Google Scholar. Search terms included the scientific name (in quotes) and one of the following (in quotes): ‘mean weight’, ‘average weight’ or ‘whole round’, the latter aimed at market weights for completely whole fishes. For example, “‘*Engraulis encrasicolus*’ ‘average weight’”. Data were not always obtained when using these search terms and, therefore, variations on them were also used, e.g. ‘weight’, ‘size’ or ‘kg’ instead of ‘average weight’.

Only sizes relating to wild, and not farmed, fishes were collected. These were usually weights. They comprised capture sizes; market sizes for a whole fish (i.e. both ‘whole’ and ‘round’); and in a small proportion of cases, those given simply as the size for a wild species, which were assumed to relate to fishery capture (rather than the entire under-water population or survey fishing). Market weights were assumed to represent the weight of an entire fish, unless otherwise stated (this assumption is tested in the sensitivity analysis). Occasionally, headed and/or gutted weights were collected, where suitable data were obtained for conversion to the live weight. Fish lengths were occasionally obtained in the searches, and included for species for which no suitable weight data were found, where length-weight (LW) conversion data were available (see below).

Weights converted from fish lengths were sometimes used, in the absence of collected weight data, using length data from searches and common lengths obtained directly from FishBase (Froese & Pauly 2021). Fish lengths were converted to weights where corresponding LW formula were available for the species, on FishBase (Froese & Pauly 2021) or in the same reference.

LW formulae have the form: Weight = a × Length^b^ where a and b are constants (Froese *et al*. 2011). Sometimes length-length formulae, also obtained from FishBase (Froese & Pauly 2021), were used where the type of length being converted (e.g. total length or length to the fork of the tail) did not match that specified for the LW formula.

### Derivation of EMWs

Since mean individual fish capture weights are not included in FAO fishery statistics, EMWs were extrapolated from other data. The aim was to obtain the most precise, while still reliable, EMWs from the fish sizes (weights and lengths) collected in internet searches.

After converting the fish lengths to weights (see below), the collected data were each categorised as one of the following six types of fish capture weight, some advantages and disadvantages of which are as follows:

1. Mean or average weights. Mean weights are the most relevant data for estimating the global mean weight. Reported average weights were assumed to represent mean weights.
2. Mean weights from survey fishing. These could be smaller than the mean capture weight in a fishery, depending on the selectivity of the survey fishing gear, especially if they include immature fishes. ‘Mature’ or ‘adult’ fishes were assumed to be within the fishery capture size range.
3. Simple weight ranges. These are likely to span the mean weight but may be imprecise i.e. give a wide range.
4. Usual weights. These were assumed to be the mode, which may differ from the mean weight, depending on size distributions.
5. Common weights. These may also differ from the mean and mode; common may not mean the most common size.
6. Weights converted from lengths. Length-weight conversion is more reliable where the length being converted is within the length range for which the LW formula was derived (Froese 1998). Weights converted from a mean length are likely to under-estimate the mean weight due to the non-linear relationship between weight and length, inherent in the LW formula (Beyer 1991). For usual and common lengths, and simple length ranges, issues identified above for the corresponding type of weight will similarly apply.

Fish individual weights vary between time, place and conditions of capture (see *Discussion*). Including more than one fish weight, preferably from different fisheries or markets, can increase representativeness, though potentially also widening the range.

EMWs were obtained for one species at a time. The fish weight data to be included in each EMW were selected via a system for ranking data, according to the type of fish weight, as listed above. The data ranking system for the main estimate (Table 1) aimed to achieve a compromise between selecting the most relevant, or reliable, and precise data; and including more references to increase representativeness. It worked as follows:

- Mean, average and usual fishery capture or market weights were used where available; also mean weights from survey fishing that relate to mature fishes;
- If these were not available, simple weight ranges and common weights, and survey fishing mean weights not restricted to mature fishes, were used; and
- If capture/market weights were not available, weights converted from fish lengths were used.

**Table 1.** Ranking of fish weight data in the main and alternative estimates

Fish weight data type	Data ranking in each estimate					
	Main (& A6, A7)	A1	A2	A3	A4	A5
Mean or average weight. Includes those from survey fishing if for mature fishes	1	1	1	1	1	1
Usual or modal weight	1	2	1	1	1	1
Marketing website mean or usual weight	1	4	1	1	2	1
Simple weight range	2	1	1	2	2	1
Common or typical weight	2	3	2	2	2	1
Survey fishing mean weight (if not solely mature fishes)	2	4	2	1	2	1
Weight converted from length (mean/average, usual/modal, common or typical)	3	5	3	3	3	1

Footnote. Table shows the ranking of fish weight data in the main estimate, and in each of the alternative estimates A1 to A7. The data ranking gives the rules for determining which fish weights, from those obtained using internet searches, are used to derive each estimated mean weight (EMW). ‘1’ indicates the highest ranked data. Higher ranked data are used in preference to lower ranked data, where available. In the main estimate, mean, average and usual weights, including mean weights relating to adult fishes caught in survey fishing (rank 1), are used in preference to simple weight ranges, common weights and other mean weights from survey fishing (rank 2), which are likewise preferred to weights converted from fish lengths (rank 3). Estimates A1 to A5 differ only in the data ranking, as shown. For example, A5 includes all types of fish weight (rank 1). Estimates A6 and A7 use the same rankings as the main estimate.

In addition to this main estimate, some alternative estimates were made (A1–A7) using different data ranking systems (Table 1), which were compared in the sensitivity analysis (discussed later).

Each EMW was obtained as the outside range of the selected fish weights. Note that most EMWs, even those based on mean weights, were usually derived as a range since fish weights (and lengths) are often reported as such and EMWs were often based on more than one fish weight.

### Estimates of numbers for species

For species categories for which an EMW was obtained, fish number ranges were estimated by dividing the respective global tonnage by the EMW range. These species categories almost always comprised a single species; occasionally weight data were found for a genus or family.

For species categories for which no EMW was obtained, generic estimated mean weights (GEMWs) were used to estimate fish number ranges. Such categories included those for species for which searches returned no suitable fish size data, and almost all multi-species categories.

GEMWs were extrapolated from EMW estimates. The GEMW for each group, whether a genus, family, order, class, or all species combined, was calculated as follows. The GEMW is the mean weight for all species with an EMW in that group, obtained from their total estimated numbers and total tonnage.

Numbers were estimated for a species category by obtaining a matching GEMW; matching on the genus where possible, otherwise on the family and so on, thereby using data for the most closely related species from that available.

Fish numbers were estimated for each species category, as described, globally for each year between 2000 and 2019. Tonnage and estimated numbers were then averaged for all years. While EMWs are the same for all years, GEMWs can vary between years, due to changes in the proportions of species on which they are based. An average global GEMW for 2000–2019 was also obtained, for each species category without an EMW, back-calculated from its total tonnage and estimated numbers for all years combined (see following section).

### Estimate totals

Estimates based on EMWs and GEMWs were summed to give the total global estimate, for each year and on average annually for 2000–2019. The difference between the lowest and highest annual estimate was analysed by species.

Average annual numbers were also obtained separately for each country, continent and fishing area. These subtotals were calculated using EMWs and average global GEMWs for 2000–2019 (see previous section).

### Sensitivity analysis

A total of seven alternative estimates, A1 to A7 (discussed below), were performed for each year between 2000–2019, averaging the results of each for the period.

Alternative estimates A1 to A5 were performed to test the sensitivity of the estimate to changes in the data ranking system. A1–A5 each used a different data ranking system (Table 1), and only data of the highest ranking available were included for each species, with 1 being the highest ranking. A1–A5 differed from the main estimate only in the data ranking system used.

Alternative estimate A6 concerned fish weights obtained from seafood marketing and food-related websites. It was assumed, in the main estimate, that these represented an entire whole fish unless stated otherwise. A6 tested the alternative assumption that such weights were in fact headed and gutted, converting them to live weights. A typical conversion factor of 2.0 was chosen for headed and gutted weights, based on UK Government (2018). Weights stated as ‘whole’, but not also as ‘round’, were assumed to be gutted weights. For these, a typical conversion factor of 1.15 was chosen, likewise based on UK Government (2018).

Alternative estimate A7 tested a different estimating method for some tonnages without an EMW, similar to Mood and Brooke (2010). For species categories comprising a single genus or family, multi-species EMWs were calculated from other EMWs, as obtained for the main estimate. as follows. Multi-species EMWs were obtained by combining EMW ranges for the smallest and largest species in the group with an EMW (more precisely the species with the smallest lower EMW and the species with the largest upper EMW), where available. Occasionally, in the absence of related EMWs from the main estimate, multi-species EMWs were alternatively based on one or two additional EMWs, obtained for species in the group for which fish weight data were available.

### Estimates for reduction fisheries

Numbers of fishes used for reduction to fishmeal and oil in 2010 were estimated using data from Cashion *et al.* (2017). Cashion *et al.* (2017) give the species composition for 53% of total fisheries capture destined for reduction in 2010, which they estimated to total 16.6 million tonnes (Cashion *et al.* 2017 [in Table S2 of the Supplementary material]). Their analysis was based on ‘reconstructed catch’ data, as discussed later for Pauly and Zeller (2016) but excluding discarded bycatch. Fish numbers for this 53% were estimated using EMWs and one average global GEMW for 2000–2019.

The remaining 47% of tonnage used for reduction was assumed to comprise other finfishes and some Antarctic krill (*Euphausia superba*), a crustacean species that is similarly used (Katevas 2014). After deducting an estimated tonnage of Antarctic krill used for this purpose, estimates for this tonnage without species information were extrapolated, using a new GEMW back-calculated from the total tonnage and estimated numbers of fishes in the 53% of tonnage used for reduction for which the species was given.

For comparison, a separate similar estimate of fish numbers caught for reduction in 2001–2006 was made, using data on proportions of capture production so used, by species and country, from Wijkström (2012).

Froehlich *et al.* (Froehlich 2018), suggested that capture of the smaller marine fish species used for reduction (‘forage fish’) could be reduced by six million tonnes per year, as part of precautionary management measures. These authors list the 20 species classified as ‘forage fish’ in their analysis, by tonnage in 2012 (Froehlich *et al.* 2018 [in Table S1 of the Supplementary material]). To estimate the numbers of fishes affected by this proposed decrease in capture, the overall estimated mean weight for these 20 species was calculated from their combined estimates for 2000–2019.

### Estimates for certified fisheries

Numbers of fishes caught in capture certified by the Friend of the Sea (FOS) and Marine Stewardship Council (MSC) schemes, in 2014 and 2015, respectively, were estimated using certified tonnages obtained from Potts *et al*. (2016). Potts *et al.* (2016) list the finfish species constituting this certified capture, by common name, with their certified tonnages. Numbers were estimated for each of these tonnages for which there was a corresponding FAO species category, using the estimated mean weight (EMW or GEMW) derived for the species category, for 2000–2019. In most cases, the common name given by Potts *et al*. (2016) matched an FAO species category, and the appropriate EMW was used. Where there was no exact match, the website of the FOS or MSC was used to determine the most likely species involved (FOS 2023; MSC 2023).

In some cases, the certified tonnages exceeded the tonnage reported by the FAO (2021a) in the corresponding FAO species category, and so adjustment was made as follows. Actual certified tonnages for species are expected to be less than the corresponding FAO tonnage, if the latter is correct and represents the whole catch, with none reported in other species categories. To adjust for potentially anomalous figures, estimates were based on the lower of the certified tonnage according to Potts *et al*. (2016) and the corresponding FAO tonnage. Reported certified tonnages for FOS and MSC were compared with the FAO tonnage for 2014 and 2015, respectively. In the special case of South American pilchard (*Sardinops sagax*), certified tonnage was compared with the combined FAO tonnage for all species in the genus *Sardinops.* Though the FAO reports *Sardinops* species separately, FishBase (Froese & Pauly 2023) considers these to be synonyms of *Sardinops sagax.*

### Analysis of fish protection law

Affording fishes some legal protection of their welfare during and after wild-capture is likely to begin in countries that have analogous laws for aquaculture. Animal welfare law, relating to farmed fishes during slaughter, was analysed for the 30 countries with the highest finfish capture numbers (by midpoint) for 2000–2019. The aim was to identify countries with any welfare law covering farmed fish slaughter, according to the wording of law and current authors’ interpretation, excepting those laws solely aimed at preventing malicious cruelty. Estimated fish numbers were then combined for countries that were determined as having such legislation.

Firstly, reference was made to the Animal Protection Index report for each country included, published by World Animal Protection (2020), which analyses the country’s main animal protection laws. Internet searches were then performed, in Google and Google Scholar, to locate relevant laws. These search terms were used: ‘animal welfare law’ or ‘animal law’ followed by the name of the country; or name of the law. Laws were translated into English, as necessary, using Google Translate. The legal texts obtained were then studied for information on the species covered and any protection applicable to farmed fishes during slaughter. Where these texts were not clear, other sources of information obtained in the same internet searches or obtained from the FAOLEX Database website (FAO 2023c), were additionally used when available.

Having obtained a list of top countries that were determined as having such legislation, this was combined with the list of all other EU countries, since EU law (Regulation [EC] No 1099/2009) prohibits causing fishes avoidable pain or suffering during slaughter (European Union 2009). Since this law has also transferred into UK law, the UK was additionally included.

### Estimates for other vertebrates

To enable the comparison of estimated finfish numbers with numbers of other vertebrates, tonnages for turtles and frogs reported in FAO production statistics for wild capture (FAO 2021a) and aquaculture (FAO 2021b) were converted to numbers using estimated mean weights, since these species groups are not reported in numbers. Tonnages for 2019 were obtained using FishStatJ software (FAO 2021a,b). For wild-caught turtles, which mostly comprised unnamed marine turtles (Testudinata), an estimated mean weight of 0.5–19.8 kg was used, based on Nijman (2010) and Pham *et al.* (2013). For farmed turtles, which mostly comprised Chinese softshell turtle (*Trionyx sinensis*), an estimated mean weight of 1.0–1.5 kg was used, based on FAO (2023d). For farmed and wild frogs, which mostly comprised unnamed frogs (*Rana*), an estimated mean weight range of 50–250 g was used, based on Cagiltay *et al*. (2014) and Zhu *et al*. (2021).

Another group of vertebrates that FAO reports in tonnages only, is that of hunted terrestrial animals represented by 1.98 million tonnes of ‘game meat’ produced in 2019 (FAO 2023a). Since no information on taxa is given (FAO 2023a), no attempt was made to estimate numbers comprising this tonnage.

## Results

All fish (and frog and turtle) number estimates in the presented results, including midpoints, are rounded to two significant figures.

### FAO data

The FAO reported capture production for 1,725 categories of finfish species in the period 2000–2019, for which annual totals ranged from 74.0 million tonnes in 2010 to 83.3 million tonnes in 2018 and averaged 77.3 million tonnes (FAO 2021a). Finfish capture production for 2019 comprised 1,569 of these species categories, totalling 79.4 million tonnes.

Anchoveta capture production averaged 6.7 million tonnes annually for 2000–2019 (FAO 2021a). This species showed the greatest variation in absolute capture production tonnage, ranging between 3.1 million in 2014 and 11.3 million in 2000 (Supplementary Table S1). Average annual capture production for the decade to 2019 equated to 62% of that for the decade to 2011 (Supplementary Table S1).

### Collection of data for EMWs

A total of 805 fish individual weights were obtained from fish sizes (weights and lengths) collected from internet searches. After 114 weights were excluded by the data ranking system, the main estimates for 2000–2019 used 691, which included 200 from Mood and Brooke (2010). These 691 fish weights were categorised, according to the data types shown in Table 1, as follows: 483 mean or average weights, 76 simple weight ranges, 51 survey fishing mean weights; 38 usual weights; nine common or typical weights and 34 weights converted from fish lengths. All related to a single-species except for seven relating to a genus and one relating to a family. The sources of collected fish sizes on which these 691 weights were based (657 fish weights, and 34 fish lengths) are summarised in Supplementary Table S2. Most of these fish sizes were obtained from research articles, seafood marketing websites, or government or inter-government publications (Supplementary Table S2).

### Derivation of EMWs

A total of 480 EMWs were obtained for the main estimates for 2000–2019, representing 472 single-species and eight multi-species categories. EMW ranges for these are shown in Table S3 in the Supplementary material, indicated by an EMW type beginning ‘S’ or ‘M’ for single and multi-species categories, respectively. Ten of these single-species categories had no reported tonnage for 2019, and the 2019 main estimate was therefore based on 470 EMWs, in turn based on 679 fish weights.

An EMW was obtained for 97% of all single-species categories, by tonnage, in the estimate for 2019 and the average annual estimate for 2000–2019.

The 480 EMWs for 2000–2019 represented 293 genera, 129 families, 41 orders and five classes; enabling GEMWs to be calculated for each of these taxa.

### Estimates of numbers for species

Global estimated numbers for all species categories with an EMW, on average annually for 2000–2019, are shown in Supplementary Table S3, indicated by an EMW/GEMW type beginning ‘S’ for single-species or ‘M’ for multi-species EMWs. These comprised 62% of the average annual estimate, by tonnage, and totalled 720–1,500 billion (7.2 × 10^11^–1.5 × 10^12^) fishes (Table 2).

**Table 2.** Estimated average annual wild-caught finfish number ranges (2000–2019), by estimating method

			Estimated numbers in billions (× 10^9^)				
Fish weight data ranking and type		Capture (‘000 tonnes)	Lower	Upper	Midpoint	% of total capture tonnage	% of total estimate midpoint
Single-species EMWs							
1	Mean, average or usual weight	40,869	600	1,200	930	53	57
2	Common weight, simple weight range, mean weight from survey fishing (if not solely mature fishes)	5,229	28	51	40	7	2
3	Weight converted from length	831	7.9	28	18	1	1
Multi-species EMWs							
1	Mean, average or usual weight	805	40	40	40	1	2
2	Common weight, simple weight range	442	44	120	83	1	5
Total for EMWs		**48,177**	**720**	**1,500**	**1,100**	**62**	**68**
GEMWs							
	GEMW for genus	6,685	41	75	58	9	4
	GEMW for family	4,876	53	110	83	6	5
	GEMW for order	2,404	21	41	31	3	2
	GEMW for class	525	3.6	7.4	5.5	1	0.3
	GEMW for all fishes (used where there is minimal species information)	14,682	220	450	330	19	21
Total for GEMWs		**29,172**	**340**	**690**	**510**	**38**	**32**
Total		**77,348**	**1,100**	**2,200**	**1,600**	**100**	**100**

Footnote: Table shows a breakdown of global wild-caught finfish numbers (averaged annually 2000–2019), estimated from capture production tonnages (landings) reported by the FAO (2021a) and estimated mean individual weights (EMWs) for species, by type of data used to estimate mean weights. For tonnages where no EMW was obtained for the category of species, generic estimated mean weights (GEMWs) extrapolated from EMW estimates were used. Estimates based on EMWs and on GEMWs for the genus, being based on data for the same and closely related species, respectively, are likely to be more reliable and together comprise 72% of the total estimate midpoint. For more information on data types included for each data ranking, see Table 1 (main estimate). Estimated numbers are rounded to 2 significant figures.

In total, there were 1,245 species categories for which no EMW was obtained (1,099 of such categories for 2019). Global estimated numbers for these, on average annually for 2000–2019, are also shown in Supplementary Table S3. These are based on GEMWs and indicated by an EMW/GEMW type beginning ‘G’. For, respectively, 399, 494, 242 and 50 of these categories, fish numbers were calculated from GEMWs corresponding to a single genus, family, order or class; for eight categories using the GEMW for all species combined; and for 52 categories using a combination of GEMW types depending on the year. A total of 263 distinct GEMWs were used. Estimates based on GEMWs comprised 38% of the average annual estimate, by tonnage, and totalled 340–690 billion (3.4–6.9 × 10^11^) fishes (Table 2).

For the GEMW estimates, for 18% of total tonnage, numbers were based on GEMWs for related species, employing data for the same genus, family or taxonomic order (Table 2). For 20% of total tonnage, numbers were based on the GEMW for all species combined, or a GEMW for the class (Table 2). Virtually all this tonnage had minimal species information, without the taxonomic order, including the categories ‘Marine fishes nei’ and ‘Freshwater fishes nei’ which together comprised 19% of total tonnage.

Global estimated numbers for the top 40 species categories, for 2019 and averaged annually for 2000–2019, are shown in Tables 3 and 4, respectively.

**Table 3.** Estimated global wild-caught finfish number ranges (2019), ranked by estimate midpoint

			Estimated mean weight (EMW/GEMW) (g).		Estimated numbers in billions (× 10^9^)		
Rank	FAO species category	Capture (‘000 tonnes)	Lower	Upper	Lower	Upper	Midp’t
1	Anchoveta (=Peruvian anchovy) (*Engraulis ringens*)	4,249	10	29	150	420	290
2	Marine fishes nei (Osteichthyes)	10,301	*39*	*76*	*140*	*270*	*200*
3	Freshwater fishes nei (Osteichthyes)	6,161	*39*	*76*	*81*	*160*	*120*
4	Stolephorus anchovies nei (*Stolephorus* spp.)	390	3	7	56	160	110
5	European pilchard (=Sardine) (*Sardina pilchardus)*	1,499	20	20	75	75	75
6	European sprat (*Sprattus sprattus*)	541	9	9	64	64	64
7	Japanese anchovy (*Engraulis japonicus*)	930	20	22	42	47	44
8	European anchovy (*Engraulis encrasicolus*)	596	8	38	16	73	44
9	Cyprinids nei (Cyprinidae)	966	*20*	*31*	*31*	*49*	*40*
10	Silver cyprinid (*Rastrineobola argentea*)	336	9	15	22	37	30
11	Clupeoids nei (Clupeoidei**)**	565	*16*	*32*	*18*	*35*	*27*
12	Scads nei (*Decapterus* spp.)	1,297	*42*	*70*	*19*	*31*	*25*
13	Sandeels (=Sandlances) nei (*Ammodytes* spp.)	235	10	10	24	24	24
14	Araucanian herring (*Strangomera bentincki*)	320	11	25	13	28	21
15	Anchovies, etc. nei (Engraulidae)	263	*9*	*23*	*11*	*29*	*20*
16	Sardinellas nei (*Sardinella* spp.)	818	*51*	*75*	*11*	*16*	*13*
17	Indian oil sardine (*Sardinella longiceps*)	576	42	49	12	14	13
18	Blue whiting (=Poutassou) (*Micromesistius poutassou*)	1,517	80	300	5.1	19	12
19	Pacific sandlance (*Ammodytes personatus*)	114	10	10	11	11	11
20	Alaska pollock (=Walleye poll.) (*Gadus chalcogrammus*)	3,496	227	1,000	3.5	15	9.4
21	Atlantic herring (*Clupea harengus*)	1,559	100	600	2.6	16	9.1
22	Southern African anchovy (*Engraulis capensis*)	165	19	19	8.9	8.9	8.9
23	Pacific anchoveta (*Cetengraulis mysticetus*)	243	25	35	6.9	9.8	8.3
24	Cape horse mackerel (*Trachurus capensis*)	323	22	291	1.1	15	7.9
25	Black & Caspian Sea sprat (*Clupeonella cultriventris*)	55	6	10	5.7	9.0	7.4
26	Croakers, drums nei (Sciaenidae)	645	*68*	*128*	*5.0*	*9.4*	*7.2*
27	Silversides (=Sand smelts) nei (Atherinidae)	14	*1*	*7*	2.2	*12.0*	*6.9*
28	Bombay-duck (*Harpadon nehereus*)	193	26	32	6.0	7.5	6.8
29	Yellow croaker (*Larimichthys polyactis*)	310	36	66	4.7	8.7	6.7
30	Lizardfishes nei (Synodontidae*)*	181	*28*	*35*	*5.1*	*6.4*	*5.8*
31	Pacific chub mackerel (*Scomber japonicus*)	1,347	169	466	2.9	8.0	5.4
32	Largehead hairtail (*Trichiurus lepturus*)	1,133	196	265	4.3	5.8	5.0
33	Norway pout (*Trisopterus esmarkii*)	101	16	28	3.6	6.2	4.9
34	Californian anchovy (*Engraulis mordax*)	61	14	14	4.5	4.5	4.5
35	Pacific herring (*Clupea pallasii*)	480	64	426	1.1	7.5	4.3
36	Goldstripe sardinella (*Sardinella gibbosa*)	122	21	46	2.7	5.8	4.3
37	Japanese pilchard (*Sardinops melanostictus*)	767	180	180	4.3	4.3	4.3
38	Jack and horse mackerels nei (*Trachurus* spp.)	411	*57*	*374*	*1.1*	*7.2*	*4.2*
39	Indian mackerel (*Rastrelliger kanagurta*)	470	138	138	3.4	3.4	3.4
40	Yellowstripe scad (*Selaroides leptolepis*)	113	27	46	2.4	4.2	3.3
	Total for above (40 species categories)	**43,865**	*25*	*50*	**880**	**1,700**	**1,300**
	Other finfishes (1,529 species categories)	35,533	*191*	*336*	110	190	150
	Total estimate for 2019 (1,569 species categories)	**79,398**	*41*	*81*	**980**	**1,900**	**1,400**

Footnote: Table shows estimated number ranges for global wild-caught finfishes in 2019, for the top 40 species categories, ranked by descending estimate midpoint. Estimates are based on finfish capture production tonnages (landings) reported by the FAO (2021a) and estimated mean individual weights (EMWs) for species, expressed as a range from lower to upper. Where EMWs based on data for the species were not obtained, generic mean weights (GEMWs) extrapolated from EMWs were used. GEMWs are shown in italics. Estimates total 980–1,900 billion (9.8 × 10^11 -^ 1.9 × 10^12^) for 2019. Estimated numbers are rounded to 2 significant figures. Estimates for all species categories, averaged for the period 2000–2019, are shown in Supplementary Table S3.

**Table 4.** Estimated average annual global wild-caught finfish number ranges (2000–2019), ranked by estimate midpoint

			Estimated mean weight (EMW/GEMW) (g).		Estimated numbers in billions (× 10^9^)		
Rank	FAO species category	Average annual capture (‘000 tonnes)	Lower	Upper	Lower	Upper	Midp’t
1	Anchoveta (=Peruvian anchovy) (*Engraulis ringens*)^1,2^	6,651	10	29	230	670	450
2	Marine fishes nei (Osteichthyes)	9,229	*32*	*67*	*140*	*280*	*210*
3	Freshwater fishes nei (Osteichthyes)^5^	5,409	*33*	*68*	*80*	*160*	*120*
4	Stolephorus anchovies nei (*Stolephorus* spp.)	304	3	7	43	120	83
5	European sprat (*Sprattus sprattus*)^2^	587	9	9	69	69	69
6	Japanese anchovy (*Engraulis japonicus*)^1,2^	1,354	20	22	62	68	65
7	European pilchard (=Sardine) (*Sardina pilchardus)* ^2^	1,165	20	20	59	59	59
8	European anchovy (*Engraulis encrasicolus*)^2^	542	8	38	14	66	40
9	Sandeels (=Sandlances) nei (*Ammodytes* spp.)^1,2^	386	10	10	39	39	39
10	Araucanian herring (*Strangomera bentincki*)^3^	485	11	25	20	43	31
11	Capelin (*Mallotus villosus*)^1,2^	708	17	50	14	42	28
12	Scads nei (*Decapterus* spp.)	1,174	*42*	*70*	*17*	*28*	*22*
13	Clupeoids nei (Clupeoidei)	425	*15*	*32*	*13*	*28*	*21*
14	Cyprinids nei (Cyprinidae)^5^	556	*22*	*35*	*16*	*25*	*21*
15	Anchovies, etc. nei (Engraulidae)^2^	255	*10*	*25*	*10*	*26*	*18*
16	Pacific sandlance (*Ammodytes personatus*)^3^	172	10	10	17	17	17
17	Silver cyprinid (*Rastrineobola argentea*)^4,5^	185	9	15	12	21	16
18	Sardinellas nei (*Sardinella* spp.)^2^	913	*47*	*75*	*12*	*19*	*16*
19	Southern African anchovy (*Engraulis capensis*)	224	19	19	12	12	12
20	Atlantic herring (*Clupea harengus*)^1,2^	1,987	100	600	3.3	20	12
21	Blue whiting (=Poutassou) (*Micromesistius poutassou*)^1,2^	1,381	80	300	4.6	17	11
22	Black & Caspian Sea sprat (*Clupeonella cultriventris*)^5^	80	6	10	8.3	13	11
23	Indian oil sardine (*Sardinella longiceps*)^3^	478	42	49	9.7	11	10
24	Silversides (=Sand smelts) nei (Atherinidae)^5^	22	*1*	*7*	*3.3*	*18*	*10*
25	Croakers, drums nei (Sciaenidae)	723	*65*	*122*	*5.9*	*11*	*8.5*
26	Alaska pollock (=Walleye poll.) (*Gadus chalcogrammus*)	3,053	227	1,000	3.1	13	8.3
27	Cape horse mackerel (*Trachurus capensis*)^2^	336	22	291	1.2	15	8.2
28	Yellow croaker (*Larimichthys polyactis*)	340	36	66	5.2	9.5	7.4
29	Bombay-duck (*Harpadon nehereus*)	206	26	32	6.4	8.0	7.2
30	Goldstripe sardinella (*Sardinella gibbosa*)	183	21	46	4.0	8.7	6.4
31	Pacific chub mackerel (*Scomber japonicus*)^2^	1,460	169	466	3.1	8.6	5.9
32	Largehead hairtail (*Trichiurus lepturus*)	1,282	196	265	4.8	6.5	5.7
33	Pacific anchoveta (*Cetengraulis mysticetus*)^3^	148	25	35	4.2	6.0	5.1
34	Yellowstripe scad (*Selaroides leptolepis*)	170	27	46	3.7	6.3	5.0
35	Pacific saury (*Cololabis saira*)^3^	414	56	172	2.4	7.4	4.9
36	Gulf menhaden (*Brevoortia patronus*)^1,2^	479	95	127	3.8	5.1	4.4
37	Jack and horse mackerels nei (*Trachurus* spp.)	459	*66*	*394*	*1.2*	*7.0*	*4.1*
38	Lizardfishes nei (Synodontidae)	123	*29*	*36*	*3.4*	*4.3*	*3.9*
39	Pacific herring (*Clupea pallasii*)^2^	396	64	426	0.93	6.2	3.6
40	Chilean jack mackerel (*Trachurus murphyi*)^1,2^	1,157	200	1,000	1.2	5.8	3.5
	Total above (40 species categories)	**45,600**	*23*	*47*	**960**	**2,000**	**1,500**
	Other finfishes (1,685 species categories)	31,748	*186*	*326*	97	170	130
	Total estimate (1,725 species categories)	**77,348**	*36*	*73*	**1,100**	**2,200**	**1,600**

Footnote: Table shows estimated number ranges for global wild-caught fishes, on average each year in 2000–2019, for the top 40 species categories, ranked by descending estimate midpoint. Numbers are calculated from capture production tonnage (landings) reported by the FAO (2021a) and estimated mean individual weights (EMWs) for species. Where EMWs based on data for the species were not obtained, generic mean weights (GEMWs) extrapolated from EMWs were used. GEMWs are shown in italics. Total annual estimates average 1,100–2,200 billion (1.1–2.2 × 10^12^) for 2000–2019. Estimated numbers are rounded to 2 significant figures. Estimates for all species categories, averaged annually for 2000–2019, are shown in Supplementary Table S3.

Many fishes are caught for reduction to fishmeal and oil. 1 indicates the main species so used in 2010 based on Cashion *et al*. (2017). 2 indicates species generally/sometimes so used based on Wijkström (2012). 3 and 4 indicate some additional species so used, based on FishBase (Froese & Pauly 2023) and Kubiriza *et al*. (2021), respectively. 5 indicates all or some capture (> 1%) is from inland waters (FAO 2021a).

### Estimate totals

An estimated 980–1,900 billion (9.8 × 10^11^–1.9 × 10^12^) fishes were caught from the wild in recorded global capture in 2019 (Table 3) and, on average, 1,100 to 2,200 billion (1.1–2.2 × 10^12^) fishes were caught annually in 2000–2019 (Table 4). The lower estimate for 2019 is due to reduced landings of anchoveta, which is the top species by estimated numbers (Tables 3 and 4). Anchoveta comprised 28% of the 2000–2019 average estimate, by midpoint. These estimated number ranges give an overall estimated mean weight for all fishes landed of 41–81 g for 2019 (Table 3) and 36–73 g for 2000–2019 (Table 4).

**Figure 1.**
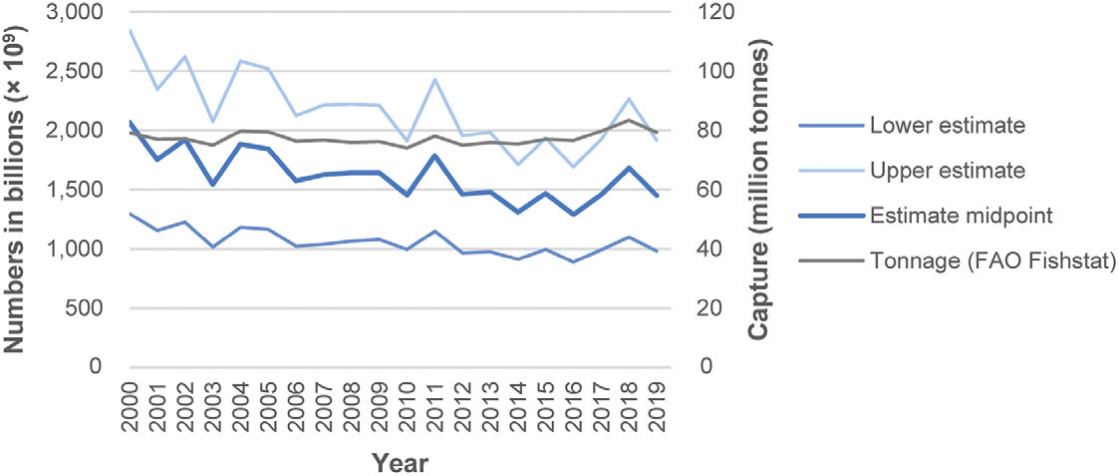
Estimated annual global wild-caught finfish number ranges for 2000–2019. Annual numbers, from lower to upper estimate (to 2 significant figures), average 1,100–2,200 billion (1.1–2.2 × 10^12^) individuals, with a midpoint of 1,600 billion (1.6 × 10^12^) individuals. Estimates are based on capture production tonnages (landings) reported by the FAO (2021a) and estimated mean individual weights for species.

**Figure 2.**
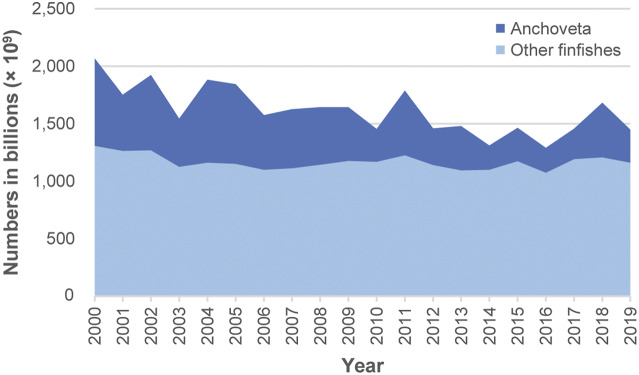
Estimated annual global wild-caught finfish numbers (i.e. midpoints of estimated number ranges) for anchoveta (*Engraulis ringens*) and all other species combined (2000–2019). Estimates are based on capture production tonnages (landings) reported by the FAO (2021a) and estimated mean individual weights for species. Inter-year differences in estimated numbers are mainly due to variable anchoveta capture tonnages. Lowest and highest annual capture numbers (estimate midpoints), to 2 significant figures, were as follows. Anchoveta numbers ranged between 210 billion (2.1 × 10^11^) in 2014 and 760 billion (7.6 × 10^11^) in 2000. Numbers for all other finfish species combined ranged between 1,100 billion (1.1 × 10^12^) in 2016 and 1,300 billion (1.3 × 10^12^) in 2000.

The midpoint for annual total estimates for 2000–2019 ranged between 1,300 billion (1.3 × 10^12^) in 2016, to 2,100 billion in 2000 (Figure 1, Table S4 in the Supplementary material). Most of this difference comprised anchoveta numbers; the rest predominantly comprising numbers for ‘Marine fishes nei’, ‘Sandeels (=Sandlances) nei’ (*Ammodytes* spp.) and capelin (*Mallotus villosus*) (Supplementary Table S4). Estimate midpoints for anchoveta showed larger inter-year differences compared with all other species combined (Figure 2).

**Figure 3.**
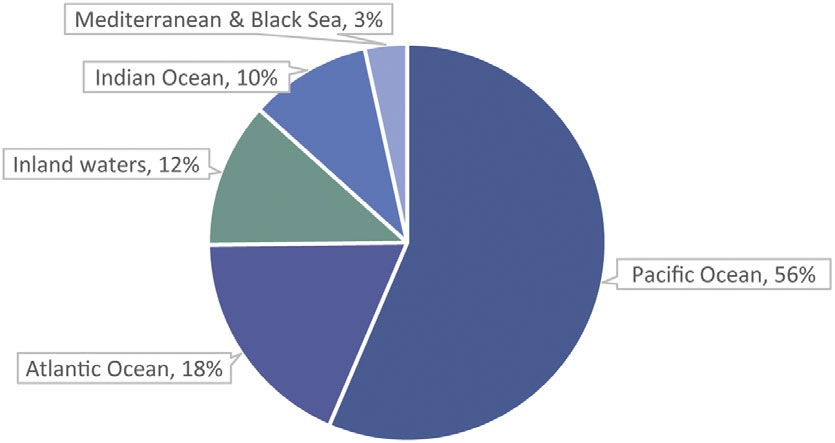
Percentages of estimated average annual wild-caught finfish numbers (i.e. midpoints of estimated number ranges) by fishing area. Most capture is from the marine environment, with the Pacific and Atlantic Oceans accounting for 75% of global numbers. Numbers are estimated from capture production tonnages (landings) reported by the FAO (2021a). Marine capture has more complete data (i.e. tonnages) available by species than inland capture (FAO 2020). Arctic Sea capture (less than 0.001% of capture tonnage) is not shown.

**Figure 4.**
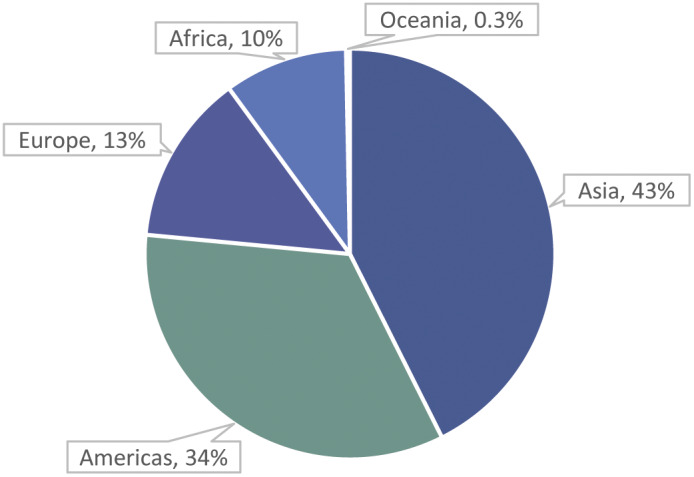
Percentages of estimated average annual wild-caught finfish numbers (i.e. midpoints of estimated number ranges) by continent. Asia and the Americans account for 76% of global numbers. Numbers are estimated from capture production tonnages (landings) reported by the FAO (2021a). Capture not assigned to any country (0.1% of capture tonnage) is not shown. Dominant finfish species groups are as follows. Asia: ‘Marine fishes nei’ (Osteichthyes), ‘Freshwater fishes nei’ (Osteichthyes) and ‘Stolephorus anchovies nei’ (*Stolephorus* spp.). Americas: ‘Anchoveta (=Peruvian anchovy)’ (*Engraulis ringens*). Europe: ‘European sprat’ (*Sprattus sprattus*), ‘Sandeels (=Sandlances) nei’ (*Ammodytes* spp.) and ‘Capelin’ (*Mallotus villosus*). Africa: ‘European pilchard (=Sardine)’ (*Sardina pilchardus*), ‘Freshwater fishes nei’ and ‘Silver cyprinid’ (*Rastrineobola argentea*). Oceania: ‘Clupeoids nei’ (Clupeoidei) and ‘Marine fishes nei’.

The majority of estimated fish numbers, 88%, are caught from the marine environment, with 12% caught from inland waters (Figure 3). For all but five of the top 40 species by estimated numbers for 2000–2019, capture production is all, or virtually all, marine capture (Table 4). The Pacific and Atlantic Oceans together account for 75% of fish numbers (Figure 3). By continent, Asia and the Americas together account for 76% of fish numbers (Figure 4).

Average annual estimates for the top 30 countries, by estimate midpoint, and for the EU27 countries combined, are shown in Table 5 for 2000–2019. Peru, China, EU27 and Chile together account for almost half of the global estimate. Estimated numbers for all countries are shown in Table S5 in the Supplementary material.

**Table 5.** Estimated average annual wild-caught finfish numbers (2000–2019) for the top 30 countries, by estimate midpoint

			Estimated numbers in billions (× 10^9^)					Overall estimated mean weight for country (g)			
Rank	Country	Average annual capture 2000-2019 (‘000 t)	Lower	Upper	Midpoint	Percent of global estimate midpoint	Percent based on specific species/genus weight data (by tonnage)	Lower	Upper	Top species for country (by estimate midpoint)	Any welfare law for farmed fish slaughter
1	Peru	6,192	200	570	390	24%	98%	11	31	Anchoveta (=P. anchovy)	Y
2	China	10,230	130	220	170	11%	56%	48	78	Marine fishes nei	N
3	Chile	2,839	58	150	110	7%	97%	19	49	Anchoveta (=P. anchovy)	Y
4	Indonesia	4,906	62	140	100	6%	67%	35	79	Stolephorus anchovies nei	Y
5	India	3,743	55	100	78	5%	30%	37	68	Cyprinids nei	Y
6	Denmark	814	53	56	54	3%	100%	15	15	Sandeels (=Sandlances) nei	Y
7	Morocco	1,045	39	43	41	3%	92%	24	27	European pilchard (=Sardine)	N
8	Myanmar	1,759	26	54	40	2%	0.4%	33	67	Marine fishes nei	N
9	Vietnam	1,916	26	53	39	2%	4%	36	74	Marine fishes nei	Y
10	Philippines	1,989	25	52	39	2%	77%	38	80	Stolephorus anchovies nei	Y
11	Japan	3,127	31	44	38	2%	86%	70	100	Japanese anchovy	N
12	Thailand	1,791	24	48	36	2%	41%	37	76	Marine fishes nei	N
13	Russian Fed	3,809	17	40	29	2%	94%	95	219	European sprat	N
14	Norway	2,281	17	35	26	2%	100%	65	135	Sandeels (=Sandlances) nei	Y
15	Bangladesh	1,415	17	34	25	2%	26%	41	84	Freshwater fishes nei	N
16	Turkey	409	12	38	25	2%	92%	11	33	European anchovy	Y
17	Malaysia	1,190	16	32	24	1%	46%	37	77	Marine fishes nei	Y
18	Iceland	1,403	9.5	29	19	1%	100%	48	147	Capelin	Y
19	South Africa	625	15	19	17	1%	98%	33	43	Southern African anchovy	Y
20	South Korea	1,170	14	19	16	1%	84%	63	83	Japanese anchovy	N
21	USA	3,847	8.4	18	13	1%	97%	213	457	Gulf menhaden	N
22	Mexico	1,203	9.9	16	13	1%	83%	75	122	California pilchard	Y
23	Sweden	230	11	12	12	1%	100%	18	20	European sprat	Y
24	Cambodia	481	7.1	15	11	1%	0%	33	68	Freshwater fishes nei	?
25	Poland	191	9.0	10	9.6	1%	91%	19	21	European sprat	Y
26	Tanzania	360	6.8	11	9.0	1%	69%	32	53	Silver cyprinid	N
27	Ukraine	181	6.3	11	8.4	1%	71%	17	29	European sprat	Y
28	Uganda	383	6.3	9.9	8.1	1%	79%	39	61	Silver cyprinid	Y
29	Namibia	491	1.6	14	7.9	0.5%	98%	34	301	Cape horse mackerel	Y
30	Spain	866	4.8	10	7.4	0.5%	86%	87	179	European pilchard (=Sardine)	Y
	Total above	**60,887**	**920**	**1,900**	**1,400**	**87%**	70%	32	66		
	Others	16,462	140	270	200	13%					
	Total	**77,348**	**1,100**	**2,200**	**1,600**	**100%**	71%	36	73		
	EU27	4,429	110	140	130	8%	94%	31	39	European sprat	Y

Footnote: Table shows average annual estimated number ranges for wild-caught finfishes (2000–2019), for the top 30 countries, ranked by descending estimate midpoint. The top 30 countries account for 87% of global numbers. Peru, China, Chile and EU27 countries combined (also shown) account for nearly 50%. Estimated numbers are rounded to 2 significant figures. Estimates are based on capture production tonnages (landings) reported by the FAO (2021a) and estimated mean individual weights (EMWs) for species. For tonnages where no EMW was obtained for the category of species, generic estimated mean weights (GEMWs) extrapolated from EMW estimates were used. Country estimates are expected to be more reliable where the percentage (shown) based on data for the same or closely related species (EMWs and GEMWs for the same genus) is higher. Similar estimates for all countries are shown in Supplementary Table S5. Note that, for any country, the top species (shown) may represent only a small percentage of total numbers caught in cases where no single category of species dominates capture numbers. For scientific names of species shown, see Table 4, except for California pilchard (*Sardinops caeruleus*). Of the countries shown, a total of 19 countries, together with all other EU27 countries, have some national animal welfare law covering fish slaughter in the context of fish farming (Table S10 in the Supplementary material), usually a requirement to avoid causing unnecessary suffering. Cambodian law was not analysed.

### Sensitivity analysis

The results for alternative estimates A1 to A6, averaged annually for 2000–2019, were similar to that for the main estimate of 1,100–2,200 billion (1.1–2.2 × 10^12^) fishes (Table 6).

**Table 6.** Estimated number ranges for wild-caught finfishes, in seven alternative estimates

		Numbers in billions (× 10^9^)			
Estimate	Period	Lower	Upper	Midpoint	Number of fish weights used
Main	2000–2019	1,100	2,200	1,600	691
A1	2000–2019	1,000	2,300	1,600	698
A2	2000–2019	970	2,300	1,600	742
A3	2000–2019	1,100	2,200	1,600	708
A4	2000–2019	1,100	2,200	1,600	676
A5	2000–2019	960	2,300	1,600	805
A6	2000–2019	990	2,100	1,600	691
A7	2000–2019	1,000	2,600	1,800	701
	1999–2007	1,100	2,700	1,900	

Footnote: Table shows results for each alternative estimate of global wild-caught finfish number ranges, compared with the main one, averaged annually. Numbers are estimated from capture production tonnages (landings) reported by the FAO (2021a) using estimated mean individual weights for species. A1 to A6 show a similar result. A1 to A5 differ only in the data ranking system (Table 1), which determines the selection of fish weights (from those collected using internet searches) used. A6 assumes market weights are headed and gutted. A7 uses alternative calculations for multi-species tonnages, giving a wider range. Estimated numbers are rounded to 2 significant figures.

In A7, which uses a different method for multi-species categories, a total of 153 multi-species EMWs were obtained. A7 produced a wider range of 1,000–2,600 billion (1.0–2.6 × 10^12^), on average annually for 2000–2019 (Table 6). When averaged for the period 1999–2007, for which numbers were previously estimated (Mood & Brooke 2010), A7 produced a range of 1,100–2,700 billion (1.1–2.7 × 10^12^) (Table 6).

### Estimates for reduction fisheries

Estimated numbers of fishes reduced to fishmeal and oil in 2010 totalled 490–1,100 billion (4.9 × 10^11^–1.1 × 10^12^) fishes (Table S6 in the Supplementary material), with a midpoint of 810 billion. These represented 56% of the 2010 total estimate of 1,000–1,900 billion (1.0–1.9 × 10^12^), by estimate midpoint. The overall estimated mean weight for these reduced fishes was 15–33 g. Estimated numbers of fishes caught for reduction in 2001–2006 totalled 430–1,000 billion fishes (4.3 × 10^11^–1.0 × 10^12^) (Table S7 in the Supplementary material), with a midpoint of 730 billion. Their overall estimated mean weight was 13–30 g.

Using the estimates obtained by species for 2000–2019 (Supplementary Table S3), the overall estimated mean weight for the top 20 species classified as ‘forage fish’ by Froehlich *et al.* (2018), discussed earlier, was obtained as 18–39 g. Based on this mean weight range, a decrease in ‘forage fish’ capture by six million tonnes, as might be required by more precautionary management measures according to Froehlich *et al.* (2018), is estimated to comprise 150–330 billion (1.5–3.3 × 10^11^) individuals, with a midpoint 240 billion. This represents 15% of the total estimated annual numbers for 2000–2019 of 1,100–2,200 billion (Table 4) by estimate midpoint.

### Estimates for certified fisheries

Estimated numbers of fishes certified by FOS in 2014, and MSC in 2015, based on certified tonnages reported in Potts *et al.* (2016), are as follows (Tables S8 and S9 in the Supplementary material). FOS-certified anchoveta capture comprised 110–310 billion (1.1–3.1 × 10^11^) fishes with a midpoint of 210 billion. Other FOS-certified finfish capture comprised 9.2–39 billion (9.2 × 10^9^–3.9 × 10^10^) fishes with a midpoint of 24 billion. MSC certified capture comprised 7.8–26 billion (7.8 × 10^9^–2.6 × 10^10^) fishes with a midpoint of 17 billion.

### Analysis of fish protection law

Of the top 30 producing countries for wild-caught finfishes, 19 have some general animal welfare law that, in principle, covers farmed fishes at slaughter (Table 5), of which only Norway (Norwegian Government 2018) and Iceland (Icelandic Government 2018) have fish-specific welfare codes, according to this analysis (Table S10 in the Supplementary material). Combining these countries with the remaining EU28 countries (EU27 countries plus the UK), gave a list of 43 countries with such welfare law. Fishes caught by these 43 countries, on average annually in 2000–2019, totalled 630–1,400 billion fishes, or 64% of the total estimate of 1,100–2,200 billion (Table 4) by estimate midpoint. Estimated numbers caught by top countries where welfare laws did not cover farmed fish slaughter, or for which this was unclear (Cambodia), totalled 320–540 billion, or 27% of the total estimate midpoint. Estimated numbers caught by the remaining countries that were not analysed, i.e. countries not in the top 30 nor in the EU28, totalled 100–210 billion, or 10% of the total estimate.

### Estimates for other vertebrates

For turtles and frogs, farmed production totalled 374,336 and 123,845 tonnes, respectively, in 2019 (FAO 2021b), while capture production totalled 444 and 1,147 tonnes, respectively (FAO 2021a). Estimated numbers were as follows: 500–2,500 million farmed frogs, 250–370 million farmed turtles, 4.6–23 million wild frogs and 22–890 thousand wild turtles (Table 7).

**Table 7.** Vertebrates killed globally for food, etc (2019)

Taxa	Production tonnage	Percent production tonnage %	Numbers	Percent numbers %	Source
Farmed animals:					
Finfishes	56,000,000	12	*120,000,000,000*	7.5	1, 2
Birds	130,000,000	28	77,000,000,000	4.7	3
Mammals	200,000,000	43	3,500,000,000	0.2	3
Frogs	120,000	<1	*1,500,000,000*	0.1	1,4
Turtles	370,000	<1	*310,000,000*	<0.1	1,5
Wild-caught aquatic animals (reported):					
Finfishes	79,000,000	17	*1,400,000,000,000*	87.5	6,7
Frogs	1,100	<1	*14,000,000*	<0.1	6,4
Crocodiles	NA		1,200,000	<0.1	6
Turtles	440	<1	*460,000*	<0.1	6,8
Marine mammals	NA		100,000	<0.1	6
Wild-caught terrestrial animals:	2,000,000	<1	NA		9
Total for above	**480,000,000**	**100**	***1,700,000,000,000***	**100**	
Unreported capture of wild finfishes, crustaceans & molluscs:					
Unreported landings	16,000,000	3	NA		10
Discards	8,400,000	2	NA		10

Footnote: Table shows vertebrates killed for food (and feed etc in the case of finfishes) in tonnages and numbers from, or based on, FAO data, together with unreported fishery capture. Numbers shown in italics indicate estimates (midpoints) obtained from other sources, based on FAO production tonnages. NA = currently unquantified. While wild-caught finfishes comprise only 17% of total FAO reported vertebrate production by tonnage, they represent an estimated 87.5% by numbers, due to their smaller average weight. These totals exclude fish numbers caught in discarded catch and unrecorded landings, hunted terrestrial animal numbers and other unrecorded mortalities in all taxa. Tonnages and numbers are rounded to 2 significant figures.

Sources: 1. FAO (2021b). 2. Mood *et al.* (2023). 3. FAO (2023a). 4. Farmed and wild-caught frog numbers are here estimated from tonnage, assuming a mean weight of 50–250 g based on Cagiltay *et al*. (2014) and Zhu *et al*. (2021). 5. Farmed turtle numbers are here estimated from tonnage, assuming a mean weight of 1.0–1.5 kg based on FAO (2023d). 6. FAO (2021a). 7. Present study. 8. Wild-caught turtle numbers are here estimated from tonnage, assuming a mean weight of 0.5–19.8 kg based on Nijman (2010) and Pham *et al.* (2013). 9. FAOSTAT (FAO 2023a) reports that 1.98 million tonnes of ‘game meat’ were produced but does not report the taxa or corresponding numbers of animals. This source reports numbers of production animals only for domesticated species and for hares, which are included in the same category as rabbits. It is here assumed that FAOSTAT production numbers for birds and mammals virtually all relate to farmed animals. 10. Pauly *et al.* (2020).

## Discussion

Based on recorded capture, the present study estimates that between 1,100 and 2,200 billion (1.1–2.2 × 10^12^) fishes were caught from the wild each year (Table 4), on average, during 2000–2019; with annual variation (Figure 1). Estimated fish numbers mainly relate to capture from the marine environment (Figure 3).

The top species is anchoveta (Table 4). Numbers are high due to its high capture production tonnage, second only to ‘Marine fishes nei’, and its small individual weight (Table 4). This species is caught in the Southeast Pacific (FAO 2021a) by Peru and Chile, which are among the top three producing countries by estimated numbers (Table 5). Anchoveta population dynamics are strongly influenced by environmental variability (Oliveros-Ramos *et al.* 2021). Patterns of anchoveta capture production have had, and will have, an important influence on global fish numbers caught (Figure 2, Supplementary Table S4). Note, however, that the inter-year differences in anchoveta numbers shown (Figure 2, Supplementary Table S4), being based on estimate midpoints, do not allow for any changes in mean weight, e.g. that might occur with changes in the proportions of juveniles in catches (Gutierrez *et al.* 2022).

Anchoveta, and most species in the top marine single-species categories (name not ending with ‘nei’) (Table 4), are mostly caught by purse seines and/or pelagic trawls (Pauly *et al*. 2020). Many of the top species are caught usually, or sometimes, for reduction to fishmeal and oil (Table 4), for which total numbers have here been estimated (as discussed later).

Annual estimated numbers for the period 1999–2007, for which fish numbers have previously been estimated by Mood and Brooke (2010), averaged 1,100–2,400 billion (1.1–2.4 × 10^12^), in the present study (Supplementary Table S4). This is slightly narrower than the earlier estimate of 970–2,700 billion for the same period (Mood & Brooke 2010). The lower figure has increased, mainly due to the new data. The upper figure has reduced, mainly due to a change in the method for multi-species tonnages, aimed at producing a more precise estimate (narrower range). Alternative estimate A7 used similar estimating methods for such tonnages to Mood and Brooke (2010) and obtained a similar range of 1,100–2,700 billion (1.1–2.7 × 10^12^) for 1999–2007 (Table 6).

The present study’s fish numbers are best estimates, calculated from FAO fisheries capture statistics, together with wild fish capture or market weight data from various sources. Some accuracy issues relating to FAO capture production tonnages, and the mean weight estimates derived herein, are discussed below.

FAO capture statistics are themselves estimates, and not all wild-caught fishes are included. The FAO capture production database omits the portion of the catch that is discarded at sea and catches from illegal, unreported or unregulated (IUU) fisheries, which are both inherently difficult to estimate (FAO 2018). FAO fishery capture statistics also exclude mortalities in fishes caught, or impacted by, fishing gear but not retrieved onboard. These include fishes that die following escape from nets or actively avoiding them, or following deliberate release prior to bringing the catch onboard, or in capture by lost or discarded fishing gear (‘ghost fishing’) (International Council for the Exploration of the Sea [ICES] 2005; Pérez Roda *et al.* 2019).

The FAO actively assures the quality of its fishery capture statistics as far as possible (FAO 2018), aside from these exclusions. However, the following sources of inaccuracy remain:

- Often national data submitted are incomplete, inconsistent or do not comply with international reporting standards; the FAO estimates missing data (FAO 2020);
- Some sectors are under-reported, including small-scale marine fisheries (Pauly & Zeller 2016), inland fisheries (Funge-Smith 2018; FAO 2020) and recreational fisheries (Funge-Smith 2018; FAO 2021c);
- Over-reporting by some countries such as China, for which global capture production was revised downward by around 2% for 2006 and 2016 (FAO 2020);
- Incorrect conversion of processed weights to live weight equivalents (Garibaldi 2012); and
- Fishes caught from enhanced fisheries, e.g. fisheries stocked with fingerlings from aquaculture or enhanced with habitat management, are often incorrectly reported as aquaculture rather than capture production (Funge-Smith 2018).

Pauly and Zeller (2016) estimated the marine capture tonnages missing from FAO statistics, including under-reported small-scale fishing capture, discards and illegal catch, using data from a wide variety of sources in a process called ‘catch reconstruction’. These authors estimated that, in 2010, global marine fish and invertebrate capture (excluding corals and sponges) comprised an additional 32 million tonnes compared with that reported by the FAO (Pauly & Zeller 2016 [in Table S1 of the Supplementary material]). This reconstructed catch excludes fishes killed by fishing gears but not retrieved, due to lack of data. An FAO study estimated that discards of animals (excluding corals and sponges) in global commercial marine capture totalled 9.1 million tonnes annually for 2010–2014 (Pérez Roda *et al.* 2019). According to reconstructed fish and invertebrate catch data obtained from the Sea Around Us website (Pauly *et al.* 2020), global discards and unreported landings in 2019 totalled 8.4 million tonnes and 16.1 million tonnes, respectively.

Another quality issue in FAO capture production tonnages is the extent to which species are reported separately or aggregated into multi-species categories with less species information. The taxonomic resolution of reported catches, like reliability, varies between countries (Pauly & Zeller 2017). Categories ‘Marine fishes nei’ and ‘Freshwater fishes nei’ (discussed below), with no specified taxonomic order and therefore comprising potentially very diverse finfish species, together represent 19% of total tonnage (Table 4). This capture is predominantly reported by Asian countries (FAO 2021a), and ‘Marine fishes nei’ includes capture from China’s distant-water fishing fleet (FAO 2018, 2020). According to the FAO (2022), marine catches have generally more complete data available by species than do inland captures, with ‘Freshwater fishes nei’ accounting for around half of global reported inland fishery capture in recent years (FAO 2022).

Aside from the omitted capture and other potential inaccuracies in FAO capture production tonnages discussed above, it is presumed that the most reliable estimates of numbers are those for species categories with an EMW, or a GEMW for the same genus, since these are based on fish weight data for the same or closely related species. These represented 72% of the estimate by midpoint (Table 2). The least reliable estimates are those for species categories for which the taxonomic order is not known, virtually all relating to the categories ‘Marine fishes nei’ and ‘Freshwater fishes nei’. These were therefore based on GEMWs calculated at the level or class or all classes combined and represented 21% of the estimate midpoint (Table 2).

The most reliable fish weights used to obtain EMWs are likely to be those based on fishery capture weights, including 111 mean weights obtained from research articles on studies that sampled individuals caught in commercial and artisanal fishing. Due to the limited data on fishery capture weights, other data, such as marketing weights and survey fishing weights, have also been used.

The sensitivity analysis showed that, when several alternative rules were employed for selecting data to include in each EMW, similar overall results were obtained for the average annual estimate (Table 6). Weights from a marketing or food-related website have been assumed to represent the whole fish, unless otherwise stated, which could mean that some corresponding EMWs are under-estimated and numbers over-estimated. The sensitivity analysis (A6) showed that reversing this assumption produced a similar, though slightly lower, total estimate (Table 6), suggesting the potential effect is not great.

The potential variability of fish capture weight, for any species, increases the difficulty in estimating the global mean weight. Examples of variation between population, capture method and over time are as follows. Alemany and Alvarez (1993) found that that sardine (*Sardina pilchardus*), also called ‘European pilchard’ (Table 4), in the Western Mediterranean could be grouped into two separate populations, differing in length. The mean length for the oldest age class was above 20 cm for sardine located in the Alboran Sea and Gulf of Vera, and about 18 cm for sardine located further northeast, in Alicante, Valencia and Gulf of Lions (Alemany & Alvarez 1993). The capture size range of yellowfin tuna (*Thunnus albacares*), in the Western and Central Pacific Ocean, varies depending on fishing method: 20–70 cm and 90–160 cm when caught by pole and line and longline, respectively, the difference due to the size of hooks used and depth of fishing, with the full range caught by purse seine (Allain *et al*. 2016). The annual average weight of skipjack tuna (*Katsuwonus pelamis*) caught by purse seine and pole and line vessels in the Eastern Pacific Ocean between 2011–2016 ranged between 1.8 and 2.5 kg (Inter-American Tropical Tuna Commission [IATTC] 2017). One effect of fishing pressure, more generally, is that over time, it can change the demographic composition of a fish population toward a dominance of younger and smaller fish (Enberg *et al*. 2009).

### Fish number estimates for reduction fisheries

It is here estimated that 490–1,100 billion (4.9 × 10^11^–1.1 × 10^12^) finfishes were processed into fishmeal and oil in 2010 (Supplementary Table S6), based on reconstructed marine capture destined for reduction totalling 16.6 million tonnes (Cashion *et al.* 2017 [in Table S2 of the Supplementary material]). This comprises 260–600 billion fishes of identified species and, by extrapolation, 230–530 billion fishes of unidentified species. According to Péron *et al.* (2010), fishmeal and fish oil are generally derived from small pelagic fish species. Consequently, the proportion of fish numbers used for this purpose is greater than the proportion by tonnage. Cashion *et al*. (2017) report a growing diversity of species used for reduction, in addition to the main species so used, sourced largely from Asian trawl fisheries. This estimate assumes that all capture for reduction comprises finfishes, except for an estimated tonnage of Antarctic krill (Supplementary Table S6), which is partly used to make meal (Katevas 2014) and not included in the estimate.

Since this estimate is based on reconstructed marine capture that was destined for reduction, it excludes bycatch from reduction fisheries that was not so used, either discarded overboard or landed. This estimate also excludes fishes used for reduction in the form of trimmings, i.e. by-products from use as food (FAO 2020); fishes caught in inland fishing, such as silver cyprinid (*Rastrineobola argentea*), which is also used to make fishmeal (Kubiriza *et al*. 2021); and fishes used as direct feed or bait (FAO 2020).

FAO data suggest that total capture production destined for reduction has remained at a similar level since then, at least by tonnage; averaging 15.8 million tonnes annually between 2011–2019 compared with 15.0 million tonnes in 2010 (FAO 2021c).

This estimate of 490–1,100 billion fishes destined for reduction in 2010 (Supplementary Table S6) is comparable to separate similar estimates, averaged annually, of 430–1,000 billion fishes for 2001–2006 (Supplementary Table S7) and 460–1,100 billion fishes for 2007–2016 (Mood & Brooke 2019b) (not peer reviewed).

### Fish number estimates for certification schemes

Potts *et al.* (2016) reported that the virtual entirety of the Peruvian anchoveta population is certified by FOS. Besides this most numerous species (Table 4), estimated numbers of other fish species caught in capture certified by the FOS and MSC certification schemes combined (Supplementary Tables S8 and S9), annually in 2014–2015, totalled 17–66 billion (1.7–6.6 × 10^10^), with a midpoint of 42 billion. This equates to 3% of total estimated numbers for 2014 or 2015 (Supplementary Table S4). This assumes that certified capture numbers were similar between the two years and assumes no double certification, rates of which Potts *et al*. (2016) believed were negligible. These FOS and MSC estimates are based on certified tonnages, as reported by Potts *et al.* (2016), and EMWs/GEMWs from the present study, with some assumptions regarding species composition and some adjustments for consistency with FAO capture production tonnages (Supplementary Tables S8 and S9).

### Animal welfare implications

The number of animals affected is one important measure of the extent of any welfare issue and can help identify priorities for research and policy efforts. This estimate of 1,100–2,200 billion (1.1–2.2 × 10^12^) finfishes caught from the wild in recorded global capture, on average annually during 2000–2019 (Table 4, Supplementary Table S3), indicates the very large numbers of animals that could benefit from improved fish welfare during wild capture and killing. Estimated fish numbers are high for many individual species (Table 4, Supplementary Table S3) and countries (Table 5 and Supplementary Table S5).

The estimate of 980–1,900 billion (9.8 × 10^11^–1.9 × 10^12^) wild-caught finfishes in 2019 (Table 3) exceeds, by an order of magnitude, 81 billion farmed birds and mammals (FAO 2023a) and an estimated 78–171 billion farmed fishes (Mood *et al.* 2023), killed for food that same year. Based on these and other FAO data, including estimated frog and turtle numbers (see *Results*) but excluding hunted terrestrial animal numbers, wild-caught finfishes represented an estimated 87.5% of vertebrates killed for consumption in 2019 (Table 7) which, in the case of wild finfishes, means mainly for direct human consumption or reduction to fishmeal and oil (FAO 2021c).

Anatomical, physiological, pharmacological, and behavioural evidence suggests that fishes feel pain, including changes in motivational states following painful events observed in rainbow trout (*Oncorhynchus mykiss*), goldfish (*Carassius auratus*) and zebrafish (*Danio rerio*) (Sneddon *et al.* 2014; Sneddon & Leach 2016). Brown (2015) argues that, since little is known about most fish species, and while it is hard to generalise between diverse groups, we must assume that the capabilities that have been revealed by model taxa are likely to be exemplary of teleosts as a whole. Teleosts represent 99.97% of estimated numbers for all fishes excluding those reported in miscellaneous ‘fishes nei’ categories. Capacity to feel pain may also extend to other taxonomic groups; questions such as ‘does the lowly hagfish experience the same pain as the trout?’ are not easy questions (Silverman 2008). We consider that a precautionary approach that assumes all finfishes (and some invertebrates [see below]) are sensitive to pain is appropriate, to begin to address the likely severe welfare impacts on large numbers of animals. Whether or not intelligence is necessary for a capacity to feel pain, behavioural and cognitive sophistication has been demonstrated in a range of fish species (Brown 2015), including frillfin goby (*Bathygobius soporator*) (Aronson 1971), Siamese fighting fish (*Betta splendens*) (Oliveira *et al*. 1998), grouper (*Plectropomus pessuliferus*) and giant moray eel (*Gymnothorax javanicus*) (Bshary *et al*. 2006), and cleaner wrasse (*Labroides dimidiatus*) (Salwiczek *et al*. 2012), as well as manta ray (*Manta birostris*) (Ari & D’Agostino 2016), which is an elasmobranch (non-teleost) species.

Captured fishes experience a range of stressors (Breen *et al.* 2020) during a process that may take several to many hours (Wassenberg *et al*. 2001; Savina *et al*. 2016; Trygg Mat Tracking & IMCS Network 2021). Those that are alive when retrieved onboard are generally not stunned and die from live gutting and/or asphyxiation in air or ice water (van de Vis & Kestin 1996; Metcalfe 2009; Anders *et al.* 2019; Breen *et al.* 2020), for which the time taken until loss of consciousness may be up to one or more hours (van de Vis & Kestin 1996; Lambooij *et al.* 2012). Since welfare is likely to be very poor for often extended periods, and such large numbers are caught, the capture of wild fishes is a major animal welfare issue for which there is an ethical obligation to improve practices. Humane slaughter technologies developed for aquaculture may be applicable in commercial fisheries (Huntingford & Kadri 2009), such as the prototype stunner tested for use on wild-caught cod and haddock by Lambooij *et al.* (2012).

Note that these estimates for wild-caught fishes exclude unrecorded landings and fishes discarded overboard (Table 7); other unrecorded fish mortalities, discussed earlier; and invertebrate animals. These include, respectively, 5.2 and 3.9 million tonnes of decapods and cephalopods, caught annually in 2000–2019 (FAO 2021a), comprising, respectively, 171 and 42 categories of species in 2019, for which similar principles for welfare apply since there is evidence these can also experience pain (Birch *et al*. 2021; Crook 2021; Elwood 2021). The FAO (2014) recognises that ethical considerations are a major reason for protecting animal welfare during slaughter and that these apply equally to aquatic animals in fisheries and aquaculture as to livestock and poultry animals, according to guidelines on the SAFA framework.

It would aid welfare assessment if the FAO collected and reported data on fish numbers (as they do for other taxa [see Table 7]) or published mean capture weights that would enable numbers to be calculated. This would help encourage the recognition of fishes as individual wild animals that need protection through conservation and welfare measures, and not merely commodities as has been a traditional view (Pauly & Zeller 2017). Mean weight data could also be useful for assessment of fish populations, since fish mean weights can change over time with fishing pressure and may affect the ability to reproduce (Charbonneau *et al*. 2019). Improving FAO statistics to be more informative regarding the sustainability of capture, such as including discards (Pauly & Zeller 2017) and improving the species breakdown (Liang & Pauly 2017; Blasco *et al.* 2020), would likewise benefit welfare assessment, as would more information on the fishes used for direct feed and bait (sometimes used live and impaled on hooks [Gregory 1998; Robertson *et al.* 2010]). Fish capture so used is not reported separately but included in 4.0 million tonnes of total landings that were used for purposes besides food and reduction in 2019 (FAO 2021c), but this excludes fishes used for bait that are not landed (FAO 2021c).

Improving the sustainability of fisheries, through more precautionary catch limits (Pauly *et al.* 2002) and by reducing incidental catch (FAO 2011), could potentially benefit fish welfare by reducing the numbers caught. With 35.4% of fishery stocks fished at biologically unsustainable levels in 2019, according to the FAO’s assessment (FAO 2022), it is a goal target of the United Nations (UN) to improve sustainability by lowering catches, at least to levels that can produce maximum sustainable yield, and ending illegal fishing (UN 2023). Targeting larger individuals of a species, to let fishes breed and grow to an optimal size before capture, would reduce the impact of fishing on fish stocks (Froese *et al.* 2016) and could decrease capture numbers. Setting catch levels low enough to protect species connected by predator-prey relationships helps to conserve biodiversity and prevent the phenomenon of ‘fishing down marine food webs’, whereby depletion of predator species results in increasing numbers of smaller low-trophic level fishes in catches (Pauly *et al.* 2002). Use of artificial fishing baits, as alternatives to the use of fishes for bait (Dellinger *et al.* 2016; Karunanithi *et al.* 2018; Masilan & Neethiselvan 2018), might enable fewer such fishes to be caught, benefiting conservation and welfare.

It has been argued that eating small fishes, lower down the food chain, would have benefits for nutrition, food safety and the environment (Corliss 2023). This could, in principle, increase capture numbers, unless these fishes are diverted from other uses. The direct human consumption of small low-trophic level fishes has been recommended as an alternative to their use for fishmeal and oil (Cashion *et al.* 2017; Xia *et al.* 2023).

Midpoints of estimates based on 2010 data (see *Results*) suggest that around half of wild-caught finfish numbers are destined for reduction to fishmeal and oil, of which, respectively, 70 and 73% are used for aquaculture feeds (Mallison 2017), though their inclusion rates (as a percentage of feed) show a clear downward trend (FAO 2020). Although fishes used for fishmeal and oil are small (see *Results*), it should not be assumed that their capacity to suffer is less than for any other teleosts. These are much larger than the zebrafish, weighing less than one or two grams (e.g. Rey *et al.* 2015), for which evidence for sensitivity to pain has been found (Sneddon *et al.* 2014). The development of gentler capture and humane slaughter for these species could benefit many fishes. It may also be possible to develop strategies to reduce capture numbers, consistent with fisheries’ management objectives, since these fishes play an important role in marine food webs (Shannon & Waller 2021). According to Froehlich *et al.* (2018), implementation of more precautionary ecosystem- and conservation-based catch limits might reduce capture of the small marine fish species that are used for reduction (‘forage fish’) by six million tonnes per year, and it is here estimated that this would decrease total capture numbers by 15% (see *Results*).

The estimated high numbers of fishes caught for reduction could substantially increase if efforts to develop mesopelagic fishing overcome current technical and economic obstacles, catching finfishes such as lanternfish (Myctophidae) species weighing 0.8–3.3 g (Irigoien *et al.* 2014), from a total estimated biomass of 2–19.5 billion tonnes (Sobradillo *et al.* 2019), with total population numbers potentially measuring in the quadrillions (× 10^15^).

### Welfare codes

Despite the predominance of finfishes in vertebrate numbers used (directly or indirectly) for food (Table 7), little if any welfare regulation exists that constrains how they are handled or killed in wild-capture marine fisheries (Metcalfe 2009). There is legal protection for wild-caught freshwater fishes in Swiss inland fisheries (Swiss Federal Council 2020), potentially benefiting an estimated 5.3–12 million fishes (Supplementary Table S5), and possible welfare requirements for captured fishes held for killing later, e.g. in restaurants, as in New Zealand (New Zealand Government 2022). Existing law on captive wild animal welfare might be interpreted to provide welfare protection for wild-caught fishes, on which basis the impaling of live fishes on hooks for use as fishing bait is prohibited in Sweden (Jordbruksverket 2011).

For many countries, accounting for at least 64% of estimated wild-caught finfish numbers, a need to protect the welfare of fishes during slaughter is recognised in law (Table 5) in the context of aquaculture, which could logically be extended to wild fishes. It is preferable that codes are fish-specific and species-specific since, as has been shown by widespread non-adherence to OIE guidelines in the farmed fish slaughter practices of some EU countries (European Commission 2017), general statements to minimise suffering cannot be relied upon to ensure use of humane slaughter methods.

Standards of certification schemes, and those of food businesses, could potentially protect the welfare of wild-caught fishes, as they increasingly do in the context of aquaculture, including for slaughter (Mood *et al.* 2023). The MSC and FOS, the two main global certification schemes for capture fisheries in 2015 (Potts *et al.* 2016), aim to ensure that a certified fishery is sustainable and managed in accordance with laws and regulations (FOS 2020; MSC 2022). Aside from prohibiting shark-finning, neither scheme currently requires more humane capture and killing (FOS 2020; MSC 2022) and by excluding fish welfare, the schemes do not currently match sustainability guidelines (FAO 2014). Many billions of fishes could potentially benefit from such requirements (see *Discussion*).

## Conclusion

We have estimated that 1,100–2,200 billion (1.1–2.2 × 10^12^), or 1.1–2.2 trillion, finfishes were caught, on average, each year, in recorded global fisheries capture during 2000–2019. This excludes unrecorded fish capture and other wild-caught animals such as decapods and cephalopods. Since fishes generally suffer very poor welfare during capture and killing, and in such large numbers, fishing is a major animal welfare issue. We recommend the FAO collects and publishes statistics for wild fish capture in numbers, as well as tonnages, to facilitate animal welfare assessment. The welfare of very large numbers of fishes would potentially be improved by reducing the duration of capture and time out of water; gentler capture and humane slaughter methods; and by reducing numbers caught through policies also designed to improve sustainability.

## Supporting information

Mood and Brooke supplementary material 1 Mood and Brooke supplementary material

Mood and Brooke supplementary material 2 Mood and Brooke supplementary material

Mood and Brooke supplementary material 3 Mood and Brooke supplementary material

Mood and Brooke supplementary material 4 Mood and Brooke supplementary material

Mood and Brooke supplementary material 5 Mood and Brooke supplementary material

Mood and Brooke supplementary material 6 Mood and Brooke supplementary material

Mood and Brooke supplementary material 7 Mood and Brooke supplementary material

Mood and Brooke supplementary material 8 Mood and Brooke supplementary material

Mood and Brooke supplementary material 9 Mood and Brooke supplementary material

Mood and Brooke supplementary material 10 Mood and Brooke supplementary material

## Supplementary material

The supplementary material for this article can be found at http://doi.org/10.1017/awf.2024.7.
