# Supplementary material for: Estimating global numbers of fishes caught from the wild annually from 2000 to 2019
Source: Anim Welf. 2024 Feb 8;33:e6. doi: 10.1017/awf.2024.7 (PMC10951671; doi:10.1017/awf.2024.7)
Supplement: Mood and Brooke supplementary material 1 — Mood and Brooke supplementary material [file S0962728624000071sup001.pdf]

Anchoveta catch 1991-2000

Year	Anchoveta capture production (landings) in tonnes	Period	10-year average annual anchoveta capture in tonnes	10-year average annual anchoveta capture as percentage of 1991-2000 average
1991	4,017,106			
1992	6,157,269			
1993	8,482,463			
1994	12,520,611			
1995	8,644,576			
1996	8,863,714			
1997	7,685,098			
1998	1,729,064			
1999	8,723,265			
2000	11,276,357	1991-2000	7,809,952	100%
2001	7,213,077	1992-2001	8,129,549	104%
2002	9,702,614	1993-2002	8,484,084	109%
2003	6,203,751	1994-2003	8,256,213	106%
2004	10,679,338	1995-2004	8,072,085	103%
2005	10,244,166	1996-2005	8,232,044	105%
2006	7,007,157	1997-2006	8,046,389	103%
2007	7,611,858	1998-2007	8,039,065	103%
2008	7,419,295	1999-2008	8,608,088	110%
2009	6,910,467	2000-2009	8,426,808	108%
2010	4,205,979	2001-2010	7,719,770	99%
2011	8,319,597	2002-2011	7,830,422	100%
2012	4,692,855	2003-2012	7,329,446	94%
2013	5,674,036	2004-2013	7,276,475	93%
2014	3,140,029	2005-2014	6,522,544	84%
2015	4,310,015	2006-2015	5,929,129	76%
2016	3,192,476	2007-2016	5,547,661	71%
2017	3,922,746	2008-2017	5,178,750	66%
2018	7,044,950	2009-2018	5,141,315	66%
2019	4,248,852	2010-2019	4,875,153	62%

Average 1999-2007	8,740,176
Average 2000-2019	6,650,981

This table shows anchoveta (*Engraulis ringens*) capture production tonnages (landings) reported by the FAO (2021a) for the years 1991-2019. During the period 2000-2019, annual capture ranged between 3.1 million tonnes (2014) and 11 million tonnes (2000), to 2 significant figures. When the data for 1991-2019 is averaged over rolling 10-year periods, average annual capture ranged from 4.9 million tonnes for the period 2010-2019, to 8.6 million tonnes for 1999-2008.
