# Supplementary material for: Estimating global numbers of fishes caught from the wild annually from 2000 to 2019
Source: Anim Welf. 2024 Feb 8;33:e6. doi: 10.1017/awf.2024.7 (PMC10951671; doi:10.1017/awf.2024.7)
Supplement: Mood and Brooke supplementary material 2 — Mood and Brooke supplementary material [file S0962728624000071sup002.pdf]

Types of fish size reference used to estimate mean capture weights

Reference type		No. of reported fish sizes obtained								Total[ii]	Percent of total
		Average/ mean weight	Usual/ modal weight	Simple weight range	Common/ typical weight	Survey mean weight[i]	Average/ mean length	Usual/ modal length	Common/ typical length		
1	Research article	178	7	8		33		2		228	33
2	Seafood marketing & industry	54	12	56	1		2	1		126	18
3	Government & inter-government	99	4	4	2	10				119	17
4	Recreational fishing	57	2	4	4					67	10
5	Research institute, university, science centre & museum	39	3	1		4				47	7
6	FAO factsheet/report	8	6	2		2	3		4	25	4
7	NGO	19	1	1	1			1		23	3
8	FishBase (Froese & Pauly 2021)	1							20	21	3
9	Misc fish biology	8	2						1	11	2
10	Misc food	10	1							11	2
11	University student thesis/project	8				2				10	1
12	Diving	1			1					2	<1
13	Other periodical	1								1	<1
	Total	**483**	**38**	**76**	**9**	**51**	**5**	**4**	**25**	**691**	100
	Percent of total	70	5	11	1	7	1	1	4	100	

[i] Mean weights are categorised as survey mean weights when obtained from survey fishing, e.g. survey trawls, and not specifically relating to mature fishes.

[ii] A total of 691 fish sizes were used to estimate mean capture weights in the main estimate for 2000-2019, out of a total 805 collected from internet searches, with 114 excluded by the data ranking system (see text).
