# Supplementary material for: Estimating global numbers of fishes caught from the wild annually from 2000 to 2019
Source: Anim Welf. 2024 Feb 8;33:e6. doi: 10.1017/awf.2024.7 (PMC10951671; doi:10.1017/awf.2024.7)
Supplement: Mood and Brooke supplementary material 3 — Mood and Brooke supplementary material [file S0962728624000071sup003.pdf]

Estimated average annual wild-caught finfish numbers (2000-2019)

Rank	Species	Scientific name	FAO finfish capture (tonnes)	Lower estimated numbers (2 significant figures)	Upper estimated numbers (2 significant figures)	Estimate midpoint (2 significant figures)	EMW lower (g)	EMW upper (g)	EMW/ GEMW type
1	Anchoveta(=Peruvian anchovy)	Engraulis ringens	6,650,981	230,000,000,000	670,000,000,000	450,000,000,000	10	29	S1
2	Marine fishes nei	Osteichthyes	9,228,541	140,000,000,000	280,000,000,000	210,000,000,000	32	67	GA
3	Freshwater fishes nei	Osteichthyes	5,409,395	80,000,000,000	160,000,000,000	120,000,000,000	33	68	GA
4	Stolephorus anchovies nei	Stolephorus spp	304,500	43,000,000,000	120,000,000,000	83,000,000,000	3	7	M2
5	European sprat	Sprattus sprattus	587,379	69,000,000,000	69,000,000,000	69,000,000,000	9	9	S1
6	Japanese anchovy	Engraulis japonicus	1,353,641	62,000,000,000	68,000,000,000	65,000,000,000	20	22	S1
7	European pilchard(=Sardine)	Sardina pilchardus	1,164,686	59,000,000,000	59,000,000,000	59,000,000,000	20	20	S1
8	European anchovy	Engraulis encrasicolus	542,058	14,000,000,000	66,000,000,000	40,000,000,000	8	38	S1
9	Sandeels(=Sandlances) nei	Ammodytes spp	385,561	39,000,000,000	39,000,000,000	39,000,000,000	10	10	M1
10	Araucanian herring	Strangomera bentincki	485,446	20,000,000,000	43,000,000,000	31,000,000,000	11	25	S1
11	Capelin	Mallotus villosus	707,935	14,000,000,000	42,000,000,000	28,000,000,000	17	50	S1
12	Scads nei	Decapterus spp	1,174,227	17,000,000,000	28,000,000,000	22,000,000,000	42	70	GG
13	Clupeoids nei	Clupeoidei	425,376	13,000,000,000	28,000,000,000	21,000,000,000	15	32	GO
14	Cyprinids nei	Cyprinidae	555,578	16,000,000,000	25,000,000,000	21,000,000,000	22	35	GF
15	Anchovies, etc. nei	Engraulidae	255,349	10,000,000,000	26,000,000,000	18,000,000,000	10	25	GF
16	Pacific sandlance	Ammodytes personatus	172,261	17,000,000,000	17,000,000,000	17,000,000,000	10	10	S2
17	Silver cyprinid	Rastrineobola argentea	184,649	12,000,000,000	21,000,000,000	16,000,000,000	9	15	S1
18	Sardinellas nei	Sardinella spp	912,577	12,000,000,000	19,000,000,000	16,000,000,000	47	75	GG
19	Southern African anchovy	Engraulis capensis	223,658	12,000,000,000	12,000,000,000	12,000,000,000	19	19	S1
20	Atlantic herring	Clupea harengus	1,987,021	3,300,000,000	20,000,000,000	12,000,000,000	100	600	S1
21	Blue whiting(=Poutassou)	Micromesistius poutassou	1,381,076	4,600,000,000	17,000,000,000	11,000,000,000	80	300	S1
22	Black and Caspian Sea sprat	Clupeonella cultriventris	80,226	8,300,000,000	13,000,000,000	11,000,000,000	6	10	S1
23	Indian oil sardine	Sardinella longiceps	477,681	9,700,000,000	11,000,000,000	10,000,000,000	42	49	S1
24	Silversides(=Sand smelts) nei	Atherinidae	22,455	3,300,000,000	18,000,000,000	10,000,000,000	1	7	GF
25	Croakers, drums nei	Sciaenidae	722,746	5,900,000,000	11,000,000,000	8,500,000,000	65	122	GF
26	Alaska pollock(=Walleye poll.)	Gadus chalcogrammus	3,052,750	3,100,000,000	13,000,000,000	8,300,000,000	227	1,000	S2
27	Cape horse mackerel	Trachurus capensis	335,716	1,200,000,000	15,000,000,000	8,200,000,000	22	291	S3
28	Yellow croaker	Larimichthys polyactis	340,368	5,200,000,000	9,500,000,000	7,400,000,000	36	66	S2
29	Bombay-duck	Harpadon nehereus	205,632	6,400,000,000	8,000,000,000	7,200,000,000	26	32	S1
30	Goldstripe sardinella	Sardinella gibbosa	182,719	4,000,000,000	8,700,000,000	6,400,000,000	21	46	S1
31	Pacific chub mackerel	Scomber japonicus	1,460,494	3,100,000,000	8,600,000,000	5,900,000,000	169	466	S1
32	Largehead hairtail	Trichiurus lepturus	1,281,875	4,800,000,000	6,500,000,000	5,700,000,000	196	265	S1
33	Pacific anchoveta	Cetengraulis mysticetus	147,921	4,200,000,000	6,000,000,000	5,100,000,000	25	35	S3
34	Yellowstripe scad	Selaroides leptolepis	169,550	3,700,000,000	6,300,000,000	5,000,000,000	27	46	S1
35	Pacific saury	Cololabis saira	413,717	2,400,000,000	7,400,000,000	4,900,000,000	56	172	S1
36	Gulf menhaden	Brevoortia patronus	479,255	3,800,000,000	5,100,000,000	4,400,000,000	95	127	S1
37	Jack and horse mackerels nei	Trachurus spp	458,656	1,200,000,000	7,000,000,000	4,100,000,000	66	394	GG
38	Lizardfishes nei	Synodontidae	122,834	3,400,000,000	4,300,000,000	3,900,000,000	29	36	GF
39	Pacific herring	Clupea pallasii	396,346	930,000,000	6,200,000,000	3,600,000,000	64	426	S1
40	Chilean jack mackerel	Trachurus murphyi	1,157,240	1,200,000,000	5,800,000,000	3,500,000,000	200	1,000	S1
41	Indian scad	Decapterus russelli	152,584	2,200,000,000	4,100,000,000	3,100,000,000	37	70	S1
42	Southern African pilchard	Sardinops ocellatus	161,290	1,600,000,000	4,600,000,000	3,100,000,000	35	100	S1
43	Norway pout	Trisopterus esmarkii	61,526	2,200,000,000	3,800,000,000	3,000,000,000	16	28	S1
44	Threadfin breams nei	Nemipterus spp	552,253	2,200,000,000	3,700,000,000	3,000,000,000	149	253	GG
45	California pilchard	Sardinops caeruleus	427,252	2,300,000,000	3,600,000,000	2,900,000,000	120	183	S1
46	Californian anchovy	Engraulis mordax	35,364	2,600,000,000	2,600,000,000	2,600,000,000	14	14	S1
47	Round sardinella	Sardinella aurita	377,925	1,000,000,000	4,100,000,000	2,600,000,000	92	379	S1
48	Carangids nei	Carangidae	239,232	1,300,000,000	3,800,000,000	2,500,000,000	64	190	GF
49	Indian mackerel	Rastrelliger kanagurta	340,033	2,500,000,000	2,500,000,000	2,500,000,000	138	138	S1
50	Short mackerel	Rastrelliger brachysoma	305,092	1,500,000,000	3,100,000,000	2,300,000,000	100	200	S1
51	Atlantic mackerel	Scomber scombrus	865,220	1,900,000,000	2,300,000,000	2,100,000,000	370	454	S1
52	Bigeye scad	Selar crumenophthalmus	177,488	2,000,000,000	2,000,000,000	2,000,000,000	88	88	S1
53	Japanese pilchard	Sardinops melanostictus	343,781	1,900,000,000	1,900,000,000	1,900,000,000	180	180	S1
54	Madeiran sardinella	Sardinella maderensis	159,153	1,300,000,000	2,600,000,000	1,900,000,000	62	126	S1
55	Bonga shad	Ethmalosa fimbriata	260,365	1,900,000,000	1,900,000,000	1,900,000,000	137	137	S1
56	Indian mackerels nei	Rastrelliger spp	254,444	1,600,000,000	2,200,000,000	1,900,000,000	116	163	GG
57	Longnose anchovy	Anchoa nasus	19,195	1,900,000,000	1,900,000,000	1,900,000,000	10	10	S1
58	Gobies nei	Gobiidae	48,322	1,700,000,000	2,000,000,000	1,800,000,000	25	29	GC[1]
59	Pacific thread herring	Opisthonema libertate	205,129	1,800,000,000	1,800,000,000	1,800,000,000	114	114	S1
60	Cunene horse mackerel	Trachurus trecae	67,045	130,000,000	3,200,000,000	1,700,000,000	21	498	S3
61	Ponyfishes(=Slipmouths) nei	Leiognathidae	212,076	930,000,000	2,400,000,000	1,700,000,000	89	227	GO
62	Silver pomfrets nei	Pampus spp	337,015	1,300,000,000	2,000,000,000	1,700,000,000	169	258	GG
63	Goatfishes	Upeneus spp	99,430	630,000,000	2,600,000,000	1,600,000,000	38	157	GF
64	Dagaas (=Kapenta)	Stolothrissa, Limnothrissa spp	61,319	1,400,000,000	1,900,000,000	1,600,000,000	33	44	GF
65	Spinefeet(=Rabbitfishes) nei	Siganus spp	72,590	1,000,000,000	2,100,000,000	1,600,000,000	34	69	GC
66	Chirostoma spp	Chirostoma spp	3,152	470,000,000	2,500,000,000	1,500,000,000	1	7	GF
67	Bigeyes nei	Priacanthus spp	122,556	590,000,000	2,300,000,000	1,500,000,000	52	207	GG
68	Atlantic chub mackerel	Scomber colias	348,174	670,000,000	2,300,000,000	1,500,000,000	155	520	S1
69	Atlantic horse mackerel	Trachurus trachurus	191,165	380,000,000	2,300,000,000	1,300,000,000	83	500	S2
70	Pelagic percomorphs nei	Perciformes	54,334	830,000,000	1,700,000,000	1,300,000,000	31	65	GC
71	Buccaneer anchovy	Encrasicholina punctifer	2,766	1,300,000,000	1,300,000,000	1,300,000,000	2	2	S1
72	Red-eye round herring	Etrumeus teres	78,032	1,200,000,000	1,200,000,000	1,200,000,000	63	63	S1
73	Japanese sandfish	Arctoscopus japonicus	12,974	1,200,000,000	1,200,000,000	1,200,000,000	10	10	GO
74	Demersal percomorphs nei	Perciformes	54,177	800,000,000	1,700,000,000	1,200,000,000	33	67	GC
75	Rainbow sardine	Dussumieria acuta	31,637	1,200,000,000	1,200,000,000	1,200,000,000	26	26	S1
76	Falkland sprat	Sprattus fuegensis	20,026	1,100,000,000	1,300,000,000	1,200,000,000	16	19	S3
77	Japanese jack mackerel	Trachurus japonicus	252,683	1,200,000,000	1,200,000,000	1,200,000,000	217	217	S1
78	Argentine anchovy	Engraulis anchoita	21,248	820,000,000	1,400,000,000	1,100,000,000	15	26	S1
79	Bali sardinella	Sardinella lemuru	100,430	1,000,000,000	1,100,000,000	1,100,000,000	90	100	S1
80	Hairtails, scabbardfishes nei	Trichiuridae	240,440	900,000,000	1,200,000,000	1,100,000,000	198	268	GF
81	Snakeskin gourami	Trichogaster pectoralis	30,942	820,000,000	1,000,000,000	930,000,000	30	38	S1
82	Mediterranean horse mackerel	Trachurus mediterraneus	21,588	860,000,000	1,100,000,000	960,000,000	20	25	S1
83	Lake Malawi sardine	Engraulicypris sardella	47,789	960,000,000	960,000,000	960,000,000	50	50	S1
84	Whitehead's round herring	Etrumeus whiteheadi	49,886	780,000,000	1,100,000,000	940,000,000	45	64	S3
85	Hilsa shad	Tenualosa ilisha	387,372	420,000,000	1,500,000,000	940,000,000	266	915	S1
86	Brazilian sardinella	Sardinella brasiliensis	54,176	900,000,000	900,000,000	900,000,000	60	60	S1
87	Argentines	Argentina spp	34,270	500,000,000	1,300,000,000	900,000,000	26	69	GO[1]
88	Nile tilapia	Oreochromis niloticus	241,528	320,000,000	1,500,000,000	890,000,000	166	750	S2
89	Atlantic menhaden	Brevoortia tyrannus	201,501	500,000,000	1,200,000,000	870,000,000	162	400	S1
90	Skipjack tuna	Katsuwonus pelamis	2,559,551	280,000,000	1,400,000,000	850,000,000	1,800	9,070	S1
91	Silver barb	Barbonymus gonionotus	47,557	840,000,000	840,000,000	840,000,000	56	56	S1
92	Silver croaker	Pennahia argentata	94,989	470,000,000	1,200,000,000	830,000,000	80	200	S2
93	Tilapias nei	Oreochromis (=Tilapia) spp	399,833	800,000,000	800,000,000	800,000,000	500	500	M1
94	Atlantic cod	Gadus morhua	1,039,422	260,000,000	1,300,000,000	780,000,000	800	4,000	S1
95	Sea catfishes nei	Ariidae	375,061	290,000,000	1,300,000,000	770,000,000	300	1,300	GF
96	Boarfish	Capros aper	28,270	590,000,000	950,000,000	770,000,000	30	48	S1
97	Nurse tetra	Brycinus nurse	29,183	740,000,000	740,000,000	740,000,000	39	39	S1
98	Fusiliers nei	Caesionidae	88,568	400,000,000	1,000,000,000	710,000,000	87	221	GO
99	Flyingfishes nei	Exocoetidae	58,610	350,000,000	1,000,000,000	700,000,000	56	169	GO

100	Threadfins, tasselfishes nei	Polynemidae	83,571	640,000,000	700,000,000	670,000,000	120	131	GF
101	Percoids nei	Percoidei	85,597	370,000,000	950,000,000	660,000,000	90	230	GO
102	Big-scale sand smelt	Atherina boyeri	1,129	190,000,000	1,100,000,000	660,000,000	1	6	S2
103	Pouting(=Bib)	Trisopterus luscus	13,477	480,000,000	830,000,000	650,000,000	16	28	GG
104	Argentine hake	Merluccius hubbsi	360,656	550,000,000	700,000,000	630,000,000	518	650	S1
105	Prochilods nei	Prochilodus spp	58,494	610,000,000	620,000,000	610,000,000	95	95	GO
106	Pond smelt	Hypomesus olidus	3,171	170,000,000	1,000,000,000	600,000,000	3	19	S2
107	Seerfishes nei	Scomberomorus spp	455,650	230,000,000	950,000,000	590,000,000	482	1,944	GG
108	Climbing perch	Anabas testudineus	26,223	570,000,000	570,000,000	570,000,000	46	46	S1
109	Cape hakes	Merluccius capensis, M.paradoxus	290,979	420,000,000	760,000,000	590,000,000	383	695	GG
110	Porgies, seabreams nei	Sparidae	214,117	450,000,000	630,000,000	540,000,000	339	477	GF
111	Spotted sardinella	Amblygaster sirm	18,823	360,000,000	710,000,000	530,000,000	26	53	S1
112	Halfbeaks nei	Hemiramphidae	42,699	220,000,000	840,000,000	530,000,000	51	197	GF
113	Tuna-like fishes nei	Scombroidei	252,289	370,000,000	680,000,000	530,000,000	371	682	GO
114	Pacific menhaden	Ethmidium maculatum	19,763	450,000,000	600,000,000	530,000,000	33	44	GF
115	Japanese scad	Decapterus maruadsi	32,546	520,000,000	520,000,000	520,000,000	63	63	S1
116	Goatfishes, red mullets nei	Mullidae	31,293	200,000,000	830,000,000	510,000,000	38	157	GF
117	Snakeheads(=Murrels) nei	Channa spp	101,462	500,000,000	510,000,000	500,000,000	200	205	GG
118	Chilean silverside	Odontesthes regia	1,053	160,000,000	820,000,000	490,000,000	1	7	GF
119	Grunts, sweetlips nei	Haemulidae (=Pomadasyidae)	72,805	320,000,000	640,000,000	480,000,000	113	227	GF
120	Finfishes nei	Osteichthyes	19,794	310,000,000	640,000,000	480,000,000	31	64	GA
121	Torpedo scad	Megalaspis cordyla	103,078	470,000,000	470,000,000	470,000,000	222	222	S1
122	North Pacific hake	Merluccius productus	290,631	280,000,000	640,000,000	460,000,000	454	1,035	S1
123	Jacks, crevalles nei	Caranx spp	201,440	150,000,000	690,000,000	420,000,000	293	1,318	GG
124	Kissing gourami	Helostoma temminckii	13,318	370,000,000	470,000,000	420,000,000	28	36	S1
125	Daggertooth pike conger	Muraenesox cinereus	321,114	110,000,000	640,000,000	370,000,000	500	3,000	S2
126	Filefishes nei	Cantherhines (=Navodon) spp	179,504	360,000,000	380,000,000	370,000,000	468	495	GO
127	Tarek	Chalcalburnus tarichi	11,534	290,000,000	450,000,000	370,000,000	26	40	GF
128	Mouthbrooding cichlids	Haplochromis spp	18,270	370,000,000	370,000,000	370,000,000	50	50	M1
129	Needlefishes nei	Tylosurus spp	18,688	360,000,000	360,000,000	360,000,000	52	52	GF
130	Red mullet	Mullus barbatus	13,445	130,000,000	580,000,000	360,000,000	23	100	S1
131	Beaked redfish	Sebastes mentella	68,668	350,000,000	350,000,000	350,000,000	195	195	S1
132	Butterfishes, pomfrets nei	Stromateidae	67,579	260,000,000	400,000,000	330,000,000	168	258	GF
133	South Pacific hake	Merluccius gayi	108,115	320,000,000	320,000,000	320,000,000	341	341	S1
134	Three spot gourami	Trichogaster trichopterus	9,233	240,000,000	310,000,000	280,000,000	30	38	GG
135	Striped snakehead	Channa striata	62,673	310,000,000	310,000,000	310,000,000	200	205	S1
136	Freshwater siluroids nei	Siluroidei	145,935	300,000,000	310,000,000	300,000,000	477	494	GO
137	Whitemouth croaker	Micropogonias furnieri	90,000	150,000,000	450,000,000	300,000,000	200	600	S2
138	Frigate and bullet tunas	Auxis thazard, A. rochei	304,936	300,000,000	300,000,000	300,000,000	1,024	1,024	GG
139	Okhotsk atka mackerel	Pleurogrammus azonus	148,308	300,000,000	300,000,000	300,000,000	500	500	S1
140	Flatfishes nei	Pleuronectiformes	236,545	180,000,000	410,000,000	300,000,000	582	1,285	GO
141	Emperors(=Scavengers) nei	Lethrinidae	95,258	210,000,000	380,000,000	290,000,000	252	451	GO[1]
142	Diadromous clupeoids nei	Clupeoidei	6,028	190,000,000	400,000,000	290,000,000	15	32	GO
143	Whiting	Merlangius merlangus	48,601	97,000,000	490,000,000	290,000,000	100	500	S1
144	Japanese smelt	Hypomesus nipponensis	1,476	79,000,000	480,000,000	280,000,000	3	19	GG
145	Chacunda gizzard shad	Anodontostoma chacunda	15,914	280,000,000	280,000,000	280,000,000	57	57	S1
146	Royal threadfin	Pentanemus quinquarius	10,940	260,000,000	300,000,000	280,000,000	37	43	S1
147	Mullets nei	Mugilidae	258,341	220,000,000	330,000,000	270,000,000	789	1,183	GF
148	Threadfin and dwarf breams nei	Nemipteridae	53,307	220,000,000	320,000,000	270,000,000	165	245	GF
149	Narrow-barred Spanish mackerel	Scomberomorus commerson	248,419	42,000,000	500,000,000	270,000,000	500	5,900	S1
150	Haddock	Melanogrammus aeglefinus	323,263	180,000,000	360,000,000	270,000,000	900	1,800	S1
151	Yellowfin sole	Limanda aspera	109,748	160,000,000	370,000,000	260,000,000	300	700	S1
152	Freshwater gobies nei	Gobiidae	6,117	240,000,000	270,000,000	260,000,000	23	25	GC[1]
153	Kawakawa	Euthynnus affinis	321,326	160,000,000	340,000,000	250,000,000	943	2,024	S1
154	Bigeye grunt	Brachydeuterus auritus	32,826	160,000,000	330,000,000	250,000,000	100	200	S2
155	Pink(=Humpback) salmon	Oncorhynchus gorbuscha	415,105	180,000,000	300,000,000	240,000,000	1,364	2,273	S1
156	Lake Tanganyika sardine	Limnothrissa miodon	9,335	210,000,000	280,000,000	240,000,000	34	45	GF
157	Chinese gizzard shad	Clupanodon thrissa	9,140	200,000,000	280,000,000	240,000,000	33	45	GF
158	Silver pomfret	Pampus argenteus	48,315	190,000,000	290,000,000	240,000,000	169	258	S1
159	Groundfishes nei	Osteichthyes	9,816	150,000,000	300,000,000	230,000,000	32	67	GA
160	Mudfish	Clarias anguillaris	34,458	220,000,000	220,000,000	220,000,000	154	154	S2
161	Dotted gizzard shad	Konosirus punctatus	8,425	190,000,000	260,000,000	220,000,000	33	44	GF
162	Common pandora	Pagellus erythrinus	12,840	220,000,000	220,000,000	220,000,000	59	59	GG
163	Surmullets(=Red mullets) nei	Mullus spp	12,716	87,000,000	350,000,000	220,000,000	37	147	GG
164	Pelagic fishes nei	Osteichthyes	9,366	140,000,000	290,000,000	210,000,000	33	66	GA
165	Torpedo-shaped catfishes nei	Clarias spp	78,597	210,000,000	210,000,000	210,000,000	372	374	GG
166	Elongate ilisha	Ilisha elongata	84,108	140,000,000	280,000,000	210,000,000	300	600	S2
167	Pacific cod	Gadus macrocephalus	396,536	87,000,000	330,000,000	210,000,000	1,200	4,545	S1
168	Sillago-whitings	Sillaginidae	27,170	120,000,000	300,000,000	210,000,000	90	233	GO
169	West African croakers nei	Pseudotolithus spp	51,016	200,000,000	220,000,000	210,000,000	234	257	GG
170	Orfe(=Ide)	Leuciscus idus	5,737	160,000,000	250,000,000	210,000,000	23	36	GF
171	Nile perch	Lates niloticus	280,455	70,000,000	340,000,000	210,000,000	825	4,000	S1
172	Atlantic redfishes nei	Sebastes spp	69,263	180,000,000	220,000,000	200,000,000	315	376	GG
173	Smelts nei	Osmerus spp, Hypomesus spp	6,702	62,000,000	330,000,000	200,000,000	20	107	GG
174	Atlantic bumper	Chloroscombrus chrysurus	24,135	170,000,000	220,000,000	200,000,000	108	140	S3
175	Bigeye tuna	Thunnus obesus	419,773	12,000,000	380,000,000	200,000,000	1,100	35,980	S1
176	Nilem carp	Osteochilus hasselti	5,112	150,000,000	240,000,000	200,000,000	21	34	GF
177	Mystacoleucus padangensis	Mystacoleucus padangensis	4,708	150,000,000	240,000,000	190,000,000	20	31	GF
178	False trevally	Lactarius lactarius	25,539	190,000,000	190,000,000	190,000,000	132	132	S1
179	Silver-stripe round herring	Spratelloides gracilis	271	150,000,000	230,000,000	190,000,000	1	2	S1
180	Atlantic thread herring	Opisthonema oglinum	14,126	160,000,000	220,000,000	190,000,000	65	91	S2
181	Southern blue whiting	Micromesistius australis	88,589	150,000,000	220,000,000	180,000,000	400	600	S1
182	South American pilchard	Sardinops sagax	27,152	170,000,000	300,000,000	240,000,000	90	159	GG
183	Surgeonfishes nei	Acanthuridae	7,820	120,000,000	240,000,000	180,000,000	33	67	GC
184	European hake	Merluccius merluccius	97,603	70,000,000	560,000,000	320,000,000	174	1,400	S1
185	Lake Tanganyika sprat	Stolothrissa tanganicae	6,533	150,000,000	200,000,000	170,000,000	33	45	GF
186	South American silver croaker	Plagioscion squamosissimus	14,542	120,000,000	220,000,000	170,000,000	65	123	GF
187	European smelt	Osmerus eperlanus	4,830	42,000,000	290,000,000	170,000,000	16	116	S1
188	Kissing prochilodus	Semaprochilodus insignis	13,842	160,000,000	170,000,000	170,000,000	84	84	GO
189	Gudgeons, sleepers nei	Eleotridae	4,048	150,000,000	170,000,000	160,000,000	23	26	GC[1]
190	Argentine	Argentina sphyraena	3,028	91,000,000	230,000,000	160,000,000	13	33	S2
191	Picarels nei	Spicara spp	6,108	140,000,000	170,000,000	160,000,000	35	44	GG
192	Cichlids nei	Cichlidae	50,187	110,000,000	190,000,000	150,000,000	258	455	GF
193	Shortfin scad	Decapterus macrosoma	15,538	150,000,000	150,000,000	150,000,000	105	105	S1
194	Red pandora	Pagellus bellottii	8,861	140,000,000	140,000,000	140,000,000	61	61	GG
195	Citharinus nei	Citharinus spp	11,383	140,000,000	140,000,000	140,000,000	79	79	GO
196	Skinnycheek lanternfish	Benthosema pterotum	6,813	96,000,000	190,000,000	140,000,000	35	71	GC
197	Hoven's carp	Leptobarbus hoeveni	4,031	110,000,000	170,000,000	140,000,000	23	37	GF
198	Polar cod	Boreogadus saida	14,852	130,000,000	160,000,000	140,000,000	95	119	S3
199	Salmonoids nei	Salmonoidei	10,430	71,000,000	210,000,000	140,000,000	50	147	GO
200	Rudd	Scardinius erythrophthalmus	3,455	110,000,000	170,000,000	140,000,000	20	32	GF
201	Red-toothed triggerfish	Odonus niger	17,292	120,000,000	150,000,000	140,000,000	115	140	S1
202	Mote sculpin	Normanichthys crockeri	64,326	130,000,000	150,000,000	140,000,000	441	505	GO
203	Bogue	Boops boops	33,726	100,000,000	170,000,000	130,000,000	200	333	S1

204	Moonfish	Mene maculata	17,086	76,000,000	190,000,000	130,000,000	88	225	GO
205	Scorpionfishes, redfishes nei	Scorpaenidae	46,046	120,000,000	140,000,000	130,000,000	322	384	GF
206	Snappers nei	Lutjanus spp	158,270	70,000,000	190,000,000	130,000,000	824	2,249	GG
207	Saithe(=Pollock)	Pollachius virens	370,537	82,000,000	170,000,000	130,000,000	2,200	4,500	S1
208	Black pomfret	Parastromateus niger	71,743	100,000,000	140,000,000	120,000,000	500	700	S2
209	European plaice	Pleuronectes platessa	94,431	86,000,000	160,000,000	120,000,000	598	1,100	S1
210	Saffron cod	Eleginus gracilis	32,360	81,000,000	160,000,000	120,000,000	200	400	S2
211	Eulachon	Thaleichthys pacificus	2,933	58,000,000	180,000,000	120,000,000	16	50	GF
212	Flathead grey mullet	Mugil cephalus	128,815	95,000,000	140,000,000	120,000,000	907	1,361	S1
213	Lesser African threadfin	Galeoides decadactylus	20,363	110,000,000	120,000,000	120,000,000	168	177	S1
214	Mackerels nei	Scombridae	65,242	76,000,000	160,000,000	120,000,000	409	853	GF
215	Silver hake	Merluccius bilinearis	18,135	83,000,000	150,000,000	120,000,000	122	218	S1
216	King weakfish	Macrodon ancylodon	9,800	80,000,000	150,000,000	120,000,000	65	122	GF
217	White bream	Blicca bjoerkna	2,924	90,000,000	140,000,000	120,000,000	21	33	GF
218	Japanese Spanish mackerel	Scomberomorus niphonius	52,717	53,000,000	180,000,000	110,000,000	300	1,000	S2
219	Bobo croaker	Pseudotolithus elongatus	23,970	110,000,000	110,000,000	110,000,000	216	216	S3
220	Patagonian grenadier	Macruronus magellanicus	164,549	110,000,000	110,000,000	110,000,000	1,500	1,500	S1
221	Blue grenadier	Macruronus novaezelandiae	158,786	110,000,000	110,000,000	110,000,000	1,500	1,500	S1
222	Argentinian silverside	Odontesthes bonariensis	223	34,000,000	180,000,000	110,000,000	1	7	GF
223	Chocolate hind	Cephalopholis boenak	48,278	48,000,000	160,000,000	100,000,000	300	1,000	S2
224	Surmullet	Mullus surmuletus	12,858	49,000,000	160,000,000	100,000,000	81	260	S2
225	Vendace	Coregonus albula	6,244	52,000,000	160,000,000	100,000,000	40	120	S1
226	Deep-sea smelt	Glossanodon semifasciatus	4,030	55,000,000	140,000,000	99,000,000	28	74	GO[1]
227	Hoplias aimara	Hoplias aimara	8,261	98,000,000	99,000,000	98,000,000	84	84	GO
228	Grass-eaters nei	Distichodus spp	6,605	97,000,000	97,000,000	97,000,000	68	68	GO
229	Yellowfin tuna	Thunnus albacares	1,340,308	40,000,000	150,000,000	95,000,000	9,000	33,430	S1
230	Frigate tuna	Auxis thazard	96,298	93,000,000	93,000,000	93,000,000	1,040	1,040	S1
231	Ayu sweetfish	Plecoglossus altivelis	4,709	46,000,000	140,000,000	92,000,000	34	102	GO
232	Giant African threadfin	Polydactylus quadrifilis	24,394	91,000,000	91,000,000	91,000,000	269	269	S1
233	Tinfoil barb	Barbonymus schwanenfeldii	5,078	90,000,000	90,000,000	90,000,000	56	56	GG
234	Silvery lightfish	Maurolicus muelleri	3,824	59,000,000	120,000,000	90,000,000	32	64	GC
235	Pacific cornetfish	Fistularia corneta	4,233	59,000,000	120,000,000	89,000,000	36	71	GC
236	Atka mackerel	Pleurogrammus monopterygius	52,957	79,000,000	93,000,000	86,000,000	570	670	S1
237	Netted prochilod	Prochilodus reticulatus	9,349	85,000,000	85,000,000	85,000,000	110	111	GO
238	Dentex nei	Dentex spp	24,979	43,000,000	120,000,000	83,000,000	203	587	GG
239	Japanese seabass	Lateolabrax japonicus	10,324	45,000,000	120,000,000	80,000,000	89	230	GO
240	Poor cod	Trisopterus minutus	1,639	58,000,000	100,000,000	80,000,000	16	28	GG
241	Pandoras nei	Pagellus spp	4,791	79,000,000	79,000,000	79,000,000	61	61	GG
242	Elephantsnout fishes nei	Mormyridae	32,114	76,000,000	81,000,000	79,000,000	395	424	GO
243	Barracudas nei	Sphyraena spp	118,005	14,000,000	140,000,000	75,000,000	870	8,202	GG
244	Large yellow croaker	Larimichthys croceus	74,976	75,000,000	75,000,000	75,000,000	1,000	1,000	S1
245	Silver rasbora	Rasbora argyrotaenia	1,886	57,000,000	91,000,000	74,000,000	21	33	GF
246	Fourfinger threadfin	Eleutheronema tetradactylum	9,068	70,000,000	77,000,000	74,000,000	118	129	GF
247	Indo-Pacific king mackerel	Scomberomorus guttatus	49,330	70,000,000	77,000,000	74,000,000	640	702	S1
248	Oreo dories nei	Oreosomatidae	5,879	55,000,000	89,000,000	72,000,000	66	107	GO
249	African lungfishes	Protopterus spp	15,593	46,000,000	97,000,000	72,000,000	161	337	GA[1]
250	So-iny (redlip) mullet	Liza haematocheilus	115,542	46,000,000	95,000,000	70,000,000	1,217	2,535	S1
251	Smooth oreo dory	Pseudocyttus maculatus	6,117	53,000,000	85,000,000	69,000,000	72	116	GO
252	Chum(=Keta=Dog) salmon	Oncorhynchus keta	315,307	39,000,000	99,000,000	69,000,000	3,182	8,182	S1
253	Common sole	Solea solea	36,824	11,000,000	120,000,000	67,000,000	300	3,500	S1
254	African bonytongue	Heterotis niloticus	18,264	66,000,000	66,000,000	66,000,000	275	277	S1
255	Whitefishes nei	Coregonus spp	10,293	36,000,000	96,000,000	66,000,000	107	286	GG
256	Mojarras(=Silver-biddies) nei	Gerres spp	8,424	37,000,000	94,000,000	66,000,000	89	229	GO
257	Batfishes	Platax spp	2,840	43,000,000	88,000,000	65,000,000	32	67	GC
258	Corvina drum	Cilus gilberti	5,452	45,000,000	85,000,000	65,000,000	64	121	GF
259	West African goatfish	Pseudupeneus prayensis	5,558	19,000,000	110,000,000	65,000,000	50	300	S2
260	Sichel	Pelecus cultratus	1,785	49,000,000	77,000,000	63,000,000	23	36	GF
261	Scats	Scatophagus spp	2,679	40,000,000	84,000,000	62,000,000	32	66	GC
262	Pompanos nei	Trachinotus spp	5,876	32,000,000	92,000,000	62,000,000	64	184	GF
263	Atlantic silverside	Menidia menidia	385	40,000,000	83,000,000	62,000,000	5	10	S3
264	Golden redfish	Sebastes marinus	55,674	28,000,000	93,000,000	60,000,000	600	2,000	S1
265	Wolf-herrings nei	Chirocentrus spp	50,945	44,000,000	76,000,000	60,000,000	670	1,153	GG
266	Roaches nei	Rutilus spp	18,026	60,000,000	60,000,000	60,000,000	300	300	GG
267	Milkfish	Chanos chanos	9,003	30,000,000	90,000,000	60,000,000	100	300	S2
268	Klunzinger's mullet	Liza klunzingeri	4,256	60,000,000	60,000,000	60,000,000	71	71	S1
269	Pacific ocean perch	Sebastes alutus	40,981	51,000,000	68,000,000	60,000,000	600	800	S1
270	Puntioplites waandersi	Puntioplites waandersi	1,464	46,000,000	72,000,000	59,000,000	20	32	GF
271	Brazilian codling	Urophycis brasiliensis	6,227	52,000,000	66,000,000	59,000,000	94	121	S3
272	Rhinofishes nei	Labeo spp	1,756	46,000,000	72,000,000	59,000,000	25	39	GF
273	Snappers, jobfishes nei	Lutjanidae	75,344	46,000,000	71,000,000	59,000,000	1,059	1,641	GF
274	Greater lizardfish	Saurida tumbil	25,733	58,000,000	58,000,000	58,000,000	441	441	S1
275	Atlantic saury	Scomberesox saurus	4,280	39,000,000	78,000,000	58,000,000	55	110	S1
276	Axillary seabream	Pagellus acarne	1,906	57,000,000	57,000,000	57,000,000	33	33	S1
277	Terapon perches nei	Terapon spp	6,791	32,000,000	80,000,000	56,000,000	85	213	GO
278	Sharks, rays, skates, etc. nei	Elasmobranchii	271,647	29,000,000	81,000,000	55,000,000	3,340	9,376	GC
279	Sockeye(=Red) salmon	Oncorhynchus nerka	152,937	42,000,000	66,000,000	54,000,000	2,300	3,600	S1
280	Curimata cyprinoides	Curimata cyprinoides	4,471	52,000,000	52,000,000	52,000,000	85	86	GO
281	Schizodon fasciatus	Schizodon fasciatus	2,952	52,000,000	52,000,000	52,000,000	57	57	GO
282	Goldfish	Carassius auratus	8,076	50,000,000	50,000,000	50,000,000	162	162	S2
283	Buffalofishes nei	Ictiobus spp	1,487	38,000,000	60,000,000	49,000,000	25	39	GO
284	Blue jack mackerel	Trachurus picturatus	5,078	13,000,000	84,000,000	49,000,000	60	378	GG
285	Groupers, seabasses nei	Serranidae	62,478	22,000,000	75,000,000	48,000,000	837	2,815	GF
286	Indonesian snakehead	Channa micropeltes	9,740	48,000,000	49,000,000	48,000,000	200	205	GG
287	Thynnichthys vaillanti	Thynnichthys vaillanti	1,188	37,000,000	59,000,000	48,000,000	20	32	GF
288	Gadiformes nei	Gadiformes	13,923	23,000,000	73,000,000	48,000,000	191	616	GO
289	Moras nei	Moridae	20,478	24,000,000	71,000,000	47,000,000	289	871	GF
290	Atlantic bonito	Sarda sarda	34,006	47,000,000	47,000,000	47,000,000	729	729	S1
291	Blue mackerel	Scomber australasicus	14,126	20,000,000	71,000,000	45,000,000	200	700	S1
292	Rock sole	Lepidopsetta bilineata	40,915	45,000,000	45,000,000	45,000,000	907	907	S1
293	American gizzard shad	Dorosoma cepedianum	1,542	3,000,000	85,000,000	44,000,000	18	520	S2
294	Hagfishes nei	Myxinidae	1,909	28,000,000	58,000,000	43,000,000	33	67	GA
295	West African ilisha	Ilisha africana	17,333	29,000,000	58,000,000	43,000,000	300	600	GG
296	Common carp	Cyprinus carpio	93,168	36,000,000	49,000,000	42,000,000	1,890	2,611	S1
297	Indian pellona	Pellona ditchela	16,768	28,000,000	56,000,000	42,000,000	300	600	GF
298	Grenadiers, rattails nei	Macrouridae	22,256	30,000,000	53,000,000	42,000,000	420	730	GF
299	Greenland halibut	Reinhardtius hippoglossoides	117,092	4,700,000	78,000,000	41,000,000	1,500	25,000	S1
300	Upsidedown catfishes	Synodontis spp	19,891	40,000,000	41,000,000	40,000,000	486	503	GO
301	Queenfishes	Scomberoides spp	30,698	19,000,000	61,000,000	40,000,000	499	1,650	GG
302	Mi-iuy (brown) croaker	Miichthys miiuy	39,980	40,000,000	40,000,000	40,000,000	1,000	1,000	S2
303	Giant gourami	Osphronemus goramy	2,131	34,000,000	41,000,000	38,000,000	52	62	GO
304	Tonguefishes	Cynoglossidae	31,697	25,000,000	54,000,000	40,000,000	583	1,285	GO
305	Small-spotted catshark	Scyliorhinus canicula	6,695	39,000,000	39,000,000	39,000,000	172	172	GF
306	White trevally	Pseudocaranx dentex	3,674	19,000,000	57,000,000	38,000,000	64	192	GF
307	Cape bonnetmouth	Emmelichthys nitidus	3,280	11,000,000	66,000,000	38,000,000	50	300	S2

308	Groupers nei	Epinephelus spp	137,783	15,000,000	61,000,000	38,000,000	2,268	9,072	M2
309	Ponyfishes(=Slipmouths)	Leiognathus spp	4,654	21,000,000	54,000,000	38,000,000	86	219	GO
310	Kafue pike	Hepsetus odoe	2,745	37,000,000	37,000,000	37,000,000	73	74	GO
311	Indian threadfin	Leptomelanosoma indicum	4,159	35,000,000	39,000,000	37,000,000	106	117	GF
312	Rays, stingrays, mantas nei	Rajiformes	140,659	25,000,000	48,000,000	37,000,000	2,922	5,624	GO
313	Prochilodus mariae	Prochilodus mariae	1,543	37,000,000	37,000,000	37,000,000	42	42	GO
314	Javelin grunter	Pomadasys kaakan	5,418	35,000,000	38,000,000	37,000,000	144	153	GF[1]
315	Japanese sardinella	Sardinella zunasi	2,022	12,000,000	61,000,000	36,000,000	33	167	S2
316	Three-spined stickleback	Gasterosteus aculeatus	1,508	23,000,000	49,000,000	36,000,000	31	64	GC
317	Lanternfishes nei	Myctophidae	1,473	23,000,000	48,000,000	36,000,000	31	63	GC
318	Kutum	Rutilus frisii	10,645	35,000,000	35,000,000	35,000,000	300	300	GG
319	Donkey croaker	Pennahia anea	3,500	35,000,000	35,000,000	35,000,000	99	99	S1
320	Scaled sardines	Harengula spp	1,326	30,000,000	41,000,000	35,000,000	33	44	GF
321	Parrotfishes nei	Scaridae	4,470	20,000,000	50,000,000	35,000,000	89	225	GO
322	Roach	Rutilus rutilus	10,513	35,000,000	35,000,000	35,000,000	300	300	S1
323	Garfish	Belone belone	1,738	35,000,000	35,000,000	35,000,000	50	50	S1
324	Common dab	Limanda limanda	11,767	35,000,000	35,000,000	35,000,000	340	340	S1
325	Suckers nei	Catostomidae	852	26,000,000	42,000,000	34,000,000	20	32	GO
326	Black oreo	Allocyttus niger	2,716	26,000,000	42,000,000	34,000,000	65	105	GO
327	Blue runner	Caranx crysos	6,737	7,400,000	60,000,000	34,000,000	113	907	S2
328	Shortjaw leatherjacket	Oligoplites refulgens	2,783	16,000,000	50,000,000	33,000,000	56	173	GF
329	Orangespotted trevally	Carangoides bajad	3,040	16,000,000	49,000,000	33,000,000	62	187	GF
330	Gurnards, searobins nei	Triglidae	13,261	29,000,000	36,000,000	33,000,000	365	454	GF
331	Mojarras, etc. nei	Gerreidae	4,165	18,000,000	47,000,000	33,000,000	88	230	GO
332	Lake Malawi utaka	Copadichromis virginalis	10,243	24,000,000	41,000,000	32,000,000	252	422	GF
333	Barramundi(=Giant seaperch)	Lates calcarifer	86,585	6,200,000	58,000,000	32,000,000	1,500	14,000	S1
334	Pangasius djambal	Pangasius djambal	15,933	31,000,000	32,000,000	32,000,000	493	510	GO
335	Blue butterfish	Stromateus fiatola	6,456	25,000,000	38,000,000	32,000,000	168	258	GF
336	Japanese flyingfish	Cypselurus agoo	2,637	16,000,000	47,000,000	31,000,000	56	169	GO
337	Triggerfishes, durgons nei	Balistidae	11,139	30,000,000	32,000,000	31,000,000	347	373	GF
338	Albacore	Thunnus alalunga	231,278	11,000,000	51,000,000	31,000,000	4,536	21,364	S1
339	Japanese threadfin bream	Nemipterus japonicus	6,038	25,000,000	35,000,000	30,000,000	172	242	S1
340	Tadpole codling	Salilota australis	8,579	14,000,000	43,000,000	29,000,000	200	600	S2
341	Morwongs	Nemadactylus spp	3,406	16,000,000	41,000,000	28,000,000	84	213	GO
342	Monocle breams	Scolopsis spp	5,422	22,000,000	35,000,000	28,000,000	157	249	GF
343	Korean sandlance	Hypoptychus dybowskii	1,172	18,000,000	38,000,000	28,000,000	31	65	GC
344	Sompat grunt	Pomadasys jubelini	4,165	27,000,000	29,000,000	28,000,000	146	152	GF[1]
345	Pacific bumper	Chloroscombrus orqueta	3,418	24,000,000	32,000,000	28,000,000	108	140	GG
346	Arrowtooth flounder	Atheresthes stomias	32,305	25,000,000	30,000,000	28,000,000	1,093	1,268	S3
347	Mozambique tilapia	Oreochromis mossambicus	16,995	27,000,000	27,000,000	27,000,000	620	620	S1
348	Needlefishes, etc. nei	Belonidae	1,415	27,000,000	27,000,000	27,000,000	52	52	GF
349	Laulao catfish	Brachyplatystoma vaillanti	23,180	26,000,000	29,000,000	27,000,000	800	905	S2
350	Witch flounder	Glyptocephalus cynoglossus	12,280	12,000,000	41,000,000	27,000,000	300	1,000	S1
351	Puffers nei	Tetraodontidae	11,994	26,000,000	27,000,000	27,000,000	437	468	GO
352	Streaked prochilod	Prochilodus lineatus	1,099	26,000,000	26,000,000	26,000,000	42	42	GO
353	Roundnose grenadier	Coryphaenoides rupestris	13,903	19,000,000	33,000,000	26,000,000	420	730	S2
354	American yellow perch	Perca flavescens	4,642	10,000,000	41,000,000	25,000,000	114	454	S1
355	Giant trevally	Caranx ignobilis	12,070	9,200,000	42,000,000	25,000,000	289	1,318	GG
356	European flounder	Platichthys flesus	19,923	10,000,000	40,000,000	25,000,000	500	2,000	S1
357	Red bigeye	Priacanthus macracanthus	1,977	9,900,000	40,000,000	25,000,000	50	200	S2
358	Pacific sierra	Scomberomorus sierra	11,069	25,000,000	25,000,000	25,000,000	450	450	S1
359	Spangled emperor	Lethrinus nebulosus	10,108	20,000,000	29,000,000	24,000,000	349	507	GO[1]
360	Pike-congers nei	Muraenesox spp	20,602	6,900,000	41,000,000	24,000,000	500	3,000	GG
361	Black scabbardfish	Aphanopus carbo	9,938	21,000,000	27,000,000	24,000,000	367	474	S3
362	Cabinza grunt	Isacia conceptionis	3,554	16,000,000	31,000,000	24,000,000	113	226	GF
363	European whitefish	Coregonus lavaretus	3,544	13,000,000	34,000,000	23,000,000	104	280	GG
364	Tarakihi	Nemadactylus macropterus	3,186	13,000,000	34,000,000	23,000,000	95	249	GO
365	Characins nei	Characidae	83,961	20,000,000	27,000,000	23,000,000	3,160	4,300	GF
366	Semah mahseer	Tor douronensis	599	18,000,000	28,000,000	23,000,000	21	34	GF
367	Peruvian banded croaker	Paralonchurus peruanus	1,935	16,000,000	30,000,000	23,000,000	65	122	GF
368	Naked catfishes	Bagrus spp	18,928	23,000,000	23,000,000	23,000,000	830	830	GF
369	Bleak	Alburnus alburnus	522	10,000,000	35,000,000	23,000,000	15	50	S1
370	Golden trevally	Gnathanodon speciosus	2,127	11,000,000	34,000,000	23,000,000	63	190	GF
371	Asian redtail catfish	Hemibagrus nemurus	18,603	22,000,000	22,000,000	22,000,000	830	830	GF
372	Signal barb	Labiobarbus festivus	555	17,000,000	27,000,000	22,000,000	20	32	GF
373	Ballyhoo halfbeak	Hemiramphus brasiliensis	1,778	8,900,000	36,000,000	22,000,000	50	200	S2
374	European perch	Perca fluviatilis	26,410	22,000,000	22,000,000	22,000,000	1,200	1,200	S1
375	Yellow striped flounder	Pseudopleuronectes herzenst.	17,315	19,000,000	25,000,000	22,000,000	700	900	GG
376	Hampala barb	Hampala macrolepidota	549	17,000,000	27,000,000	22,000,000	20	32	GF
377	Bagrid catfish	Chrysichthys nigrodigitatus	17,815	21,000,000	21,000,000	21,000,000	830	830	S1
378	Talang queenfish	Scomberoides commersonnianus	16,429	10,000,000	33,000,000	21,000,000	499	1,650	S1
379	Spot croaker	Leiostomus xanthurus	1,811	15,000,000	28,000,000	21,000,000	65	122	GF
380	Blackbelly rosefish	Helicolenus dactylopterus	5,846	21,000,000	21,000,000	21,000,000	275	275	S2
381	Snoek	Thyrsites atun	42,171	14,000,000	28,000,000	21,000,000	1,500	3,000	S1
382	Argentine croaker	Umbrina canosai	14,949	20,000,000	22,000,000	21,000,000	686	739	S3
383	Yellowtail flounder	Limanda ferruginea	11,269	19,000,000	23,000,000	21,000,000	500	600	S1
384	Southern rays bream	Brama australis	17,669	12,000,000	29,000,000	21,000,000	600	1,500	GG
385	Lemon sole	Microstomus kitt	10,575	16,000,000	23,000,000	20,000,000	450	650	S2
386	Atlantic croaker	Micropogonias undulatus	7,213	7,900,000	32,000,000	20,000,000	227	909	S1
387	Common dolphinfish	Coryphaena hippurus	96,940	20,000,000	20,000,000	20,000,000	4,940	4,940	S1
388	Alewife	Alosa pseudoharengus	4,844	18,000,000	21,000,000	20,000,000	227	273	S1
389	Silver grunt	Pomadasys argenteus	2,877	19,000,000	20,000,000	19,000,000	143	154	GF[1]
390	Indian halibut	Psettodes erumei	22,988	19,000,000	19,000,000	19,000,000	1,200	1,200	S1
391	Hector's lanternfish	Lampanyctodes hectoris	824	12,000,000	26,000,000	19,000,000	32	67	GC
392	Guinean striped mojarra	Gerres nigri	2,343	11,000,000	26,000,000	18,000,000	89	223	GO
393	Senegalese hake	Merluccius senegalensis	9,670	14,000,000	26,000,000	20,000,000	378	679	GG
394	Leerfish	Lichia amia	1,744	9,200,000	27,000,000	18,000,000	63	190	GF
395	Little tunny(=Atl.black skipj)	Euthynnus alletteratus	21,746	18,000,000	18,000,000	18,000,000	1,196	1,196	S1
396	Crucian carp	Carassius carassius	11,809	18,000,000	18,000,000	18,000,000	661	661	S1
397	Wrasses, hogfishes, etc. nei	Labridae	17,496	6,500,000	29,000,000	18,000,000	602	2,698	GF
398	Rainbow smelt	Osmerus mordax	1,487	17,000,000	17,000,000	17,000,000	85	85	S1
399	Rainbow runner	Elagatis bipinnulata	17,369	17,000,000	17,000,000	17,000,000	1,000	1,000	S1
400	Blotched picarel	Spicara maena	676	15,000,000	19,000,000	17,000,000	35	44	S1
401	Boe drum	Pteroscion peli	1,777	16,000,000	18,000,000	17,000,000	98	114	S3
402	Tilefishes nei	Branchiostegidae	46,956	9,600,000	24,000,000	17,000,000	1,986	4,878	GF
403	Grey gurnard	Eutrigla gurnardus	3,710	16,000,000	16,000,000	16,000,000	225	225	S1
404	Northern pike	Esox lucius	26,971	5,400,000	27,000,000	16,000,000	1,000	5,000	S1
405	Silver seabream	Pagrus auratus	26,878	16,000,000	16,000,000	16,000,000	1,685	1,685	S1
406	Pink ear emperor	Lethrinus lentjan	5,960	12,000,000	19,000,000	16,000,000	310	487	GO[1]
407	Amberjacks nei	Seriola spp	104,017	11,000,000	20,000,000	15,000,000	5,177	9,635	GG
408	King soldier bream	Argyrops spinifer	6,173	13,000,000	18,000,000	15,000,000	346	489	GF
409	Beardless barb	Cyclocheilichthys apogon	385	12,000,000	18,000,000	15,000,000	21	33	GF
410	Asian swamp eel	Monopterus albus	695	9,800,000	20,000,000	15,000,000	35	71	GC
411	Barbodes balleroides	Barbodes balleroides	362	11,000,000	18,000,000	15,000,000	20	32	GF

412	Dover sole	Microstomus pacificus	7,731	12,000,000	17,000,000	15,000,000	450	650	GG
413	Australian salmon	Arripis trutta	5,469	1,600,000	27,000,000	14,000,000	200	3,500	S1
414	African sicklefish	Drepane africana	5,963	9,900,000	19,000,000	14,000,000	315	602	S2
415	Castaneta	Cheilodactylus bergi	1,877	8,200,000	21,000,000	14,000,000	91	230	GO
416	Brazilian flathead	Percophis brasiliensis	8,217	8,200,000	21,000,000	14,000,000	400	1,000	S2
417	Trahira	Hoplias malabaricus	618	14,000,000	14,000,000	14,000,000	44	44	GO
418	Flathead sole	Hippoglossoides elassodon	15,586	11,000,000	17,000,000	14,000,000	909	1,400	GG
419	Soles nei	Soleidae	7,802	2,200,000	26,000,000	14,000,000	300	3,500	GF
420	Irish mojarra	Diapterus auratus	1,795	8,000,000	20,000,000	14,000,000	89	224	GO
421	Freshwater bream	Abramis brama	50,689	14,000,000	14,000,000	14,000,000	3,600	3,600	S1
422	Caspian shads	Caspialosa spp	522	12,000,000	16,000,000	14,000,000	33	44	GF
423	Lorna drum	Sciaena deliciosa	5,079	12,000,000	15,000,000	14,000,000	335	424	GG
424	Seven khramulya	Capoeta capoeta	331	11,000,000	17,000,000	14,000,000	20	32	GF
425	Leporellus vittatus	Leporellus vittatus	768	13,000,000	13,000,000	13,000,000	57	57	GO
426	Longtail tuna	Thunnus tonggol	230,278	12,000,000	15,000,000	13,000,000	15,000	20,000	S1
427	Weakfishes nei	Cynoscion spp	28,888	12,000,000	14,000,000	13,000,000	2,005	2,381	GG
428	Shads nei	Alosa spp	3,554	12,000,000	14,000,000	13,000,000	246	299	GG
429	Leopard coralgrouper	Plectropomus leopardus	17,747	6,100,000	20,000,000	13,000,000	883	2,923	GF
430	Glassfishes	Ambassidae	1,675	7,300,000	19,000,000	13,000,000	89	229	GO
431	Bullet tuna	Auxis rochei	11,431	13,000,000	13,000,000	13,000,000	876	876	S1
432	Red porgy	Pagrus pagrus	10,234	10,000,000	16,000,000	13,000,000	660	1,000	S1
433	Golden threadfin bream	Nemipterus virgatus	1,925	6,400,000	19,000,000	13,000,000	100	300	S2
434	Greater forkbeard	Phycis blennoides	3,491	5,800,000	20,000,000	13,000,000	177	600	GF
435	Shallow-water Cape hake	Merluccius capensis	4,872	6,400,000	19,000,000	13,000,000	255	767	S1
436	Picked dogfish	Squalus acanthias	18,428	3,200,000	22,000,000	13,000,000	835	5,781	S2
437	Parona leatherjacket	Parona signata	1,277	6,200,000	19,000,000	13,000,000	68	205	GF
438	Clown loach	Chromobotia macracanthus	327	9,600,000	15,000,000	13,000,000	21	34	GO
439	Sargo breams nei	Diplodus spp	5,046	10,000,000	15,000,000	12,000,000	346	487	GF
440	Blackthroat seaperch	Doederleinia berycoides	1,539	7,300,000	18,000,000	12,000,000	87	212	GO
441	Black catfishes nei	Chrysichthys spp	10,166	12,000,000	12,000,000	12,000,000	830	830	GG
442	Long pectoral-fin minnow	Macrochirichthys macrochirus	312	9,500,000	15,000,000	12,000,000	21	33	GF
443	Yellowtail scad	Atule mate	1,139	6,100,000	18,000,000	12,000,000	62	186	GF
444	Atlantic searobins	Prionotus spp	5,179	10,000,000	13,000,000	12,000,000	391	512	GF
445	False scad	Caranx rhonchus	5,882	7,400,000	16,000,000	12,000,000	370	800	S2
446	Red gurnard	Aspitrigla cuculus	5,014	10,000,000	13,000,000	12,000,000	382	495	GF
447	Lumpfish(=Lumpsucker)	Cyclopterus lumpus	15,023	5,200,000	18,000,000	12,000,000	836	2,892	S2
448	Orange roughy	Hoplostethus atlanticus	16,210	12,000,000	12,000,000	12,000,000	1,400	1,400	S1
449	Southern hake	Merluccius australis	37,810	4,200,000	19,000,000	12,000,000	2,000	9,000	S1
450	Flatheads nei	Platycephalidae	4,422	12,000,000	12,000,000	12,000,000	384	384	GF
451	Eastern Pacific bonito	Sarda chiliensis	36,864	6,800,000	16,000,000	12,000,000	2,268	5,443	S1
452	Spadefishes nei	Ephippidae	500	7,500,000	15,000,000	11,000,000	33	67	GC
453	Australian pilchard	Sardinops neopilchardus	685	9,300,000	13,000,000	11,000,000	51	74	S1
454	Grass carp(=White amur)	Ctenopharyngodon idellus	25,654	3,800,000	19,000,000	11,000,000	1,361	6,804	S1
455	Aba	Gymnarchus niloticus	12,284	8,600,000	14,000,000	11,000,000	902	1,430	S1
456	Japanese icefish	Salangichthys microdon	644	5,600,000	17,000,000	11,000,000	39	116	GO
457	Dorab wolf-herring	Chirocentrus dorab	9,639	11,000,000	11,000,000	11,000,000	876	876	S1
458	Large-eye dentex	Dentex macrophthalmus	3,723	5,900,000	16,000,000	11,000,000	232	627	GG
459	Bluefish	Pomatomus saltatrix	20,600	7,600,000	14,000,000	11,000,000	1,429	2,724	S1
460	Cassava croaker	Pseudotolithus senegalensis	5,073	5,000,000	17,000,000	11,000,000	300	1,005	S3
461	Gizzard shad nei	Dorosoma spp	383	740,000	21,000,000	11,000,000	18	520	GG
462	Rubberlip grunt	Plectorhinchus mediterraneus	5,783	7,200,000	14,000,000	11,000,000	400	800	S2
463	African moonfish	Selene dorsalis	2,901	7,300,000	14,000,000	11,000,000	206	397	S2
464	Silver sillago	Sillago sihama	1,262	6,000,000	15,000,000	11,000,000	83	209	GO
465	Mangrove red snapper	Lutjanus argentimaculatus	12,451	5,500,000	15,000,000	10,000,000	814	2,268	GG
466	Bigmouth sleeper	Gobiomorus dormitor	335	9,000,000	12,000,000	10,000,000	29	37	GC[1]
467	Scomber mackerels nei	Scomber spp	2,869	6,100,000	14,000,000	10,000,000	199	470	GG
468	Gilded catfish	Zungaro zungaro	8,667	9,600,000	11,000,000	10,000,000	800	905	GF
469	Great barracuda	Sphyraena barracuda	16,753	1,800,000	18,000,000	10,000,000	907	9,072	S1
470	Atlantic pomfret	Brama brama	8,699	5,800,000	14,000,000	10,000,000	600	1,500	S2
471	Silver carp	Hypophthalmichthys molitrix	17,653	10,000,000	10,000,000	10,000,000	1,747	1,747	S2
472	Bayad	Bagrus bajad	8,335	10,000,000	10,000,000	10,000,000	830	830	GF
473	John dory	Zeus faber	10,447	7,000,000	13,000,000	10,000,000	800	1,500	S1
474	Pangas catfishes nei	Pangasius spp	4,953	9,800,000	10,000,000	10,000,000	488	505	GO
475	Salema	Sarpa salpa	3,854	8,200,000	12,000,000	9,900,000	335	468	GF
476	Pike-perch	Sander lucioperca	18,193	7,900,000	12,000,000	9,800,000	1,540	2,314	S1
477	Greenback horse mackerel	Trachurus declivis	8,265	9,700,000	9,700,000	9,700,000	850	850	S1
478	Blackbanded trevally	Seriolina nigrofasciata	3,199	9,500,000	9,900,000	9,700,000	323	338	S1
479	Croakers nei	Micropogonias spp	2,926	4,700,000	14,000,000	9,600,000	202	620	GG
480	Atlantic moonfish	Selene setapinnis	3,128	9,400,000	9,400,000	9,400,000	333	333	S2
481	Gulf butterfishes nei	Peprilus spp	1,428	5,100,000	13,000,000	9,300,000	106	279	GF[1]
482	Kingcroakers nei	Menticirrhus spp	1,942	4,300,000	14,000,000	9,300,000	136	454	GG
483	Lake(=Common) whitefish	Coregonus clupeaformis	10,500	9,200,000	9,300,000	9,200,000	1,134	1,143	S1
484	Bartail flathead	Platycephalus indicus	3,505	9,100,000	9,100,000	9,100,000	384	384	S1
485	North African catfish	Clarias gariepinus	51,133	8,600,000	9,500,000	9,000,000	5,385	5,955	S3
486	Bluespine unicornfish	Naso unicornis	373	5,700,000	12,000,000	8,700,000	32	66	GC
487	Vimba bream	Vimba vimba	257	6,700,000	11,000,000	8,700,000	24	38	GF
488	Sand steenbras	Lithognathus mormyrus	3,508	7,100,000	10,000,000	8,600,000	347	494	GF
489	Kinneret bleak	Acanthobrama terraesanctae	339	6,700,000	10,000,000	8,600,000	33	50	GF
490	Pink cusk-eel	Genypterus blacodes	38,198	8,400,000	8,400,000	8,400,000	4,536	4,536	S1
491	Angolan dentex	Dentex angolensis	1,349	3,500,000	13,000,000	8,400,000	101	386	S1
492	Greater argentine	Argentina silus	3,297	5,500,000	11,000,000	8,200,000	300	600	S2
493	Stingrays, butterfly rays nei	Dasyatidae	27,017	5,200,000	11,000,000	8,200,000	2,403	5,229	GO
494	Honnibe croaker	Nibea mitsukurii	696	5,700,000	11,000,000	8,200,000	65	123	GF
495	Peruvian moonfish	Selene peruviana	2,414	6,700,000	9,500,000	8,100,000	254	362	GG
496	Picarel	Spicara smaris	315	7,100,000	9,000,000	8,100,000	35	44	GG
497	Atlantic anchoveta	Cetengraulis edentulus	155	6,100,000	9,900,000	8,000,000	16	25	S2
498	Gilthead seabream	Sparus aurata	8,055	8,000,000	8,000,000	8,000,000	1,009	1,009	S1
499	Argentine menhaden	Brevoortia pectinata	1,018	6,400,000	9,400,000	7,900,000	108	158	GG
500	Alestes spp	Alestes spp	330	7,900,000	7,900,000	7,900,000	42	42	GO[1]
501	Largemouth black bass	Micropterus salmoides	1,022	4,400,000	11,000,000	7,900,000	90	230	GO
502	Snooks(=Robalos) nei	Centropomus spp	12,341	2,500,000	13,000,000	7,900,000	928	4,859	GF
503	Bastard halibut	Paralichthys olivaceus	10,520	5,300,000	11,000,000	7,900,000	1,000	2,000	S2
504	Whitefin wolf-herring	Chirocentrus nudus	6,384	3,000,000	13,000,000	7,900,000	500	2,132	S3
505	Round goby	Neogobius melanostomus	221	7,200,000	8,500,000	7,900,000	26	31	GC[1]
506	West African Spanish mackerel	Scomberomorus tritor	3,587	3,600,000	12,000,000	7,800,000	300	1,000	S2
507	Amer. plaice(=Long rough dab)	Hippoglossoides platessoides	8,520	6,100,000	9,400,000	7,700,000	909	1,400	S1
508	Rays and skates nei	Rajidae	31,248	5,500,000	9,900,000	7,700,000	3,147	5,676	GF
509	Black seabream	Spondyliosoma cantharus	7,700	7,700,000	7,700,000	7,700,000	1,000	1,000	S1
510	Japanese halfbeak	Hyporhamphus sajori	621	3,100,000	12,000,000	7,700,000	51	197	GF
511	White seabream	Diplodus sargus	2,962	6,300,000	8,900,000	7,600,000	334	469	GF
512	Silver scabbardfish	Lepidopus caudatus	10,764	4,300,000	11,000,000	7,500,000	1,000	2,500	S1
513	Tusk(=Cusk)	Brosme brosme	24,035	6,200,000	8,900,000	7,500,000	2,710	3,900	S2
514	Atractosteus spp	Atractosteus spp	345	5,000,000	10,000,000	7,500,000	34	70	GC
515	Tub gurnard	Chelidonichthys lucerna	3,986	7,500,000	7,500,000	7,500,000	532	532	S2

516	Caspian roach	Rutilus caspicus	2,222	7,400,000	7,400,000	7,400,000	300	300	GG
517	Giant featherback	Chitala lopis	2,949	7,200,000	7,600,000	7,400,000	386	412	GO
518	European seabass	Dicentrarchus labrax	8,820	7,400,000	7,400,000	7,400,000	1,200	1,200	S1
519	Sablefish	Anoplopoma fimbria	22,058	4,900,000	9,700,000	7,300,000	2,268	4,536	S1
520	Toli shad	Tenualosa toli	2,981	3,300,000	11,000,000	7,200,000	266	915	GG
521	Brachyplatystoma rousseauxii	Brachyplatystoma rousseauxii	6,084	6,700,000	7,600,000	7,200,000	800	905	GG
522	Panga seabream	Pterogymnus laniarius	2,930	5,900,000	8,300,000	7,100,000	353	494	GF
523	Oilfish	Ruvettus pretiosus	14,196	4,700,000	9,500,000	7,100,000	1,500	3,000	GF
524	Alestes baremoze	Alestes baremoze	411	7,100,000	7,100,000	7,100,000	58	58	GO[1]
525	Megrim	Lepidorhombus whiffiagonis	12,210	5,600,000	8,400,000	7,000,000	1,455	2,164	GF
526	Silver warehou	Seriolella punctata	10,513	3,500,000	11,000,000	7,000,000	1,000	3,000	GG
527	Rough scad	Trachurus lathami	1,793	7,000,000	7,000,000	7,000,000	256	256	S3
528	Thorntooth grenadier	Lepidorhynchus denticulatus	3,696	5,100,000	8,800,000	6,900,000	420	730	GF
529	Potamorhina laticeps	Potamorhina laticeps	291	6,900,000	6,900,000	6,900,000	42	42	GO
530	Kamchatka flounder	Atheresthes evermanni	8,065	6,400,000	7,400,000	6,900,000	1,093	1,268	GG
531	Southern red snapper	Lutjanus purpureus	8,155	3,600,000	10,000,000	6,800,000	815	2,266	GG
532	Scup	Stenotomus chrysops	4,529	4,500,000	9,100,000	6,800,000	500	1,000	S1
533	Spotted sicklefish	Drepane punctata	2,782	4,600,000	8,800,000	6,700,000	315	602	GG
534	White grunt	Haemulon plumierii	1,013	4,400,000	8,900,000	6,700,000	114	228	GF
535	Walleye	Sander vitreus	8,015	4,400,000	8,900,000	6,700,000	900	1,814	S1
536	Unicorn cod	Bregmaceros mcclellandi	2,165	3,200,000	10,000,000	6,600,000	215	684	GO
537	Bastard halibuts nei	Paralichthys spp	8,758	4,200,000	9,000,000	6,600,000	978	2,096	GG
538	Bluefin gurnard	Chelidonichthys kumu	4,830	3,400,000	9,700,000	6,600,000	500	1,400	S1
539	Eeltail catfishes	Plotosus spp	3,212	6,400,000	6,700,000	6,500,000	483	500	GO
540	Hakes nei	Merluccius spp	3,478	4,900,000	9,200,000	7,000,000	379	708	GG
541	Stripped weakfish	Cynoscion guatucupa	18,186	6,000,000	6,600,000	6,300,000	2,756	3,033	GG
542	Gars nei	Lepisosteidae	270	4,100,000	8,500,000	6,300,000	32	66	GC
543	Atlantic Spanish mackerel	Scomberomorus maculatus	8,510	3,100,000	9,400,000	6,300,000	907	2,722	S1
544	Freshwater minnow	Zacco platypus	296	4,900,000	7,500,000	6,200,000	40	60	GF
545	Weevers nei	Trachinus spp	1,412	6,100,000	6,100,000	6,100,000	230	230	GG
546	Yellowtail snapper	Ocyurus chrysurus	8,317	6,100,000	6,100,000	6,100,000	1,361	1,361	S1
547	Greater weever	Trachinus draco	1,399	6,100,000	6,100,000	6,100,000	230	230	S1
548	Law croaker	Pseudotolithus senegallus	2,192	6,100,000	6,100,000	6,100,000	361	361	S1
549	European eel	Anguilla anguilla	5,390	5,400,000	6,700,000	6,100,000	800	1,000	S1
550	Ling	Molva molva	40,926	6,000,000	6,000,000	6,000,000	6,800	6,800	S1
551	Fat sleeper	Dormitator maculatus	166	5,400,000	6,500,000	6,000,000	25	31	GC[1]
552	Blue shark	Prionace glauca	90,615	5,500,000	6,300,000	5,900,000	14,412	16,562	S1
553	Bocon toadfish	Amphichthys cryptocentrus	265	3,900,000	7,900,000	5,900,000	34	69	GC
554	Dogfish sharks nei	Squalidae	8,458	1,500,000	10,000,000	5,800,000	842	5,687	GF
555	Winter flounder	Pseudopleuronectes americanus	4,480	5,000,000	6,400,000	5,700,000	700	900	S1
556	Serra Spanish mackerel	Scomberomorus brasiliensis	4,322	2,500,000	8,800,000	5,600,000	491	1,744	S2
557	Pargo breams nei	Pagrus spp	7,169	5,100,000	6,200,000	5,600,000	1,154	1,414	GG
558	Rubyfish	Plagiogeneion rubiginosum	472	1,600,000	9,400,000	5,500,000	50	300	GF
559	West coast sole	Austroglossus microlepis	3,028	870,000	10,000,000	5,500,000	300	3,500	GF
560	Bloch's gizzard shad	Nematalosa nasus	210	4,700,000	6,300,000	5,500,000	33	45	GF
561	Vadigo	Campogramma glaycos	516	2,700,000	8,200,000	5,500,000	63	191	GF
562	Alfonsinos nei	Beryx spp	7,053	4,400,000	6,500,000	5,400,000	1,090	1,619	GG
563	Humpback grouper	Cromileptes altivelis	7,169	2,400,000	8,100,000	5,300,000	884	2,946	GF
564	Arawana	Osteoglossum bicirrhosum	1,557	5,200,000	5,300,000	5,200,000	296	299	GF
565	Threadsail filefish	Stephanolepis cirrhifer	2,238	5,000,000	5,400,000	5,200,000	414	445	GO
566	Sampa	Heterobranchus longifilis	2,209	5,200,000	5,200,000	5,200,000	424	427	GF
567	Southwest Atlantic butterfish	Stromateus brasiliensis	1,055	4,100,000	6,300,000	5,200,000	169	258	GF
568	Warty dory	Allocyttus verrucosus	309	3,900,000	6,300,000	5,100,000	49	79	GO
569	Trumpeters nei	Latridae	641	2,800,000	7,200,000	5,000,000	89	226	GO
570	Spotted barb	Puntius binotatus	128	3,900,000	6,100,000	5,000,000	21	33	GF
571	Acoupa weakfish	Cynoscion acoupa	19,571	5,000,000	5,000,000	5,000,000	3,900	3,900	S1
572	Swordfish	Xiphias gladius	111,016	1,900,000	8,100,000	5,000,000	13,738	60,000	S1
573	Pacific jack mackerel	Trachurus symmetricus	1,237	2,500,000	7,400,000	4,900,000	167	500	S2
574	Forkbeard	Phycis phycis	1,598	2,200,000	7,500,000	4,900,000	212	728	GF
575	Red codling	Pseudophycis bachus	5,872	2,300,000	7,300,000	4,800,000	800	2,500	S1
576	Hound needlefish	Tylosurus crocodilus	249	4,800,000	4,800,000	4,800,000	52	52	GF
577	Mackerel icefish	Champsocephalus gunnari	1,943	3,200,000	6,400,000	4,800,000	305	611	GO[1]
578	Monkfishes nei	Lophius spp	32,043	3,900,000	5,600,000	4,800,000	5,716	8,160	GG
579	Freshwater perches nei	Lates spp	7,486	1,500,000	8,000,000	4,800,000	936	4,927	GG
580	Loweye catfishes nei	Hypophthalmus spp	3,983	4,400,000	5,000,000	4,700,000	800	905	GF
581	Leporinus obtusidens	Leporinus obtusidens	209	4,700,000	4,700,000	4,700,000	45	45	GO
582	Common silver-biddy	Gerres oyena	591	2,600,000	6,600,000	4,600,000	90	227	GO
583	Sorubims nei	Pseudoplatystoma spp	3,853	4,300,000	4,800,000	4,500,000	800	905	GF
584	Transparent goby	Aphia minuta	108	4,300,000	4,700,000	4,500,000	23	25	GC[1]
585	Tilapia shiranus	Oreochromis shiranus	1,745	3,000,000	6,000,000	4,500,000	292	572	GG
586	Chub	Leuciscus cephalus	130	3,500,000	5,500,000	4,500,000	24	37	GF
587	Ridge scaled rattail	Macrourus carinatus	3,945	3,500,000	5,600,000	4,500,000	709	1,143	GF[1]
588	Shortnose greeneye	Chlorophthalmus agassizi	141	4,000,000	5,000,000	4,500,000	28	35	GO
589	Yellow jack	Carangoides bartholomaei	381	2,200,000	6,800,000	4,500,000	56	174	GF
590	Broadgilled hagfish	Eptatretus cirrhatus	204	3,000,000	6,000,000	4,500,000	34	69	GA
591	Blue ling	Molva dypterygia	8,851	1,700,000	7,200,000	4,500,000	1,234	5,121	S3
592	Striped piggy	Pomadasys stridens	656	4,400,000	4,400,000	4,400,000	149	149	GG
593	Tanaka's snailfish	Liparis tanakae	2,055	4,000,000	4,700,000	4,300,000	442	511	GO
594	Blackmouth catshark	Galeus melastomus	742	4,300,000	4,300,000	4,300,000	172	172	S2
595	Northern red snapper	Lutjanus campechanus	5,033	2,200,000	6,400,000	4,300,000	790	2,290	S1
596	Cypselurus spp	Cypselurus spp	354	2,100,000	6,300,000	4,200,000	56	169	GO
597	Common nase	Chondrostoma nasus	111	3,200,000	5,100,000	4,200,000	22	34	GF
598	Burbot	Lota lota	3,545	1,200,000	7,100,000	4,100,000	500	3,000	S1
599	Filefishes, leatherjackets nei	Monacanthidae	2,127	4,100,000	4,100,000	4,100,000	517	520	GO
600	Golden grey mullet	Liza aurata	1,116	740,000	7,400,000	4,100,000	150	1,500	S1
601	Summer flounder	Paralichthys dentatus	5,285	2,300,000	5,800,000	4,100,000	907	2,268	S1
602	River eels nei	Anguilla spp	3,377	3,300,000	4,800,000	4,100,000	703	1,008	GG
603	Coho(=Silver) salmon	Oncorhynchus kisutch	22,010	4,100,000	4,100,000	4,100,000	5,400	5,400	S1
604	Glass catfishes	Kryptopterus spp	15,278	2,300,000	5,800,000	4,100,000	2,624	6,700	GF
605	Yellowtail rockfish	Sebastes flavidus	1,452	3,600,000	4,400,000	4,000,000	328	399	GG
606	Smooth-hounds nei	Mustelus spp	11,287	2,900,000	5,200,000	4,000,000	2,191	3,937	GG
607	Pacific red snapper	Lutjanus peru	4,870	2,200,000	5,800,000	4,000,000	838	2,220	GG
608	Gulf kingcroaker	Menticirrhus littoralis	827	1,800,000	6,100,000	4,000,000	136	454	S1
609	River lamprey	Lampetra fluviatilis	232	3,900,000	3,900,000	3,900,000	60	60	S1
610	Flagfin mojarra	Eucinostomus melanopterus	482	2,200,000	5,500,000	3,900,000	88	215	GO
611	Twobar seabream	Acanthopagrus bifasciatus	4,667	3,800,000	3,800,000	3,800,000	1,220	1,220	S1
612	Haffara seabream	Rhabdosargus haffara	1,481	3,200,000	4,400,000	3,800,000	335	470	GF
613	Indian pompano	Trachinotus mookalee	324	1,900,000	5,700,000	3,800,000	57	171	GF
614	Nursehound	Scyliorhinus stellaris	648	3,800,000	3,800,000	3,800,000	172	172	GF
615	Catsharks, nursehounds nei	Scyliorhinus spp	642	3,700,000	3,700,000	3,700,000	172	172	GF
616	Redfish	Centroberyx affinis	937	1,600,000	5,900,000	3,700,000	160	600	S2
617	Warehou nei	Seriolella spp	5,540	1,800,000	5,500,000	3,700,000	1,000	3,000	GG
618	Forkbeards nei	Phycis spp	1,036	1,600,000	5,700,000	3,600,000	183	637	GF
619	Catfishes nei	Ictalurus spp	5,414	3,300,000	3,900,000	3,600,000	1,381	1,637	GG

620	Elongate tigerfish	Hydrocynus forskahlii	276	3,600,000	3,600,000	3,600,000	77	77	GO[1]
621	Widow rockfish	Sebastes entomelas	1,822	1,000,000	6,100,000	3,500,000	300	1,814	S2
622	Oscar	Astronotus ocellatus	1,128	2,600,000	4,500,000	3,500,000	253	432	GF
623	Patagonian toothfish	Dissostichus eleginoides	26,483	2,600,000	4,400,000	3,500,000	6,000	10,000	S1
624	Parore	Girella tricuspidata	453	2,000,000	5,100,000	3,500,000	89	230	GO
625	Saddled seabream	Oblada melanura	1,389	2,900,000	4,100,000	3,500,000	338	478	GF
626	White mullet	Mugil curema	3,830	2,800,000	4,200,000	3,500,000	910	1,367	GG
627	Sweetlips, rubberlips nei	Plectorhinchus spp	1,868	2,300,000	4,700,000	3,500,000	400	800	GG
628	Aimara	Hoplerythrinus unitaeniatus	193	3,500,000	3,500,000	3,500,000	55	55	GO
629	Megrims nei	Lepidorhombus spp	6,055	2,800,000	4,100,000	3,500,000	1,460	2,160	GF
630	Yellowbar angelfish	Pomacanthus maculosus	444	2,000,000	4,900,000	3,500,000	90	225	GO
631	Malabar trevally	Carangoides malabaricus	333	1,700,000	5,200,000	3,400,000	65	191	GF
632	Benguela hake	Merluccius polli	3,538	2,900,000	3,900,000	3,400,000	900	1,200	S2
633	White grouper	Epinephelus aeneus	4,282	3,400,000	3,400,000	3,400,000	1,250	1,250	S1
634	Peruvian morwong	Cheilodactylus variegatus	434	1,900,000	4,900,000	3,400,000	89	227	GO
635	American angler	Lophius americanus	14,741	2,200,000	4,600,000	3,400,000	3,182	6,818	S1
636	Parrotfish	Sparisoma cretense	423	1,900,000	4,900,000	3,400,000	87	218	GO
637	Turbot	Scophthalmus maximus	6,363	3,300,000	3,500,000	3,400,000	1,827	1,925	S2
638	Pollack	Pollachius pollachius	9,967	2,200,000	4,500,000	3,400,000	2,200	4,500	GG
639	Black skipjack	Euthynnus lineatus	6,020	2,700,000	4,000,000	3,300,000	1,520	2,200	S1
640	Cardinalfishes, etc. nei	Apogonidae	441	1,900,000	4,800,000	3,300,000	92	237	GO
641	Angler(=Monk)	Lophius piscatorius	29,789	3,300,000	3,300,000	3,300,000	9,000	9,000	S1
642	Bluespot mullet	Valamugil seheli	2,585	1,300,000	5,200,000	3,200,000	500	2,000	S2
643	Indian driftfish	Ariomma indica	175	2,800,000	3,400,000	3,100,000	52	63	GO
644	King mackerel	Scomberomorus cavalla	11,673	1,300,000	5,100,000	3,200,000	2,268	9,072	S1
645	Brazilian menhaden	Brevoortia aurea	1,038	1,200,000	5,200,000	3,200,000	200	850	S2
646	Striped weakfish	Cynoscion striatus	7,364	2,900,000	3,400,000	3,200,000	2,148	2,518	GG
647	Chinook(=Spring=King) salmon	Oncorhynchus tshawytscha	9,415	1,100,000	5,200,000	3,200,000	1,814	8,341	S1
648	Tigertooth croaker	Otolithes ruber	7,854	3,100,000	3,100,000	3,100,000	2,500	2,500	S1
649	So-iuy mullet	Mugil soiuy	3,430	2,500,000	3,800,000	3,100,000	910	1,367	GG
650	Seabasses nei	Dicentrarchus spp	3,742	3,100,000	3,100,000	3,100,000	1,200	1,200	GG
651	Black-streaked monocle bream	Scolopsis taeniata	590	2,400,000	3,800,000	3,100,000	154	250	GF
652	Slender silver-biddy	Gerres oblongus	368	1,800,000	4,400,000	3,100,000	84	210	GO
653	Blackchin tilapia	Sarotherodon melanotheron	953	2,200,000	3,800,000	3,000,000	250	432	GF
654	Lane snapper	Lutjanus synagris	3,586	1,600,000	4,400,000	3,000,000	815	2,266	GG
655	Shortspine thornyhead	Sebastolobus alascanus	1,060	2,700,000	3,200,000	3,000,000	326	391	GF
656	Common mora	Mora moro	1,315	1,500,000	4,400,000	3,000,000	296	895	GF
657	Silvery pout	Gadiculus argenteus	878	1,300,000	4,600,000	2,900,000	192	676	GF
658	Paralabrax spp	Paralabrax spp	3,912	1,300,000	4,500,000	2,900,000	875	2,919	GF
659	Wels(=Som) catfish	Silurus glanis	10,891	1,600,000	4,200,000	2,900,000	2,624	6,700	S1
660	Pontic shad	Alosa pontica	777	2,600,000	3,200,000	2,900,000	246	298	GG
661	Small sandeel	Ammodytes tobianus	29	2,900,000	2,900,000	2,900,000	10	10	GG
662	Annular seabream	Diplodus annularis	1,111	2,300,000	3,300,000	2,800,000	336	478	GF
663	Plownose chimaera	Callorhinchus callorynchus	2,309	2,700,000	2,700,000	2,700,000	852	852	GG
664	Pacific rudderfish	Psenopsis anomala	5,648	1,500,000	3,800,000	2,700,000	1,468	3,669	GF
665	Snubnose emperor	Lethrinus borbonicus	901	2,000,000	3,400,000	2,700,000	264	459	GO[1]
666	Leaping African mullet	Mugil capurrii	2,941	2,100,000	3,200,000	2,700,000	911	1,368	GG
667	Glass schilbid	Parailia pellucida	1,435	2,600,000	2,700,000	2,700,000	525	545	GO
668	American harvestfish	Peprilus paru	525	2,000,000	3,300,000	2,700,000	157	260	GF[1]
669	Shrimp scad	Alepes djedaba	250	1,300,000	4,000,000	2,700,000	62	187	GF
670	Mystus nigriceps	Mystus nigriceps	2,194	2,600,000	2,600,000	2,600,000	830	830	GF
671	Corocoro grunt	Orthopristis ruber	395	1,700,000	3,500,000	2,600,000	113	226	GF
672	African obscure snakehead	Parachanna obscura	411	2,600,000	2,600,000	2,600,000	156	158	S1
673	Greasy grouper	Epinephelus tauvina	8,467	1,200,000	3,900,000	2,600,000	2,158	7,107	GG
674	Lake cisco	Coregonus artedi	773	1,700,000	3,400,000	2,600,000	227	454	S1
675	Wedge sole	Dicologlossa cuneata	1,405	400,000	4,700,000	2,500,000	300	3,500	GF
676	Argentine seabass	Acanthistius brasilianus	3,320	1,200,000	3,900,000	2,500,000	847	2,868	GF
677	Petrale sole	Eopsetta jordani	2,105	1,800,000	3,200,000	2,500,000	667	1,175	S1
678	Common warehou	Seriolella brama	3,690	1,200,000	3,700,000	2,500,000	1,000	3,000	S1
679	John's snapper	Lutjanus johnii	2,929	1,300,000	3,500,000	2,400,000	826	2,244	GG
680	Narrownose smooth-hound	Mustelus schmitti	7,078	1,800,000	3,000,000	2,400,000	2,391	3,891	GG
681	Choicy ruff	Seriolella porosa	3,567	1,200,000	3,600,000	2,400,000	1,000	3,000	GG
682	Leporinus spp	Leporinus spp	99	2,400,000	2,400,000	2,400,000	42	42	GO
683	Roughhead grenadier	Macrourus berglax	1,647	1,800,000	3,000,000	2,400,000	557	934	GF[1]
684	Longspine snipefish	Macroramphosus scolopax	106	1,500,000	3,100,000	2,300,000	34	69	GC
685	Black seabass	Centropristis striata	1,414	1,600,000	3,100,000	2,300,000	454	908	S1
686	Orange-spotted grouper	Epinephelus coioides	7,773	1,100,000	3,600,000	2,300,000	2,156	7,292	GG
687	Shorthead drum	Larimus breviceps	196	1,600,000	3,000,000	2,300,000	65	123	GF
688	Bowfin	Amia calva	103	1,500,000	3,100,000	2,300,000	34	68	GC
689	Red hake	Urophycis chuss	1,541	1,100,000	3,400,000	2,300,000	450	1,400	S1
690	Catsharks, etc. nei	Scyliorhinidae	388	2,300,000	2,300,000	2,300,000	172	172	GF
691	West African ladyfish	Elops lacerta	2,441	1,800,000	2,700,000	2,200,000	907	1,361	GG
692	Longtail Southern cod	Patagonotothen ramsayi	18,689	1,700,000	2,800,000	2,200,000	6,759	10,990	GF
693	Bearded brotula	Brotula barbata	2,420	1,800,000	2,700,000	2,200,000	909	1,364	S2
694	Striped bonito	Sarda orientalis	3,536	880,000	3,500,000	2,200,000	1,000	4,000	S1
695	Giant catfish	Netuma thalassina	1,074	830,000	3,600,000	2,200,000	300	1,300	S2
696	Devil anglerfish	Lophius vomerinus	14,325	1,800,000	2,600,000	2,200,000	5,489	8,082	GG
697	Red grouper	Epinephelus morio	3,300	730,000	3,600,000	2,200,000	907	4,536	S1
698	Atlantic wolffish	Anarhichas lupus	14,844	2,200,000	2,200,000	2,200,000	6,804	6,804	S1
699	Various sharks nei	Selachimorpha (Pleurotremata)	10,255	1,000,000	3,300,000	2,200,000	3,080	10,105	GC
700	Fire eel	Mastacembelus erythrotaenia	101	1,400,000	2,900,000	2,200,000	35	70	GC
701	Requiem sharks nei	Carcharhinidae	33,662	2,000,000	2,300,000	2,200,000	14,536	16,775	GF
702	Grenadiers nei	Macrourus spp	1,860	1,600,000	2,700,000	2,200,000	698	1,129	GF[1]
703	Jobfishes nei	Pristipomoides spp	2,778	1,700,000	2,600,000	2,100,000	1,068	1,639	GF
704	Dark ghost shark	Hydrolagus novaezealandiae	1,813	2,100,000	2,100,000	2,100,000	852	852	GO
705	Argentine goatfish	Mullus argentinae	220	1,100,000	3,100,000	2,100,000	70	200	S2
706	Lampreys nei	Petromyzontidae	127	2,100,000	2,100,000	2,100,000	60	60	GF
707	Common snook	Centropomus undecimalis	3,240	680,000	3,500,000	2,100,000	918	4,767	GF
708	Vermilion snapper	Rhomboplites aurorubens	2,705	1,700,000	2,500,000	2,100,000	1,067	1,630	GF
709	Navaga(=Wachna cod)	Eleginus navaga	544	1,400,000	2,700,000	2,000,000	200	400	GG
710	White hake	Urophycis tenuis	5,710	1,600,000	2,500,000	2,000,000	2,268	3,656	S1
711	Yellowspotted trevally	Carangoides fulvoguttatus	193	1,000,000	3,000,000	2,000,000	64	192	GF
712	Antarctic rockcods, noties nei	Nototheniidae	16,649	1,500,000	2,500,000	2,000,000	6,770	11,016	GF
713	Indo-Pacific tarpon	Megalops cyprinoides	1,609	870,000	3,100,000	2,000,000	519	1,850	S3
714	Pacific halibut	Hippoglossus stenolepis	30,727	1,700,000	2,300,000	2,000,000	13,640	18,180	S1
715	Pacific piquitinga	Lile stolifera	72	1,600,000	2,300,000	2,000,000	32	44	GF
716	Crevalle jack	Caranx hippos	9,576	2,000,000	2,000,000	2,000,000	4,910	4,910	S1
717	Sea trout	Salmo trutta	4,295	1,100,000	2,800,000	1,900,000	1,560	3,760	S1
718	Malayan leaffish	Pristolepis fasciata	229	1,100,000	2,700,000	1,900,000	83	210	GO
719	Anglerfishes nei	Lophiidae	12,853	1,600,000	2,200,000	1,900,000	5,818	8,193	GF
720	Congo dentex	Dentex congoensis	601	990,000	2,800,000	1,900,000	218	609	GG
721	Brill	Scophthalmus rhombus	2,798	930,000	2,800,000	1,900,000	1,000	3,000	S2
722	Alfonsino	Beryx decadactylus	2,484	1,200,000	2,500,000	1,900,000	1,000	2,000	S1
723	Spotted sorubim	Pseudoplatystoma corruscans	1,535	1,700,000	1,900,000	1,800,000	800	905	GF

724	Kingklip	Genypterus capensis	8,171	1,800,000	1,800,000	1,800,000	4,536	4,536	S1
725	Sky emperor	Lethrinus mahsena	629	1,300,000	2,300,000	1,800,000	279	469	GO[1]
726	Piranhas nei	Serrasalmus spp	6,540	1,500,000	2,100,000	1,800,000	3,160	4,300	GF
727	Manduba	Ageneiosus inermis	937	1,700,000	1,800,000	1,800,000	522	542	GO
728	Cape gurnard	Chelidonichthys capensis	658	1,300,000	2,200,000	1,800,000	300	500	S2
729	Mediterranean sand eel	Gymnammodytes cicerelus	18	1,700,000	1,700,000	1,700,000	10	10	GF
730	Blackspot(=red) seabream	Pagellus bogaraveo	1,562	1,700,000	1,700,000	1,700,000	900	900	S1
731	Blue tilapia	Oreochromis aureus	2,067	1,100,000	2,300,000	1,700,000	907	1,814	S1
732	Sleek lates	Lates stappersii	2,650	530,000	2,800,000	1,700,000	942	4,982	GG
733	Carpenter seabream	Argyrozona argyrozona	635	1,400,000	1,900,000	1,700,000	329	449	GF
734	Brycinus leuciscus	Brycinus leuciscus	66	1,700,000	1,700,000	1,700,000	39	39	GG
735	Ghost shark	Callorhinchus milii	1,413	1,700,000	1,700,000	1,700,000	852	852	GG
736	Baird's slickhead	Alepocephalus bairdii	1,543	1,300,000	2,000,000	1,700,000	774	1,169	S2
737	Peruvian weakfish	Cynoscion analis	4,151	1,500,000	1,800,000	1,700,000	2,359	2,687	GG
738	Lefteye flounders nei	Bothidae	1,327	1,000,000	2,300,000	1,600,000	587	1,290	GO
739	Lebranche mullet	Mugil liza	2,277	1,200,000	2,000,000	1,600,000	1,126	1,849	S1
740	Toadfishes, etc. nei	Batrachoididae	68	1,100,000	2,200,000	1,600,000	31	65	GC
741	Gulf parrotfish	Scarus persicus	197	920,000	2,300,000	1,600,000	85	213	GO
742	Cornetfish	Fistularia tabacaria	76	1,100,000	2,200,000	1,600,000	35	70	GC
743	Kumakuma	Brachyplatystoma filamentosum	1,371	1,500,000	1,700,000	1,600,000	800	905	GG
744	Brushtooth lizardfish	Saurida undosquamis	231	800,000	2,400,000	1,600,000	97	290	S2
745	Xenocharax spilurus	Xenocharax spilurus	67	1,600,000	1,600,000	1,600,000	42	42	GO
746	Psectrogaster amazonica	Psectrogaster amazonica	66	1,600,000	1,600,000	1,600,000	42	42	GO
747	Indo-Pacific sailfish	Istiophorus platypterus	28,293	1,000,000	2,100,000	1,600,000	13,640	27,270	S1
748	Ratfishes nei	Hydrolagus spp	1,325	1,600,000	1,600,000	1,600,000	852	852	GO
749	Boarfishes nei	Caproidae	57	1,200,000	1,900,000	1,600,000	30	48	GF
750	Whitespotted conger	Conger myriaster	14,080	1,500,000	1,600,000	1,500,000	9,077	9,100	GG
751	Smallscale archerfish	Toxotes microlepis	181	870,000	2,200,000	1,500,000	83	209	GO
752	Stargazer	Uranoscopus scaber	177	1,500,000	1,500,000	1,500,000	117	117	S1
753	Common two-banded seabream	Diplodus vulgaris	594	1,300,000	1,800,000	1,500,000	337	470	GF
754	European conger	Conger conger	13,766	1,500,000	1,500,000	1,500,000	9,100	9,100	S1
755	Peruvian rock seabass	Paralabrax humeralis	1,927	700,000	2,300,000	1,500,000	829	2,772	GF
756	Florida pompano	Trachinotus carolinus	152	750,000	2,300,000	1,500,000	67	203	GF
757	Honeycomb grouper	Epinephelus merra	4,943	680,000	2,300,000	1,500,000	2,159	7,287	GG
758	Common dentex	Dentex dentex	1,469	1,500,000	1,500,000	1,500,000	1,000	1,000	S1
759	Green humphead parrotfish	Bolbometopon muricatum	184	800,000	2,100,000	1,400,000	88	229	GO
760	Plain bonito	Orcynopsis unicolor	786	910,000	1,900,000	1,400,000	410	862	GF
761	Armless snake eel	Dalophis imberbis	1,246	420,000	2,400,000	1,400,000	518	2,989	GO
762	Payara	Hydrolycus scomberoides	59	1,400,000	1,400,000	1,400,000	42	42	GO
763	Spotted weakfish	Cynoscion nebulosus	1,692	1,400,000	1,400,000	1,400,000	1,225	1,225	S1
764	Atlantic halibut	Hippoglossus hippoglossus	5,981	110,000	2,600,000	1,400,000	2,300	56,000	S2
765	Ocean whitefish	Caulolatilus princeps	2,039	1,200,000	1,500,000	1,400,000	1,316	1,766	GF[1]
766	Japanese eel	Anguilla japonica	354	550,000	2,100,000	1,300,000	165	638	S1
767	Cape dory	Zeus capensis	1,401	930,000	1,800,000	1,300,000	800	1,500	GG
768	Red bandfish	Cepola macrophthalma	172	750,000	1,900,000	1,300,000	91	229	GO
769	Tench	Tinca tinca	2,694	690,000	1,900,000	1,300,000	1,400	3,900	S1
770	Bluestripe herring	Herklotsichthys quadrimaculat.	22	1,300,000	1,300,000	1,300,000	17	17	S1
771	Wolffishes(=Catfishes) nei	Anarhichas spp	8,761	1,300,000	1,300,000	1,300,000	6,804	6,804	GG
772	Randall's threadfin bream	Nemipterus randalli	250	1,000,000	1,500,000	1,300,000	167	244	GG
773	Cobia	Rachycentron canadum	11,909	650,000	1,900,000	1,300,000	6,364	18,182	S1
774	Atlantic emperor	Lethrinus atlanticus	538	1,000,000	1,500,000	1,300,000	368	515	S3
775	Blue catfish	Ictalurus furcatus	2,245	1,200,000	1,200,000	1,200,000	1,800	1,800	S1
776	White warehou	Seriolella caerulea	1,863	620,000	1,900,000	1,200,000	1,000	3,000	GG
777	Brazilian groupers nei	Mycteroperca spp	2,322	250,000	2,200,000	1,200,000	1,042	9,223	GG
778	Yellowstripe goatfish	Mulloidichthys flavolineatus	154	1,200,000	1,200,000	1,200,000	125	125	S1
779	New Zealand blue cod	Parapercis colias	2,256	1,200,000	1,200,000	1,200,000	1,820	1,877	GF
780	Cachama	Colossoma macropomum	4,434	1,000,000	1,400,000	1,200,000	3,160	4,300	S2
781	Spotted wolffish	Anarhichas minor	8,275	1,200,000	1,200,000	1,200,000	6,804	6,804	GG
782	Eagle rays nei	Myliobatidae	3,914	750,000	1,700,000	1,200,000	2,356	5,194	GO
783	Argentinian sandperch	Pseudopercis semifasciata	2,228	1,200,000	1,200,000	1,200,000	1,820	1,877	S3
784	Deep-water Cape hake	Merluccius paradoxus	961	1,200,000	1,200,000	1,200,000	800	800	S1
785	Amazon sailfin catfish	Pterygoplichthys pardalis	638	1,200,000	1,200,000	1,200,000	522	542	GO
786	Humphead wrasse	Cheilinus undulatus	1,032	420,000	1,900,000	1,200,000	530	2,466	GF
787	Velvet leatherjacket	Parika scaber	567	1,100,000	1,200,000	1,200,000	477	497	GO
788	Ruff	Arripis georgianus	440	130,000	2,200,000	1,200,000	200	3,500	GG
789	Sea lamprey	Petromyzon marinus	68	1,100,000	1,100,000	1,100,000	60	60	GF
790	Hogfish	Lachnolaimus maximus	864	390,000	1,900,000	1,100,000	462	2,227	GF
791	Ladyfish	Elops saurus	1,224	900,000	1,300,000	1,100,000	907	1,361	S1
792	Spiky oreo	Neocyttus rhomboidalis	93	860,000	1,400,000	1,100,000	67	108	GO
793	Southern meagre(=Mulloway)	Argyrosomus hololepidotus	11,524	720,000	1,500,000	1,100,000	7,561	16,025	S1
794	Freshwater breams nei	Abramis spp	3,978	1,100,000	1,100,000	1,100,000	3,600	3,600	GG
795	Mantas, devil rays nei	Mobulidae	3,590	690,000	1,500,000	1,100,000	2,365	5,207	GO
796	Conger eels, etc. nei	Congridae	10,029	1,100,000	1,100,000	1,100,000	9,076	9,100	GF
797	Pomfrets, ocean breams nei	Bramidae	946	630,000	1,600,000	1,100,000	601	1,503	GF
798	Stumpnoses nei	Rhabdosargus spp	411	890,000	1,300,000	1,100,000	329	461	GF
799	Rainbow trout	Oncorhynchus mykiss	2,565	860,000	1,300,000	1,100,000	2,000	3,000	S2
800	Velvety cichlids	Astronotus spp	347	770,000	1,300,000	1,100,000	258	450	GF
801	Grey triggerfish	Balistes carolinensis	511	1,000,000	1,000,000	1,000,000	491	491	S1
802	Southern bluefin tuna	Thunnus maccoyii	13,490	160,000	1,900,000	1,000,000	7,000	86,600	S1
803	Barred grunt	Conodon nobilis	366	610,000	1,500,000	1,000,000	250	600	S2
804	Pilotfish	Naucrates ductor	99	520,000	1,500,000	1,000,000	65	190	GF
805	Northern wolffish	Anarhichas denticulatus	6,947	1,000,000	1,000,000	1,000,000	6,804	6,804	GG
806	Bluespotted seabream	Pagrus caeruleostictus	468	470,000	1,600,000	1,000,000	300	1,000	S2
807	Tripletail	Lobotes surinamensis	127	580,000	1,400,000	1,000,000	88	220	GO
808	Cyclocheilichthys armatus	Cyclocheilichthys armatus	27	790,000	1,200,000	1,000,000	22	35	GF
809	African scraping feeder	Varicorhinus beso	26	780,000	1,200,000	1,000,000	21	33	GF
810	Abu mullet	Liza abu	1,160	700,000	1,300,000	1,000,000	889	1,655	GG
811	Orange-striped emperor	Lethrinus obsoletus	321	720,000	1,300,000	1,000,000	250	449	GO[1]
812	Greenland cod	Gadus ogac	470	360,000	1,600,000	980,000	293	1,298	GG
813	Canary drum(=Baardman)	Umbrina canariensis	2,357	980,000	980,000	980,000	2,400	2,400	S1
814	Giant stargazer	Kathetostoma giganteum	3,205	360,000	1,600,000	980,000	2,000	9,000	S1
815	Banded yellowfish	Centriscops humerosus	43	640,000	1,300,000	970,000	33	68	GC
816	Channel catfish	Ictalurus punctatus	1,057	780,000	1,200,000	970,000	907	1,361	S1
817	Barred sorubim	Pseudoplatystoma fasciatum	813	900,000	1,000,000	960,000	800	905	GF
818	Porgies	Calamus spp	394	790,000	1,100,000	960,000	351	496	GF
819	Freshwater drum	Aplodinotus grunniens	567	620,000	1,200,000	940,000	454	907	S1
820	Slender rainbow sardine	Dussumieria elopsoides	15	940,000	940,000	940,000	16	16	S1
821	Norway redfish	Sebastes viviparus	339	840,000	1,000,000	930,000	334	403	GG
822	Squeteague(=Gray weakfish)	Cynoscion regalis	560	620,000	1,200,000	930,000	454	907	S1
823	Black cardinal fish	Epigonus telescopus	1,580	590,000	1,300,000	920,000	1,260	2,700	S1
824	Pompano	Trachinotus ovatus	89	460,000	1,400,000	910,000	65	193	GF
825	Gummy shark	Mustelus antarcticus	2,570	650,000	1,200,000	910,000	2,203	3,934	GG
826	Cuckoo ray	Raja naevus	3,214	600,000	1,200,000	910,000	2,641	5,342	GG
827	Little skate	Raja erinacea	2,911	570,000	1,200,000	900,000	2,374	5,124	GG

828	Pickhandle barracuda	Sphyraena jello	765	260,000	1,500,000	890,000	500	3,000	S2
829	Sohal surgeonfish	Acanthurus sohal	39	580,000	1,200,000	880,000	33	67	GC
830	English sole	Pleuronectes vetulus	642	580,000	1,100,000	830,000	598	1,100	GG
831	Spotted rose snapper	Lutjanus guttatus	990	440,000	1,200,000	820,000	830	2,237	GG
832	Morays nei	Muraenidae	726	250,000	1,400,000	820,000	523	2,957	GO
833	Blonde ray	Raja brachyura	1,395	230,000	1,400,000	810,000	1,000	6,000	S2
834	Pelagic armourhead	Pseudopentaceros richardsoni	121	480,000	1,100,000	810,000	107	252	GO[1]
835	Thornback ray	Raja clavata	3,664	810,000	810,000	810,000	4,530	4,536	S1
836	Black drum	Pogonias cromis	3,125	230,000	1,400,000	800,000	2,268	13,608	S1
837	Jamaica weakfish	Cynoscion jamaicensis	2,698	790,000	820,000	800,000	3,292	3,436	GG
838	Triportheus angulatus	Triportheus angulatus	2,915	680,000	920,000	800,000	3,160	4,300	GF
839	Spotted seabass	Dicentrarchus punctatus	955	800,000	800,000	800,000	1,200	1,200	GG
840	Portuguese dogfish	Centroscymnus coelolepis	1,161	200,000	1,400,000	790,000	841	5,703	GF
841	Madamango sea catfish	Cathorops spixii	379	290,000	1,300,000	780,000	300	1,300	GF
842	Yellow snapper	Lutjanus argentiventris	924	410,000	1,100,000	780,000	806	2,280	GG
843	Asp	Aspius aspius	2,108	770,000	770,000	770,000	2,720	2,720	S1
844	Dogfishes and hounds nei	Squalidae, Scyliorhinidae	1,133	200,000	1,300,000	770,000	840	5,713	GF
845	Cape elephantfish	Callorhinchus capensis	658	770,000	770,000	770,000	852	852	S1
846	Mud sole	Austroglossus pectoralis	425	120,000	1,400,000	770,000	300	3,500	GF
847	Knifefishes	Notopterus spp	310	740,000	790,000	770,000	390	417	GO
848	Pacific harvestfish	Peprilus medius	95	320,000	1,200,000	760,000	80	300	S2
849	Jaguar guapote	Cichlasoma managuense	251	540,000	960,000	750,000	262	461	GF
850	Bigtooth corvina	Isopisthus parvipinnis	63	520,000	980,000	750,000	64	122	GF
851	Sea chubs nei	Kyphosidae	95	410,000	1,000,000	730,000	90	231	GO
852	Bighead carp	Hypophthalmichthys nobilis	1,897	730,000	730,000	730,000	2,611	2,611	S3
853	Anisotremus scapularis	Anisotremus scapularis	110	480,000	960,000	720,000	114	228	GF
854	Yellow-edged lyretail	Variola louti	958	330,000	1,100,000	720,000	866	2,900	GF
855	Cardinal fishes nei	Epigonus spp	1,227	450,000	970,000	710,000	1,260	2,700	GG
856	Doraops zuloagai	Doraops zuloagai	405	700,000	730,000	710,000	556	578	GO
857	Sand sole	Solea lascaris	391	110,000	1,300,000	710,000	300	3,500	GG
858	Punctuated snake-eel	Ophichthus remiger	615	210,000	1,200,000	700,000	516	2,983	GO
859	Weeverfishes nei	Trachinidae	159	690,000	690,000	690,000	230	230	GF
860	Maracaibo leatherjacket	Oligoplites palometa	59	340,000	1,000,000	690,000	56	174	GF
861	Snubnose pompano	Trachinotus blochii	64	340,000	1,000,000	680,000	63	188	GF
862	Thickback soles nei	Microchirus spp	366	100,000	1,200,000	660,000	300	3,500	GF
863	Coral hind	Cephalopholis miniata	300	300,000	1,000,000	650,000	300	1,000	GG
864	Atlantic salmon	Salmo salar	2,906	650,000	650,000	650,000	4,500	4,500	S1
865	Blue-barred parrotfish	Scarus ghobban	81	360,000	920,000	640,000	88	224	GO
866	Loricaria cataphracta	Loricaria cataphracta	333	630,000	650,000	640,000	510	529	GO
867	Red scorpionfish	Scorpaena scrofa	237	570,000	700,000	640,000	338	412	GF
868	Geelbek croaker	Atractoscion aequidens	370	34,000	1,200,000	630,000	300	11,000	S2
869	Pirapatinga	Piaractus brachypomus	2,295	530,000	730,000	630,000	3,160	4,300	GF
870	Mirror dory	Zenopsis nebulosus	656	440,000	820,000	630,000	800	1,500	GF
871	Silver gemfish	Rexea solandri	1,251	420,000	830,000	630,000	1,500	3,000	GF
872	Masu(=Cherry) salmon	Oncorhynchus masou	1,379	550,000	690,000	620,000	2,000	2,500	S1
873	Bar jack	Caranx ruber	373	410,000	820,000	620,000	454	909	S1
874	Leafscale gulper shark	Centrophorus squamosus	900	160,000	1,100,000	610,000	841	5,707	GF
875	American eel	Anguilla rostrata	836	610,000	610,000	610,000	1,364	1,364	S1
876	Black goby	Gobius niger	12	610,000	610,000	610,000	19	19	S1
877	Pseudocurimata boulengeri	Pseudocurimata boulengeri	35	600,000	600,000	600,000	57	58	GO
878	Tropical gar	Atractosteus tropicus	28	400,000	800,000	600,000	35	70	GC
879	Southern stingray	Dasyatis americana	1,891	370,000	830,000	600,000	2,277	5,128	GO
880	Blackspotted rubberlip	Plectorhinchus gaterinus	320	400,000	800,000	600,000	400	800	GG
881	Redtail catfish	Phractocephalus hemioliopterus	507	560,000	630,000	600,000	800	905	GF
882	Kitefin shark	Dalatias licha	872	150,000	1,000,000	590,000	843	5,679	GF
883	Largeeye breams	Gymnocranius spp	200	430,000	750,000	590,000	268	463	GO[1]
884	Mediterranean sand smelt	Atherina hepsetus	7	570,000	570,000	570,000	12	12	S2
885	Dusky grouper	Epinephelus marginatus	1,767	460,000	650,000	550,000	2,720	3,840	S1
886	Pond loach	Misgurnus anguillicaudatus	26	440,000	670,000	550,000	39	60	GO
887	Grayling	Thymallus thymallus	249	280,000	830,000	550,000	300	900	S1
888	Meagre	Argyrosomus regius	5,659	350,000	750,000	550,000	7,561	16,025	GG
889	Duskytail grouper	Epinephelus bleekeri	1,804	260,000	840,000	550,000	2,159	7,012	GG
890	Tope shark	Galeorhinus galeus	4,593	170,000	920,000	540,000	5,000	27,000	S1
891	Daggerhead breams nei	Chrysoblephus spp	213	450,000	630,000	540,000	336	474	GF
892	Yellowfin hind	Cephalopholis hemistiktos	247	250,000	820,000	530,000	300	1,000	GG
893	Wahoo	Acanthocybium solandri	4,338	110,000	960,000	530,000	4,536	38,636	S1
894	Alexandria pompano	Alectis alexandrinus	970	530,000	530,000	530,000	1,816	1,816	S3
895	Malabar blood snapper	Lutjanus malabaricus	881	520,000	540,000	530,000	1,622	1,702	S2
896	Brown meagre	Sciaena umbra	196	460,000	590,000	520,000	335	424	S1
897	Crucifix sea catfish	Arius proops	255	200,000	850,000	520,000	300	1,300	GF
898	Velvet whalefish	Barbourisia rufa	24	350,000	690,000	520,000	35	68	GC
899	Gag	Mycteroperca microlepis	856	94,000	940,000	520,000	909	9,091	S1
900	Blue fathead	Cubiceps caeruleus	27	440,000	530,000	490,000	51	61	GO
901	Southeast Atlantic soles nei	Austroglossus spp	282	81,000	940,000	510,000	300	3,500	GF
902	Sand smelt	Atherina presbyter	1	150,000	870,000	510,000	1	6	GG
903	Chubs nei	Leuciscus spp	17	400,000	620,000	510,000	27	42	GF
904	Blackfin tuna	Thunnus atlanticus	2,207	320,000	690,000	500,000	3,182	7,000	S1
905	Latchet(=Sharpbeak gurnard)	Pterygotrigla polyommata	224	440,000	570,000	500,000	392	512	GF
906	Mango tilapia	Sarotherodon galilaeus	163	370,000	640,000	500,000	255	442	GF
907	Dorado	Salminus brasiliensis	1,831	430,000	580,000	500,000	3,160	4,300	GF
908	Smooth-hound	Mustelus mustelus	1,052	250,000	740,000	490,000	1,420	4,260	S2
909	Pacific tripletail	Lobotes pacificus	61	280,000	710,000	490,000	87	219	GO
910	Pacific salmons nei	Oncorhynchus spp	1,108	360,000	620,000	490,000	1,776	3,080	GG
911	Spotted estuary smooth-hound	Mustelus lenticulatus	1,388	350,000	630,000	490,000	2,203	3,933	GG
912	Yellowfin river pellona	Pellona flavipinnis	196	330,000	650,000	490,000	300	600	GF
913	Yellowfin seabream	Acanthopagrus latus	599	490,000	490,000	490,000	1,228	1,228	GG
914	Sheepshead	Archosargus probatocephalus	818	360,000	600,000	480,000	1,361	2,268	S1
915	Broad-barred king mackerel	Scomberomorus semifasciatus	363	180,000	750,000	470,000	483	2,013	GG
916	Largescaled terapon	Terapon theraps	54	260,000	670,000	460,000	81	206	GO
917	Tomtate grunt	Haemulon aurolineatum	69	310,000	610,000	460,000	113	226	GF
918	King dory	Cyttus traversi	480	320,000	600,000	460,000	800	1,500	GF
919	Blackfin goosefish	Lophius gastrophysus	3,016	370,000	550,000	460,000	5,514	8,090	GG
920	Pacific flatiron herring	Harengula thrissina	17	390,000	520,000	450,000	32	43	GF
921	New Zealand rough skate	Zearaja nasuta	1,565	300,000	600,000	450,000	2,598	5,277	GF
922	Ruffe	Gymnocephalus cernuus	401	310,000	570,000	440,000	703	1,293	GF
923	Citharids nei	Citharidae	343	270,000	590,000	430,000	579	1,271	GO
924	Raphael catfish	Platydoras costatus	208	420,000	440,000	430,000	478	495	GO
925	Suckerfishes, remoras nei	Echeneidae	54	240,000	620,000	430,000	88	230	GO
926	Spotted ray	Raja montagui	1,490	280,000	560,000	420,000	2,658	5,352	GG
927	Lingcod	Ophiodon elongatus	2,371	170,000	660,000	420,000	3,600	13,608	S1
928	Marbled swamp eel	Synbranchus marmoratus	19	280,000	560,000	420,000	35	70	GC
929	Snake mackerels, escolars nei	Gempylidae	831	280,000	550,000	420,000	1,500	3,000	GF
930	Pacific drum	Larimus pacificus	37	290,000	540,000	420,000	69	128	GF
931	Yellowtail amberjack	Seriola lalandi	1,553	310,000	520,000	410,000	3,000	5,000	S1

932	Striped bass	Morone saxatilis	2,902	180,000	640,000	410,000	4,536	15,900	S1
933	Two-spot red snapper	Lutjanus bohar	369	74,000	740,000	410,000	500	5,000	S2
934	Hemiodus spp	Hemiodus spp	17	400,000	400,000	400,000	42	42	GO
935	Bluenose warehou	Hyperoglyphe antarctica	2,243	400,000	400,000	400,000	5,670	5,670	S1
936	Great Northern tilefish	Lopholatilus chamaeleonticeps	1,128	230,000	560,000	390,000	2,000	5,000	S1
937	Smalltooth emperor	Lethrinus microdon	147	300,000	480,000	390,000	306	485	GO[1]
938	Blackhead seabream	Acanthopagrus schlegeli	505	390,000	390,000	390,000	1,297	1,297	S2
939	Hemiramphus spp	Hemiramphus spp	31	160,000	610,000	380,000	51	197	GG
940	Lophiosilurus alexandri	Lophiosilurus alexandri	323	360,000	400,000	380,000	800	905	GF
941	White croaker	Genyonemus lineatus	33	260,000	500,000	380,000	66	124	GF
942	Darkblotched rockfish	Sebastes crameri	135	340,000	410,000	380,000	325	393	GG
943	Pacific spadefish	Chaetodipterus zonatus	18	250,000	510,000	380,000	36	73	GC
944	Butter catfish	Ompok bimaculatus	1,412	210,000	540,000	370,000	2,624	6,700	GF
945	American shad	Alosa sapidissima	483	210,000	530,000	370,000	907	2,272	S1
946	Turbots nei	Scophthalmidae	644	300,000	440,000	370,000	1,459	2,162	GF
947	Ruffs, barrelfishes nei	Centrolophidae	772	210,000	530,000	370,000	1,464	3,665	GF
948	Rabbit fish	Chimaera monstrosa	298	350,000	350,000	350,000	852	852	GO
949	Slender tuna	Allothunnus fallai	188	230,000	460,000	340,000	408	816	GF
950	Shortfin mako	Isurus oxyrinchus	10,783	340,000	340,000	340,000	31,300	31,300	S1
951	Thicklip grey mullet	Chelon labrosus	383	260,000	430,000	340,000	892	1,489	GF
952	Needlescaled queenfish	Scomberoides tol	261	160,000	520,000	340,000	499	1,650	GG
953	Minstrel sweetlips	Plectorhinchus schotaf	180	230,000	450,000	340,000	400	800	GG
954	Pacora	Plagioscion surinamensis	30	230,000	430,000	330,000	68	128	GF
955	Humpback red snapper	Lutjanus gibbus	396	170,000	490,000	330,000	808	2,277	GG
956	Pacific bluefin tuna	Thunnus orientalis	18,198	180,000	480,000	330,000	38,000	99,300	S1
957	Grey snapper	Lutjanus griseus	398	180,000	480,000	330,000	823	2,250	GG
958	Whip stingray	Dasyatis akajei	1,710	270,000	380,000	320,000	4,495	6,415	GO
959	Sobaity seabream	Sparidentex hasta	127	270,000	370,000	320,000	339	477	GF
960	Banded jewelfish	Hemichromis fasciatus	108	230,000	410,000	320,000	264	464	GF
961	Minor stardrum	Stellifer minor	29	220,000	420,000	320,000	69	128	GF
962	Rocklings nei	Gaidropsarus spp	98	140,000	490,000	320,000	199	684	GF
963	Thinlip grey mullet	Liza ramada	347	230,000	410,000	320,000	847	1,534	GG
964	Lake trout(=Char)	Salvelinus namaycush	938	320,000	320,000	320,000	2,948	2,948	S1
965	Escolar	Lepidocybium flavobrunneum	629	210,000	420,000	310,000	1,500	3,000	GF
966	Hemibates stenosoma	Hemibates stenosoma	101	230,000	390,000	310,000	255	435	GF
967	Tiger sorubim	Pseudoplatystoma tigrinum	264	290,000	330,000	310,000	800	905	GF
968	Starry ray	Raja radiata	1,104	200,000	410,000	310,000	2,719	5,391	GG
969	Santer seabream	Cheimerius nufar	119	250,000	360,000	310,000	334	469	GF
970	Balao halfbeak	Hemiramphus balao	40	300,000	300,000	300,000	133	133	S1
971	Ballan wrasse	Labrus bergylta	200	100,000	500,000	300,000	401	2,000	S1
972	Hoplias microlepis	Hoplias microlepis	22	300,000	300,000	300,000	75	75	GO
973	Highwaterman catfish	Hypophthalmus edentatus	246	270,000	310,000	290,000	800	905	GF
974	Squaretail coralgrouper	Plectropomus areolatus	373	130,000	440,000	280,000	852	2,860	GF
975	Frostfishes	Benthodesmus spp	64	240,000	330,000	280,000	198	268	GF
976	Blackmouth croaker	Atrobucca nibe	442	260,000	290,000	280,000	1,500	1,700	S2
977	Barbus cyclolepis	Barbus cyclolepis	1	29,000	520,000	280,000	2	38	S2
978	White weakfish	Atractoscion nobilis	161	15,000	540,000	280,000	300	11,000	GG
979	Hammerhead sharks, etc. nei	Sphyrnidae	5,423	54,000	490,000	270,000	11,000	100,000	GF
980	Smooth weakfish	Cynoscion leiarchus	904	270,000	280,000	270,000	3,248	3,401	GG
981	Curled picarel	Centracanthus cirrus	11	240,000	300,000	270,000	35	44	GF
982	Squirrelfishes nei	Holocentridae	308	220,000	310,000	270,000	980	1,403	GO
983	Trouts nei	Salmo spp	750	190,000	350,000	270,000	2,163	4,044	GG
984	Greater amberjack	Seriola dumerili	2,995	170,000	370,000	270,000	8,182	18,144	S1
985	Marini's anchovy	Anchoa marinii	3	260,000	260,000	260,000	10	10	GG
986	Roving coralgrouper	Plectropomus pessuliferus	341	120,000	400,000	260,000	843	2,829	GF
987	Five-lined snapper	Lutjanus quinquelineatus	315	140,000	390,000	260,000	818	2,261	GG
988	Hapuku wreckfish	Polyprion oxygeneios	1,541	260,000	260,000	260,000	5,897	5,897	S1
989	White stumpnose	Rhabdosargus globiceps	104	210,000	300,000	260,000	348	487	GF
990	Bigeye trevally	Caranx sexfasciatus	121	92,000	420,000	260,000	287	1,312	GG
991	Bonnetmouths, rubyfishes nei	Emmelichthyidae	22	72,000	430,000	250,000	50	300	GF
992	Draughtsboard shark	Cephaloscyllium isabellum	43	250,000	250,000	250,000	172	172	GF
993	Corkwing wrasse	Symphodus melops	176	83,000	410,000	240,000	432	2,117	GF
994	Silky shark	Carcharhinus falciformis	7,660	200,000	290,000	240,000	26,680	37,899	GG
995	Spotted gurnard	Pterygotrigla picta	108	210,000	280,000	240,000	393	509	GF
996	Painted sweetlips	Diagramma pictum	37	160,000	330,000	240,000	113	226	GF
997	Caquetaia kraussii	Caquetaia kraussii	71	190,000	300,000	240,000	235	378	GF
998	Cusk-eels, brotulas nei	Ophidiidae	939	230,000	250,000	240,000	3,735	4,032	GF
999	Green weakfish	Cynoscion virescens	810	240,000	250,000	240,000	3,291	3,430	GG
1000	Humpnose big-eye bream	Monotaxis grandoculis	66	160,000	320,000	240,000	206	418	GO[1]
1001	Arctic char	Salvelinus alpinus	198	44,000	440,000	240,000	454	4,545	S1
1002	Chilipepper rockfish	Sebastes goodei	216	180,000	300,000	240,000	720	1,200	S1
1003	Biglip grunt	Plectorhinchus macrolepis	128	160,000	320,000	240,000	400	800	GG
1004	Bobo mullet	Joturus pichardi	270	240,000	240,000	240,000	1,131	1,131	S2
1005	Argentine angelshark	Squatina argentina	3,747	210,000	270,000	240,000	14,000	18,000	GG
1006	Sawsharks nei	Pristiophorus spp	359	62,000	410,000	240,000	868	5,824	GO
1007	Banded astyanax	Astyanax fasciatus	859	200,000	270,000	240,000	3,160	4,300	GF
1008	Marlins,sailfishes,etc. nei	Istiophoridae	9,803	170,000	300,000	240,000	32,948	56,804	GF
1009	Boxfishes nei	Ostraciidae	111	230,000	240,000	230,000	462	484	GO
1010	Southern lemon sole	Pelotretis flavilatus	187	160,000	310,000	230,000	598	1,199	GF
1011	Atlantic sabretooth anchovy	Lycengraulis grossidens	5	230,000	230,000	230,000	19	19	S2
1012	Longspine thornyhead	Sebastolobus altivelis	87	210,000	260,000	230,000	339	416	GF
1013	Black rockfish	Sebastes melanops	178	150,000	310,000	230,000	564	1,166	S1
1014	Sand flounders nei	Rhombosolea spp	186	150,000	310,000	230,000	598	1,201	GF
1015	Leaping mullet	Liza saliens	54	200,000	260,000	230,000	209	270	S1
1016	Snakehead	Channa argus	46	220,000	230,000	230,000	200	205	GG
1017	Gafftopsail sea catfish	Bagre marinus	108	83,000	360,000	220,000	300	1,300	GF
1018	Cubera snapper	Lutjanus cyanopterus	266	120,000	320,000	220,000	830	2,236	GG
1019	Black scorpionfish	Scorpaena porcus	83	200,000	240,000	220,000	342	419	GF
1020	Blackgill rockfish	Sebastes melanostomus	74	200,000	230,000	220,000	317	377	GG
1021	Lusitanian cownose ray	Rhinoptera marginata	671	130,000	300,000	210,000	2,261	5,108	GO
1022	Silvery John dory	Zenopsis conchifer	221	150,000	280,000	210,000	800	1,500	GF
1023	Doublespotted queenfish	Scomberoides lysan	161	98,000	320,000	210,000	499	1,650	GG
1024	Sculpins nei	Cottidae	327	180,000	240,000	210,000	1,361	1,814	GF
1025	Bonefish	Albula vulpes	691	210,000	210,000	210,000	3,333	3,333	S2
1026	Flatwhiskered catfish	Pinirampus pirinampu	176	190,000	220,000	210,000	800	905	GF
1027	Guachanche barracuda	Sphyraena guachancho	325	39,000	370,000	210,000	874	8,294	GG
1028	Atlantic tomcod	Microgadus tomcod	18	200,000	200,000	200,000	90	90	S2
1029	Blue antimora	Antimora rostrata	134	94,000	310,000	200,000	438	1,434	S1
1030	Atlantic flyingfish	Cheilopogon melanurus	17	99,000	300,000	200,000	56	169	GO
1031	Blue marlin	Makaira nigricans	35,586	200,000	200,000	200,000	180,000	180,000	S1
1032	Dusky smooth-hound	Mustelus canis	729	200,000	200,000	200,000	3,719	3,719	S1
1033	Mexican barred snapper	Hoplopagrus guentherii	253	150,000	240,000	200,000	1,064	1,647	GF
1034	Coney	Cephalopholis fulva	90	90,000	300,000	200,000	300	1,000	GG
1035	Hickory shad	Alosa mediocris	53	180,000	210,000	200,000	247	301	GG

1036	Shi drum	Umbrina cirrosa	153	190,000	200,000	190,000	765	820	GG
1037	Silver grunter	Mesopristes argenteus	23	110,000	280,000	190,000	84	212	GO
1038	African striped grunt	Parapristipoma octolineatum	30	130,000	260,000	190,000	117	234	GF
1039	Splendid alfonsino	Beryx splendens	235	190,000	190,000	190,000	1,260	1,260	S1
1040	Karanteen seabream	Crenidens crenidens	76	150,000	220,000	190,000	353	492	GF
1041	Black cusk-eel	Genypterus maculatus	830	180,000	180,000	180,000	4,536	4,536	S1
1042	Bastard grunt	Pomadasys incisus	27	180,000	180,000	180,000	149	149	S2
1043	Mexican barracuda	Sphyraena ensis	287	34,000	330,000	180,000	879	8,409	GG
1044	Pemarco blackfish	Schedophilus pemarco	384	100,000	260,000	180,000	1,504	3,711	GF
1045	Snake eels nei	Ophichthidae	158	53,000	300,000	180,000	523	2,955	GO
1046	New Zealand smooth skate	Dipturus innominatus	631	120,000	240,000	180,000	2,682	5,338	GF
1047	Atlantic rubyfish	Erythrocles monodi	15	50,000	300,000	180,000	50	300	GF
1048	Asian bonytongue	Scleropages formosus	54	180,000	180,000	180,000	305	308	GF
1049	Offshore rockfish	Pontinus kuhlii	61	160,000	190,000	170,000	323	385	GF
1050	Salema butterfish	Peprilus snyderi	22	73,000	270,000	170,000	80	300	GG
1051	Pati	Luciopimelodus pati	147	160,000	180,000	170,000	800	905	GF
1052	Duckbill catfish	Sorubim lima	146	160,000	180,000	170,000	800	905	GF
1053	Sordid rubberlip	Plectorhinchus sordidus	92	110,000	230,000	170,000	400	800	GG
1054	Black marlin	Makaira indica	14,971	170,000	180,000	170,000	85,000	90,000	S1
1055	Dogtooth tuna	Gymnosarda unicolor	1,569	78,000	260,000	170,000	6,000	20,000	S1
1056	European barracuda	Sphyraena sphyraena	267	32,000	310,000	170,000	872	8,242	GG
1057	Starry butterfish	Stromateus stellatus	33	130,000	200,000	170,000	166	259	GF
1058	Atlantic sailfish	Istiophorus albicans	3,568	130,000	200,000	160,000	18,140	27,220	S1
1059	White-blotched grouper	Epinephelus multinotatus	530	70,000	250,000	160,000	2,152	7,580	GG
1060	White perch	Morone americana	1,113	70,000	250,000	160,000	4,536	15,900	GG
1061	Mediterranean scaldfish	Arnoglossus laterna	126	96,000	220,000	160,000	578	1,312	GO
1062	Rusty jobfish	Aphareus rutilans	202	120,000	190,000	160,000	1,061	1,640	GF
1063	Comber	Serranus cabrilla	213	71,000	240,000	160,000	886	2,986	GF
1064	Goldsinny-wrasse	Ctenolabrus rupestris	111	52,000	250,000	150,000	436	2,133	GF
1065	Black curbinata	Plagioscion auratus	14	110,000	200,000	150,000	69	129	GF
1066	Atlantic needlefish	Strongylura marina	8	150,000	150,000	150,000	51	51	GF
1067	Senegal jack	Caranx senegallus	66	51,000	250,000	150,000	266	1,284	GG
1068	Smooth puffer	Lagocephalus laevigatus	31	130,000	160,000	150,000	195	233	GO
1069	Crested bellowfish	Notopogon lilliei	7	97,000	200,000	150,000	34	70	GC
1070	Bank rockfish	Sebastes rufus	50	130,000	160,000	150,000	313	372	GG
1071	Roughsnout grenadier	Trachyrincus scabrus	76	100,000	180,000	140,000	420	731	GF
1072	Cusk-eels nei	Genypterus spp	633	140,000	140,000	140,000	4,536	4,536	M1
1073	Limnotilapia dardennii	Limnotilapia dardennii	44	100,000	170,000	140,000	256	436	GF
1074	Chimaeras, etc. nei	Chimaeriformes	113	130,000	130,000	130,000	852	852	GO
1075	White bass	Morone chrysops	937	59,000	210,000	130,000	4,536	15,900	GG
1076	Yellowlip emperor	Lethrinus xanthochilus	38	89,000	180,000	130,000	214	426	GO[1]
1077	Twaite shad	Alosa fallax	35	120,000	140,000	130,000	245	297	GG
1078	Mountain mullet	Agonostomus monticola	17	130,000	130,000	130,000	128	128	S1
1079	Areolate grouper	Epinephelus areolatus	435	59,000	200,000	130,000	2,153	7,428	GG
1080	Mutton snapper	Lutjanus analis	154	68,000	190,000	130,000	821	2,254	GG
1081	Marbled lungfish	Protopterus aethiopicus	345	97,000	160,000	130,000	2,190	3,540	S1
1082	Tautog	Tautoga onitis	134	47,000	210,000	130,000	648	2,837	GF
1083	Snakehead kingcroaker	Menticirrhus ophicephalus	27	58,000	200,000	130,000	136	454	GG
1084	Patchwork lampfishes	Notoscopelus spp	6	85,000	170,000	130,000	38	76	GC
1085	Common dace	Leuciscus leuciscus	3	97,000	150,000	130,000	20	32	GF
1086	Marbled spinefoot	Siganus rivulatus	6	84,000	170,000	130,000	38	74	GC
1087	Yellowedge grouper	Epinephelus flavolimbatus	375	83,000	170,000	120,000	2,270	4,540	S1
1088	Splitnose rockfish	Sebastes diploproa	45	110,000	140,000	120,000	331	403	GG
1089	Gulper shark	Centrophorus granulosus	180	32,000	210,000	120,000	842	5,696	GF
1090	Silver mylossoma	Mylossoma duriventre	447	100,000	140,000	120,000	3,160	4,300	GF
1091	Spotted flounder	Citharus linguatula	99	75,000	170,000	120,000	581	1,316	GO
1092	Snowtrouts nei	Schizothorax spp	3	95,000	150,000	120,000	21	33	GF
1093	Striped marlin	Tetrapturus audax	9,239	81,000	160,000	120,000	56,750	113,500	S1
1094	Flat needlefish	Ablennes hians	53	120,000	120,000	120,000	437	437	S1
1095	Scamp	Mycteroperca phenax	217	23,000	220,000	120,000	991	9,413	GG
1096	Pinfish	Lagodon rhomboides	48	100,000	140,000	120,000	337	474	GF
1097	Antarctic toothfish	Dissostichus mawsoni	3,377	120,000	120,000	120,000	28,000	28,000	S1
1098	Arius spp	Arius spp	59	45,000	200,000	120,000	300	1,300	GF
1099	Flying gurnard	Dactylopterus volitans	35	120,000	120,000	120,000	296	296	S1
1100	Streaked gurnard	Chelidonichthys lastoviza	71	92,000	150,000	120,000	488	781	GG
1101	S.Am. freshwater stingrays nei	Potamotrygon spp	371	73,000	160,000	120,000	2,255	5,117	GO
1102	Yellownose skate	Zearaja chilensis	365	73,000	160,000	120,000	2,221	5,000	GF
1103	Milk shark	Rhizoprionodon acutus	1,821	110,000	130,000	120,000	14,417	16,625	GF
1104	Red cusk-eel	Genypterus chilensis	531	120,000	120,000	120,000	4,536	4,536	S1
1105	Green jack	Caranx caballus	53	42,000	190,000	110,000	282	1,277	GG
1106	Pigfish	Orthopristis chrysoptera	17	76,000	150,000	110,000	114	227	GF
1107	Thumbprint emperor	Lethrinus harak	40	84,000	140,000	110,000	283	473	GO[1]
1108	Peruvian chromis	Chromis intercrusma	14	64,000	160,000	110,000	88	222	GO
1109	Short-finned eel	Anguilla australis	91	91,000	130,000	110,000	685	1,002	GG
1110	Fourlined terapon	Pelates quadrilineatus	15	64,000	160,000	110,000	93	231	GO
1111	Spotfin flathead	Grammoplites suppositus	41	110,000	110,000	110,000	384	384	GF
1112	Leopard flounder	Bothus pantherinus	83	65,000	140,000	100,000	583	1,285	GO
1113	Peacock hind	Cephalopholis argus	48	48,000	160,000	100,000	300	1,000	GG
1114	Smallscaled grouper	Epinephelus polylepis	347	46,000	160,000	100,000	2,152	7,501	GG
1115	Lowfin gulper shark	Centrophorus lusitanicus	150	26,000	180,000	100,000	840	5,713	GF
1116	Chili sea catfish	Notarius troschelii	50	38,000	170,000	100,000	300	1,300	GF
1117	Brown bullhead	Ameiurus nebulosus	51	100,000	100,000	100,000	500	500	S1
1118	Pomadasys spp	Pomadasys spp	15	100,000	100,000	100,000	149	149	GG
1119	Atlantic bluefin tuna	Thunnus thynnus	25,918	99,000	99,000	99,000	262,000	262,000	S1
1120	Bathybates minor	Bathybates minor	32	73,000	120,000	99,000	256	440	GF
1121	Silk snapper	Lutjanus vivanus	118	52,000	150,000	98,000	810	2,275	GG
1122	Round whitefish	Prosopium cylindraceum	15	53,000	140,000	98,000	107	285	GF
1123	Angelfishes nei	Pomacanthidae	12	55,000	140,000	98,000	88	225	GO
1124	Pimelodus spp	Pimelodus spp	83	91,000	100,000	97,000	800	905	GF
1125	California flounder	Paralichthys californicus	285	90,000	100,000	97,000	2,722	3,175	S1
1126	Grey tilefish	Caulolatilus microps	132	84,000	110,000	96,000	1,231	1,563	GF[1]
1127	Chilean grenadier	Coelorinchus chilensis	50	69,000	120,000	94,000	420	730	GF
1128	Giant cichlid	Boulengerochromis microlepis	30	69,000	120,000	93,000	256	436	GF
1129	Pearly razorfish	Xyrichtys novacula	82	33,000	150,000	92,000	540	2,499	GF
1130	Senegalese sole	Solea senegalensis	51	14,000	170,000	92,000	300	3,500	GG
1131	Longfin halfbeak	Hemiramphus saltator	7	37,000	140,000	91,000	51	198	GG
1132	Pacific porgy	Calamus brachysomus	32	77,000	100,000	90,000	308	411	GF
1133	Blueback shad	Alosa aestivalis	18	89,000	89,000	89,000	198	198	S1
1134	Silver-rag driftfish	Ariomma bondi	5	77,000	92,000	84,000	49	59	GO
1135	Obtuse barracuda	Sphyraena obtusata	138	17,000	160,000	88,000	867	8,146	GG
1136	Birdbeak dogfish	Deania calcea	229	87,000	87,000	87,000	2,612	2,612	S2
1137	Chilhuil sea catfish	Bagre panamensis	43	33,000	140,000	87,000	300	1,300	GF
1138	Aplodactylus punctatus	Aplodactylus punctatus	11	49,000	120,000	85,000	89	218	GO
1139	Pacific sanddab	Citharichthys sordidus	113	54,000	120,000	85,000	977	2,101	GF

1140	Hagfish	Myxine glutinosa	4	57,000	110,000	84,000	40	78	GA
1141	Raja rays nei	Raja spp	247	49,000	120,000	84,000	2,092	5,027	GG
1142	South American silver porgy	Diplodus argenteus	33	69,000	98,000	83,000	336	473	GF
1143	Pacific sand sole	Psettichthys melanostictus	66	54,000	110,000	81,000	613	1,222	GF
1144	Northern puffer	Sphoeroides maculatus	38	78,000	84,000	81,000	452	484	GO
1145	Flathead catfish	Pylodictis olivaris	119	74,000	88,000	81,000	1,350	1,597	GF
1146	Lanternsharks nei	Etmopterus spp	118	21,000	140,000	81,000	844	5,668	GF
1147	Barbel	Barbus barbus	192	53,000	110,000	80,000	1,800	3,636	S1
1148	Reticulate knifefish	Papyrocranus afer	34	75,000	82,000	79,000	419	456	GO
1149	Redtail parrotfish	Sparisoma chrysopterum	10	44,000	110,000	77,000	89	223	GO
1150	Shortbelly rockfish	Sebastes jordani	28	67,000	83,000	75,000	333	412	GG
1151	Mastacembelus cunningtoni	Mastacembelus cunningtoni	3	49,000	100,000	75,000	35	70	GC
1152	Danube bleak	Chalcalburnus chalcoides	2	56,000	89,000	72,000	26	41	GF
1153	Gopher rockfish	Sebastes carnatus	26	66,000	79,000	72,000	323	389	GG
1154	Auchenoglanis biscutatus	Auchenoglanis biscutatus	60	72,000	72,000	72,000	830	830	GF
1155	Odontesthes smitti	Odontesthes smitti	0.2	23,000	120,000	72,000	1	7	GF
1156	Channel bull blenny	Cottoperca gobio	9	42,000	100,000	72,000	90	216	GO
1157	Sharpsnout seabream	Diplodus puntazzo	26	61,000	82,000	71,000	315	429	GF
1158	Panama hake	Merluccius angustimanus	36	57,000	92,000	74,000	387	629	GG
1159	Sandy ray	Raja circularis	263	48,000	92,000	70,000	2,847	5,487	GG
1160	Sabre squirrelfish	Sargocentron spiniferum	81	59,000	81,000	70,000	988	1,373	GO
1161	Vermilion rockfish	Sebastes miniatus	26	63,000	77,000	70,000	332	405	GG
1162	Green terror	Aequidens rivulatus	22	51,000	87,000	69,000	252	433	GF
1163	Red sea catfish	Bagre pinnimaculatus	33	26,000	110,000	68,000	300	1,300	GF
1164	Thresher sharks nei	Alopias spp	8,942	66,000	70,000	68,000	127,411	135,449	GG
1165	Unicorn icefish	Channichthys rhinoceratus	51	58,000	78,000	68,000	649	876	GO[1]
1166	Chars nei	Salvelinus spp	148	48,000	87,000	68,000	1,697	3,080	GG
1167	Gorean snapper	Lutjanus goreensis	81	37,000	95,000	66,000	853	2,196	GG
1168	Dungat grouper	Epinephelus goreensis	214	30,000	99,000	65,000	2,154	7,215	GG
1169	Starry flounder	Platichthys stellatus	51	26,000	100,000	64,000	500	2,000	GG
1170	Red hind	Epinephelus guttatus	212	28,000	99,000	63,000	2,149	7,578	GG
1171	Whitespotted wedgefish	Rhynchobatus australiae	4,901	61,000	61,000	61,000	80,000	80,000	GG
1172	Electric catfish	Malapterurus electricus	33	60,000	62,000	61,000	534	555	GO
1173	Dories nei	Zeidae	63	42,000	79,000	61,000	800	1,500	GF
1174	Queen snapper	Etelis oculatus	77	48,000	72,000	60,000	1,072	1,619	GF
1175	Mediterranean moray	Muraena helena	53	18,000	100,000	60,000	517	2,985	GO
1176	Longnose velvet dogfish	Centroscymnus crepidater	87	15,000	100,000	59,000	843	5,682	GF
1177	Shagreen ray	Raja fullonica	191	37,000	80,000	59,000	2,373	5,138	GG
1178	Sauger	Sander canadensis	94	44,000	73,000	58,000	1,283	2,147	GG
1179	Bluefin trevally	Caranx melampygus	28	21,000	94,000	57,000	297	1,331	GG
1180	Golden African snapper	Lutjanus fulgens	71	32,000	82,000	57,000	859	2,189	GG
1181	Whitson's grenadier	Macrourus whitsoni	65	45,000	68,000	56,000	952	1,447	GF[1]
1182	Pacific angelshark	Squatina californica	881	49,000	63,000	56,000	14,000	18,000	GG
1183	Peruvian sea catfish	Galeichthys peruvianus	27	21,000	91,000	56,000	300	1,300	GF
1184	Permit	Trachinotus falcatus	6	27,000	83,000	55,000	68	209	GF
1185	Righteye flounders nei	Pleuronectidae	45	37,000	73,000	55,000	624	1,235	GF
1186	Red drum	Sciaenops ocellatus	87	24,000	85,000	55,000	1,021	3,636	S1
1187	Pimelodus maculatus	Pimelodus maculatus	46	51,000	58,000	54,000	800	905	GF
1188	Mud carp	Cirrhinus molitorella	3	43,000	65,000	54,000	39	59	GF
1189	Hypostomus spp	Hypostomus spp	31	52,000	54,000	53,000	579	603	GO
1190	Longnose spurdog	Squalus blainville	77	13,000	92,000	52,000	835	5,781	GG
1191	Goldsilk seabream	Acanthopagrus berda	64	52,000	52,000	52,000	1,226	1,226	GG
1192	Streaked seerfish	Scomberomorus lineolatus	40	21,000	83,000	52,000	480	1,912	GG
1193	Parassi mullet	Mugil incilis	57	42,000	63,000	52,000	910	1,368	GG
1194	Longnosed skate	Raja oxyrinchus	177	34,000	70,000	52,000	2,512	5,225	GG
1195	Wuchang bream	Megalobrama amblycephala	2	40,000	64,000	52,000	24	37	GF
1196	Slimeheads nei	Trachichthyidae	71	51,000	51,000	51,000	1,400	1,400	GF
1197	African red snapper	Lutjanus agennes	64	29,000	72,000	51,000	881	2,162	GG
1198	Nassau grouper	Epinephelus striatus	227	50,000	50,000	50,000	4,540	4,540	S1
1199	Black grunt	Haemulon bonariense	7	33,000	66,000	49,000	108	217	GF
1200	Bubu	Auchenoglanis occidentalis	41	49,000	49,000	49,000	830	830	GF
1201	Zamurito	Calophysus macropterus	41	46,000	52,000	49,000	800	905	GF
1202	Patagonian blennie	Eleginops maclovinus	378	35,000	58,000	47,000	6,499	10,661	GF
1203	Kelp greenling	Hexagrammos decagrammus	25	45,000	47,000	46,000	522	542	GF
1204	Small-eyed ray	Raja microocellata	143	28,000	64,000	46,000	2,234	5,022	GG
1205	Wreckfish	Polyprion americanus	846	37,000	54,000	46,000	15,649	22,600	S1
1206	New Zealand dory	Cyttus novaezealandiae	48	32,000	59,000	46,000	800	1,500	GF
1207	Dusky spinefoot	Siganus luridus	2	30,000	60,000	45,000	38	74	GC
1208	Longneck croaker	Pseudotolithus typus	11	44,000	46,000	45,000	229	242	GG
1209	Peruvian mojarra	Diapterus peruvianus	5	25,000	63,000	44,000	87	219	GO
1210	African forktail snapper	Apsilus fuscus	55	33,000	53,000	43,000	1,052	1,664	GF
1211	Sawfishes	Pristidae	141	27,000	59,000	43,000	2,397	5,245	GO
1212	Red pike conger	Cynoponticus coniceps	36	12,000	73,000	43,000	500	3,000	GF
1213	Brotula clarki	Brotula clarki	46	34,000	51,000	42,000	909	1,364	GG
1214	Windowpane flounder	Scophthalmus aquosus	74	34,000	50,000	42,000	1,477	2,145	GG
1215	Longfin hake	Phycis chesteri	13	18,000	65,000	42,000	204	718	GF
1216	White margate	Haemulon album	6	27,000	55,000	41,000	113	226	GF
1217	Trumpet emperor	Lethrinus miniatus	18	34,000	48,000	41,000	368	515	GG
1218	Cabezon	Scorpaenichthys marmoratus	64	35,000	47,000	41,000	1,361	1,814	S1
1219	Trout sweetlips	Plectorhinchus pictus	22	27,000	55,000	41,000	400	800	GG
1220	Lantern fish	Lampanyctus achirus	2	27,000	53,000	40,000	36	71	GC
1221	Sturgeons nei	Acipenseridae	617	37,000	43,000	40,000	14,466	16,535	GF
1222	Canary rockfish	Sebastes pinniger	80	40,000	40,000	40,000	2,000	2,030	S1
1223	Silver-cheeked toadfish	Lagocephalus sceleratus	15	37,000	42,000	40,000	343	391	GO
1224	Snowy grouper	Epinephelus niveatus	160	35,000	44,000	40,000	3,636	4,545	S1
1225	Giant boarfish	Paristiopterus labiosus	11	24,000	56,000	40,000	200	471	GO[1]
1226	Peacock cichlid	Cichla ocellaris	13	29,000	50,000	39,000	256	445	GF
1227	Sand tilefish	Malacanthus plumieri	5	22,000	56,000	39,000	88	221	GO
1228	Rock cook	Centrolabrus exoletus	30	13,000	64,000	39,000	461	2,224	GF
1229	Allis shad	Alosa alosa	70	39,000	39,000	39,000	1,800	1,800	S1
1230	Knifetooth dogfish	Scymnodon ringens	57	9,900	67,000	39,000	842	5,688	GF
1231	Blue rockfish	Sebastes mystinus	13	35,000	42,000	38,000	320	384	GG
1232	Tunas nei	Thunnini	21	24,000	50,000	37,000	426	877	GF
1233	Cero	Scomberomorus regalis	112	25,000	49,000	37,000	2,268	4,536	S1
1234	Orange perch	Lepidoperca pulchella	47	17,000	57,000	37,000	834	2,822	GF
1235	Long snouted lancetfish	Alepisaurus ferox	1	32,000	40,000	36,000	28	36	GO
1236	Armed snook	Centropomus armatus	56	11,000	60,000	35,000	941	4,970	GF
1237	Small red scorpionfish	Scorpaena notata	13	32,000	39,000	35,000	337	414	GF
1238	Green jobfish	Aprion virescens	46	28,000	43,000	35,000	1,066	1,629	GF
1239	Rock bass	Ambloplites rupestris	4	20,000	50,000	35,000	83	209	GO
1240	Grey rockcod	Notothenia squamifrons	282	26,000	43,000	35,000	6,569	10,739	GF
1241	Guitarfishes, etc. nei	Rhinobatidae	2,746	34,000	34,000	34,000	80,000	80,000	GF
1242	Spiny scorpionfish	Trachyscorpia echinata	11	32,000	37,000	34,000	302	351	GF
1243	Threadfin shad	Dorosoma petenense	1	2,300	66,000	34,000	18	520	GG

1244	Greenland shark	Somniosus microcephalus	50	8,800	59,000	34,000	844	5,670	GF
1245	Prussian carp	Carassius gibelio	15	26,000	41,000	33,000	356	572	S1
1246	Cape fathead	Cubiceps capensis	2	28,000	34,000	31,000	51	62	GO
1247	True sole	Heteromycteris proboscideus	18	5,100	60,000	32,000	300	3,500	GF
1248	Blue skate	Raja batis	340	7,400	57,000	32,000	5,960	46,150	S3
1249	Pink dentex	Dentex gibbosus	10	17,000	47,000	32,000	219	610	GG
1250	Pacific crevalle jack	Caranx caninus	13	10,000	52,000	31,000	252	1,304	GG
1251	Bluespotted cornetfish	Fistularia commersonii	2	21,000	41,000	31,000	37	73	GC
1252	Spinetail mobula	Mobula japanica	95	19,000	43,000	31,000	2,243	5,132	GO
1253	Chrysichthys sianenna	Chrysichthys sianenna	25	30,000	30,000	30,000	830	830	GG
1254	Black dogfish	Centroscyllium fabricii	45	7,800	53,000	30,000	838	5,735	GF
1255	Brown sea catfish	Sciades dowii	15	11,000	49,000	30,000	300	1,300	GF
1256	Slobbering catfish	Goslinia platynema	26	28,000	32,000	30,000	800	905	GF
1257	Violet warehou	Schedophilus velaini	65	17,000	43,000	30,000	1,515	3,724	GF
1258	Tarpon	Megalops atlanticus	990	25,000	33,000	29,000	29,680	40,000	S1
1259	Pacific sandperch	Prolatilus jugularis	53	28,000	29,000	29,000	1,820	1,877	GF
1260	Scorpionfishes, rockfishes nei	Scorpaena spp	10	25,000	31,000	28,000	332	407	GF
1261	Cape redfish	Sebastes capensis	11	25,000	31,000	28,000	343	419	GG
1262	Angular roughshark	Oxynotus centrina	41	7,100	48,000	28,000	855	5,778	GO
1263	Slickheads nei	Alepocephalus spp	26	22,000	33,000	28,000	774	1,169	GG
1264	Squirrelfish	Holocentrus adscensionis	34	23,000	32,000	28,000	1,063	1,451	GO
1265	Common bluestripe snapper	Lutjanus kasmira	32	14,000	40,000	27,000	803	2,288	GG
1266	Blacktip shark	Carcharhinus limbatus	478	27,000	27,000	27,000	18,000	18,000	S1
1267	Player scorpionfish	Scorpaena histrio	10	24,000	29,000	26,000	333	410	GF
1268	Knout goby	Mesogobius batrachocephalus	1	23,000	30,000	26,000	28	35	GC[1]
1269	Mackerel sharks,porbeagles nei	Lamnidae	847	26,000	26,000	26,000	32,355	32,355	GF
1270	Longfin yellowtail	Seriola rivoliana	166	18,000	34,000	26,000	4,905	8,998	GG
1271	Bichique	Sicyopterus lagocephalus	1	26,000	26,000	26,000	19	19	GF
1272	Spotted eagle ray	Aetobatus narinari	80	16,000	35,000	25,000	2,270	5,138	GO
1273	Pimelodus albicans	Pimelodus albicans	21	24,000	27,000	25,000	800	905	GF
1274	Blackbellied angler	Lophius budegassa	114	25,000	25,000	25,000	4,536	4,536	S1
1275	Butterfly kingfish	Gasterochisma melampus	14	16,000	34,000	25,000	412	871	GF
1276	Velvet belly	Etmopterus spinax	37	6,500	44,000	25,000	845	5,652	GF
1277	Slender rockfish	Scorpaena elongata	9	22,000	27,000	24,000	337	413	GF
1278	Redmouth grouper	Aethaloperca rogaa	32	11,000	37,000	24,000	855	2,869	GF
1279	Red lionfish	Pterois volitans	9	22,000	27,000	24,000	343	422	GF
1280	Opah	Lampris guttatus	891	24,000	24,000	24,000	37,100	37,100	S1
1281	Atlantic white marlin	Tetrapturus albidus	646	24,000	24,000	24,000	27,215	27,215	S1
1282	Copper rockfish	Sebastes caurinus	8	21,000	26,000	24,000	324	392	GG
1283	Angelshark	Squatina squatina	369	21,000	26,000	23,000	14,000	18,000	S1
1284	Eelpouts	Lycodes spp	157	23,000	23,000	23,000	6,804	6,804	GO
1285	Intermediate scabbardfish	Aphanopus intermedius	10	20,000	26,000	23,000	367	474	GG
1286	Shorthorn sculpin	Myoxocephalus scorpius	35	19,000	26,000	23,000	1,361	1,814	GF
1287	Ribbonfishes	Trachipteridae	822	22,000	22,000	22,000	37,100	37,100	GO
1288	Mediterranean starry ray	Raja asterias	67	13,000	31,000	22,000	2,185	4,983	GG
1289	Blacktail comber	Serranus atricauda	29	10,000	33,000	22,000	870	2,865	GF
1290	Madeira lantern fish	Ceratoscopelus maderensis	1	15,000	29,000	22,000	38	76	GC
1291	Smooth butterfly ray	Gymnura micrura	68	13,000	30,000	21,000	2,281	5,135	GO
1292	Largescale triggerfish	Canthidermis macrolepis	10	21,000	21,000	21,000	491	491	GF
1293	Rough triggerfish	Canthidermis maculata	3	19,000	23,000	21,000	115	140	GF
1294	Moray cods nei	Muraenolepis spp	6	9,600	31,000	21,000	206	674	GO
1295	Redbanded rockfish	Sebastes babcocki	8	18,000	23,000	20,000	338	419	GG
1296	Rudderfish	Centrolophus niger	42	12,000	29,000	20,000	1,450	3,647	GF
1297	Atlantic sharpnose shark	Rhizoprionodon terraenovae	312	19,000	22,000	20,000	14,471	16,696	GF
1298	Forktail lates	Lates microlepis	31	6,200	33,000	20,000	949	5,045	GG
1299	Porthole shovelnose catfish	Hemisorubim platyrhynchos	17	18,000	21,000	20,000	800	905	GF
1300	Atlantic bigeye	Priacanthus arenatus	21	19,000	19,000	19,000	1,100	1,100	S1
1301	Smallnose fanskate	Sympterygia bonapartii	59	12,000	27,000	19,000	2,209	5,007	GF
1302	Blue bobo	Polydactylus approximans	5	19,000	19,000	19,000	269	269	GG
1303	Lookdown	Selene vomer	6	16,000	22,000	19,000	259	360	GG
1304	Houting	Coregonus oxyrinchus	3	10,000	27,000	19,000	118	309	GG
1305	Rio skate	Rioraja agassizi	57	11,000	26,000	19,000	2,217	5,015	GF
1306	Machaca	Brycon guatemalensis	67	16,000	21,000	18,000	3,160	4,300	GF
1307	Arapaima	Arapaima gigas	1,340	18,000	18,000	18,000	73,100	73,100	S3
1308	Gurnards nei	Trigla spp	8	16,000	21,000	18,000	396	522	GF
1309	Longspine burrfish	Tragulichthys jaculiferus	9	17,000	19,000	18,000	458	495	GO
1310	Argentine conger	Conger orbignyanus	165	18,000	18,000	18,000	9,076	9,100	GG
1311	Giant manta	Manta birostris	56	11,000	25,000	18,000	2,260	5,143	GO
1312	Redcoat	Sargocentron rubrum	22	15,000	21,000	18,000	1,057	1,462	GO
1313	Senegalese tonguesole	Cynoglossus senegalensis	14	11,000	25,000	18,000	579	1,351	GO
1314	China rockfish	Sebastes nebulosus	6	16,000	19,000	17,000	331	402	GG
1315	Undulate ray	Raja undulata	53	11,000	24,000	17,000	2,194	4,984	GG
1316	Tete sea catfish	Ariopsis seemanni	8	6,500	28,000	17,000	300	1,300	GF
1317	Jolthead porgy	Calamus bajonado	7	14,000	20,000	17,000	327	454	GF
1318	Chilean sandperch	Pinguipes chilensis	32	17,000	17,000	17,000	1,820	1,877	GF
1319	Blackfin snook	Centropomus medius	27	5,400	29,000	17,000	941	4,970	GF
1320	Grenadier cod	Tripterophycis gilchristi	7	8,300	25,000	17,000	290	875	GF
1321	Grunts nei	Haemulon spp	3	11,000	22,000	17,000	114	228	GF
1322	Wenchman	Pristipomoides aquilonaris	22	13,000	21,000	17,000	1,053	1,668	GF
1323	Spiny butterfly ray	Gymnura altavela	53	10,000	23,000	16,000	2,321	5,150	GO
1324	Scaly gurnard	Lepidotrigla brachyoptera	7	14,000	18,000	16,000	398	526	GF
1325	Whitefin weakfish	Cynoscion albus	56	16,000	16,000	16,000	3,450	3,495	GG
1326	Bighead tilefish	Caulolatilus affinis	32	12,000	20,000	16,000	1,582	2,589	GF[1]
1327	Torpedo rays	Torpedo spp	82	8,800	23,000	16,000	3,546	9,314	GC
1328	Pacific barracuda	Sphyraena argentea	26	3,000	29,000	16,000	884	8,520	GG
1329	Crest-tail catsharks nei	Galeus spp	3	16,000	16,000	16,000	172	172	GG
1330	Pelagic thresher	Alopias pelagicus	2,221	15,000	16,000	16,000	137,466	143,349	GG
1331	Brownspotted grouper	Epinephelus chlorostigma	52	7,200	24,000	16,000	2,156	7,282	GG
1332	Percarina	Percarina demidoffi	13	11,000	20,000	15,000	670	1,276	GF
1333	Sand weakfish	Cynoscion arenarius	32	14,000	16,000	15,000	1,964	2,348	GG
1334	Herrings, sardines nei	Clupeidae	1	13,000	17,000	15,000	35	45	GF
1335	Brown-marbled grouper	Epinephelus fuscoguttatus	50	6,700	23,000	15,000	2,147	7,553	GG
1336	Four-spot megrim	Lepidorhombus boscii	26	12,000	18,000	15,000	1,454	2,166	GF
1337	Piper gurnard	Trigla lyra	7	13,000	17,000	15,000	402	510	GF
1338	Big skate	Raja binoculata	45	9,100	20,000	15,000	2,209	4,972	GG
1339	God's flounder	Cyclopsetta panamensis	20	9,400	20,000	15,000	985	2,073	GF
1340	Western Atlantic seabream	Archosargus rhomboidalis	25	11,000	18,000	15,000	1,361	2,268	GG
1341	Black musselcracker	Cymatoceps nasutus	6	12,000	17,000	14,000	329	457	GF
1342	Cachema weakfish	Cynoscion phoxocephalus	50	14,000	14,000	14,000	3,454	3,499	GG
1343	Parrot grunt	Pomadasys perotaei	2	14,000	14,000	14,000	149	149	GG
1344	Brown ray	Raja miraletus	42	8,500	19,000	14,000	2,182	4,987	GG
1345	Offshore silver hake	Merluccius albidus	7	10,000	19,000	14,000	395	708	GG
1346	White skate	Raja alba	42	8,400	19,000	13,000	2,232	4,980	GG
1347	South American catfish	Rhamdia quelen	11	12,000	14,000	13,000	800	905	GF

1348	Scalloped hammerhead	Sphyrna lewini	257	2,600	23,000	13,000	11,000	100,000	S1
1349	Capaz	Pimelodus grosskopfii	11	12,000	13,000	13,000	800	905	GF
1350	Broad-striped anchovy	Anchoa hepsetus	0.2	13,000	13,000	13,000	12	12	S2
1351	Swordspine snook	Centropomus ensiferus	20	3,900	21,000	12,000	952	5,073	GF
1352	Silvergray rockfish	Sebastes brevispinis	5	11,000	14,000	12,000	336	416	GG
1353	Sawtooth barracuda	Sphyraena putnamae	20	2,400	22,000	12,000	876	8,338	GG
1354	Spotted grouper	Epinephelus analogus	42	5,300	19,000	12,000	2,148	7,825	GG
1355	Eyespot skate	Raja cyclophora	38	7,500	17,000	12,000	2,213	4,995	GG
1356	Night shark	Carcharhinus signatus	343	9,900	14,000	12,000	24,507	34,769	GG
1357	Crocodile icefishes nei	Channichthyidae	8	9,800	14,000	12,000	579	831	GO[1]
1358	Aurora rockfish	Sebastes aurora	4	10,000	13,000	12,000	340	420	GG
1359	Live sharksucker	Echeneis naucrates	1	6,400	16,000	11,000	88	226	GO
1360	Oceanic whitetip shark	Carcharhinus longimanus	529	5,500	17,000	11,000	30,999	96,140	S1
1361	Small-scaled terapon	Terapon puta	1	6,300	16,000	11,000	82	207	GO
1362	Painted comber	Serranus scriba	13	5,200	17,000	11,000	787	2,547	GF
1363	Spottail seabream	Diplodus holbrooki	5	9,100	13,000	11,000	358	503	GF
1364	Summan grouper	Epinephelus summana	36	4,800	17,000	11,000	2,152	7,534	GG
1365	Sharpnose stingray	Himantura gerrardi	39	7,100	14,000	11,000	2,676	5,428	GO
1366	Japanese topeshark	Hemitriakis japanica	54	6,000	15,000	11,000	3,497	8,937	GF
1367	Mediterranean flyingfish	Cheilopogon heterurus	1	5,300	16,000	11,000	56	169	GO
1368	Steenbrasses nei	Lithognathus spp	5	8,900	12,000	11,000	370	509	GF
1369	Bigspined boarfish	Pentaceros decacanthus	7	7,100	14,000	11,000	500	1,000	S2
1370	Zebra catfish	Brachyplatystoma juruense	9	9,600	11,000	10,000	800	905	GG
1371	Angelsharks, sand devils nei	Squatinidae	274	10,000	10,000	10,000	27,000	27,000	M1
1372	Whiteleg skate	Raja taaf	32	6,300	14,000	10,000	2,269	5,013	GG
1373	Threadfin rockling	Gaidropsarus ensis	4	4,400	16,000	10,000	233	844	GF
1374	Panama spadefish	Parapsettus panamensis	0.5	6,600	13,000	10,000	35	72	GC
1375	Yelloweye rockfish	Sebastes ruberrimus	3	9,100	11,000	9,900	318	381	GG
1376	Mouse catshark	Galeus murinus	2	9,600	9,600	9,600	172	172	GG
1377	Bigeye lates	Lates mariae	15	3,000	16,000	9,400	941	4,974	GG
1378	Lethrinus spp	Lethrinus spp	4	7,700	11,000	9,300	368	515	GG
1379	Large-scaled gurnard	Lepidotrigla cavillone	4	8,000	11,000	9,200	396	522	GF
1380	Smooth hammerhead	Sphyrna zygaena	180	1,800	16,000	9,100	11,000	100,000	GG
1381	Tiger shark	Galeocerdo cuvier	72	650	17,000	9,000	4,140	110,000	S1
1382	Red gurnard perch	Helicolenus percoides	2	8,900	8,900	8,900	275	275	GG
1383	Redstripe rockfish	Sebastes proriger	3	7,700	9,500	8,600	336	417	GG
1384	Common eagle ray	Myliobatis aquila	29	5,400	11,000	8,400	2,512	5,322	GO
1385	Speckled hind	Epinephelus drummondhayi	28	3,700	13,000	8,400	2,145	7,595	GG
1386	Blackfin snapper	Lutjanus buccanella	10	4,400	12,000	8,300	819	2,260	GG
1387	Fine flounder	Paralichthys adspersus	11	5,300	11,000	8,200	990	2,067	GG
1388	Longtail stingray	Dasyatis longa	28	5,300	11,000	8,200	2,507	5,303	GO
1389	Spottail shark	Carcharhinus sorrah	235	6,600	9,500	8,100	24,621	35,706	GG
1390	Rock sea bass	Centropristis philadelphica	5	5,300	11,000	8,000	454	908	GG
1391	Eelpout	Zoarces viviparus	54	8,000	8,000	8,000	6,804	6,804	GO
1392	Shortbill spearfish	Tetrapturus angustirostris	543	5,700	10,000	8,000	53,182	94,898	GG
1393	Whitecheek shark	Carcharhinus dussumieri	214	6,800	9,100	7,900	23,646	31,454	GG
1394	Sattar snowtrout	Schizothorax curvifrons	0.2	6,200	9,700	7,900	21	32	GF
1395	African bullhead	Lophiobagrus cyclurus	7	7,900	7,900	7,900	830	830	GF
1396	Chrysichthys stappersii	Chrysichthys stappersii	7	7,800	7,800	7,800	830	830	GG
1397	Antarctic silverfish	Pleuragramma antarcticum	1	7,800	7,800	7,800	110	110	S1
1398	Atlantic goldeneye tilefish	Caulolatilus chrysops	7	7,600	8,000	7,800	906	951	S3
1399	Spotted weever	Trachinus araneus	2	7,600	7,600	7,600	230	230	GG
1400	Brownstripe red snapper	Lutjanus vitta	9	3,800	11,000	7,400	791	2,335	GG
1401	Comet grouper	Epinephelus morrhua	25	3,300	12,000	7,400	2,152	7,504	GG
1402	Northern cods nei	Gadus spp	4	2,700	12,000	7,400	295	1,306	GG
1403	Xenistius californiensis	Xenistius californiensis	1	4,900	9,800	7,300	112	225	GF
1404	Brook trout	Salvelinus fontinalis	10	4,900	9,700	7,300	1,000	2,000	S2
1405	Danube sturgeon(=Osetr)	Acipenser gueldenstaedtii	96	6,800	7,600	7,200	12,632	14,076	GG
1406	Tonguesole nei	Cynoglossus spp	6	4,300	10,000	7,200	579	1,351	GO
1407	Common Atlantic grenadier	Nezumia aequalis	4	5,100	8,900	7,000	420	730	GF
1408	California sheephead	Semicossyphus pulcher	38	5,600	8,400	7,000	4,536	6,804	S2
1409	Pike icefish	Champsocephalus esox	7	6,800	7,100	7,000	1,017	1,060	GO[1]
1410	Microchirus azevia	Microchirus azevia	4	1,100	13,000	7,000	300	3,500	GF
1411	Sunfish	Mola spp	25	6,800	7,100	6,900	3,546	3,683	GO[1]
1412	African pompano	Alectis ciliaris	13	6,900	6,900	6,900	1,816	1,816	GG
1413	Red steenbras	Petrus rupestris	3	5,700	8,100	6,900	363	518	GF
1414	Black snook	Centropomus nigrescens	11	2,200	11,000	6,800	944	4,997	GF
1415	Starry sturgeon	Acipenser stellatus	60	6,000	7,400	6,700	8,000	9,890	S1
1416	Bigeyes,glasseyes,bulleyes nei	Priacanthidae	1	2,700	10,000	6,500	56	219	GF
1417	Duckbill barracudina	Magnisudis atlantica	0.2	5,700	7,100	6,400	28	35	GO
1418	Sandbar shark	Carcharhinus plumbeus	365	5,400	7,300	6,300	50,000	68,000	S1
1419	Lesser weever	Trachinus vipera	1	6,300	6,300	6,300	230	230	GG
1420	White sturgeon	Acipenser transmontanus	113	6,300	6,300	6,300	18,000	18,000	S1
1421	Patagonian redfish	Sebastes oculatus	2	5,500	6,700	6,100	342	421	GG
1422	Stingrays nei	Dasyatis spp	19	3,600	8,500	6,100	2,245	5,260	GO
1423	Yellowfin snook	Centropomus robalito	9	1,900	10,000	6,000	941	4,970	GF
1424	Yellowmouth rockfish	Sebastes reedi	2	5,200	6,500	5,900	336	419	GG
1425	Bluestriped chub	Sectator ocyurus	1	3,300	8,300	5,800	88	220	GO
1426	Starry smooth-hound	Mustelus asterias	15	3,700	7,800	5,700	1,934	4,012	GG
1427	Mediterranean slimehead	Hoplostethus mediterraneus	8	5,700	5,700	5,700	1,400	1,400	GG
1428	American conger	Conger oceanicus	35	3,800	7,600	5,700	4,545	9,090	S2
1429	Lusitanian toadfish	Halobatrachus didactylus	0.3	3,700	7,600	5,700	33	68	GC
1430	Solea spp	Solea spp	3	890	10,000	5,700	300	3,500	GG
1431	Colorado snapper	Lutjanus colorado	7	3,200	7,900	5,500	881	2,162	GG
1432	Blue maomao	Scorpis violacea	1	3,200	7,800	5,500	83	205	GO
1433	Pizzaro stardrum	Stellifer pizarroensis	0.5	3,800	7,100	5,500	69	129	GF
1434	Pink maomao	Caprodon longimanus	7	2,500	8,400	5,500	871	2,891	GF
1435	Longjaw leatherjacket	Oligoplites altus	0.5	2,600	8,200	5,400	56	177	GF
1436	Sculpins	Myoxocephalus spp	8	4,500	6,100	5,300	1,361	1,814	GF
1437	Broadnose skate	Bathyraja brachyurops	18	3,500	7,000	5,200	2,569	5,215	GF[1]
1438	Morocco dentex	Dentex maroccanus	2	2,700	7,700	5,200	216	605	GG
1439	Arrowhead dogfish	Deania profundorum	13	5,100	5,100	5,100	2,612	2,612	GG
1440	Rooster hind	Epinephelus acanthistius	17	2,500	7,700	5,100	2,165	6,557	GG
1441	Sailray	Raja lintea	15	3,000	6,500	4,800	2,338	5,078	GG
1442	Kibonde	Chrysichthys brachynema	4	4,800	4,800	4,800	830	830	GG
1443	Atlantic puffers nei	Sphoeroides spp	2	4,600	4,900	4,700	450	480	GO
1444	Inshore sand perch	Diplectrum pacificum	6	2,200	7,200	4,700	797	2,581	GF
1445	Treefish	Sebastes serriceps	2	4,200	5,000	4,600	327	395	GG
1446	Cownose ray	Rhinoptera bonasus	15	2,800	6,300	4,600	2,316	5,198	GO
1447	Pacific guitarfish	Rhinobatos planiceps	364	4,600	4,600	4,600	80,000	80,000	GF
1448	Roudi escolar	Promethichthys prometheus	9	3,000	5,900	4,500	1,500	3,000	GF
1449	Dealfish	Trachipterus arcticus	164	4,400	4,400	4,400	37,100	37,100	GO
1450	Toothed flounder	Cyclopsetta querna	6	2,800	6,000	4,400	986	2,082	GF
1451	Spinetail ray	Bathyraja spinicauda	28	4,300	4,300	4,300	6,500	6,500	GG

1452 Southern kingcroaker	Menticirrhus americanus	1	2,000	6,600	4,300	136	454	GG
1453 Mobula nei	Mobula spp	13	2,500	6,000	4,300	2,181	5,158	GO
1454 Blackchin guitarfish	Rhinobatos cemiculus	340	4,300	4,300	4,300	80,000	80,000	GF
1455 Mottled grouper	Mycteroperca rubra	9	990	7,300	4,200	1,196	8,883	GG
1456 Brycon amazonicus	Brycon amazonicus	15	3,500	4,800	4,200	3,160	4,300	GF
1457 White snook	Centropomus viridis	7	1,300	7,000	4,100	941	4,970	GF
1458 Yellowfin grouper	Mycteroperca venenosa	28	4,100	4,100	4,100	6,810	6,810	S1
1459 Blacknose shark	Carcharhinus acronotus	121	3,400	4,800	4,100	25,298	35,576	GG
1460 Black carp	Mylopharyngodon piceus	60	4,000	4,000	4,000	15,000	15,000	S1
1461 South Georgia icefish	Pseudochaenichthys georgianus	3	3,600	4,400	4,000	785	958	GO[1]
1462 Copper mahseer	Neolissochilus hexagonolepis	0.1	3,100	4,900	4,000	21	32	GF
1463 Porbeagle	Lamna nasus	535	4,000	4,000	4,000	135,000	135,000	S1
1464 Weatherfish	Misgurnus fossilis	0.2	3,100	4,800	3,900	31	49	GO
1465 Mediterranean barbel	Barbus meridionalis	1	610	7,300	3,900	117	1,404	GG
1466 Ladyfishes nei	Elops spp	4	3,100	4,700	3,900	907	1,361	GG
1467 Brycon dentex	Brycon dentex	14	3,300	4,500	3,900	3,160	4,300	GF
1468 Valparaiso chromis	Chromis crusma	0.5	2,200	5,500	3,900	88	221	GO
1469 Fourspot flounder	Paralichthys oblongus	5	2,400	5,100	3,700	978	2,086	GG
1470 Caribbean sharpnose shark	Rhizoprionodon porosus	57	3,400	4,000	3,700	14,407	16,625	GF
1471 Longnose stingray	Dasyatis guttata	11	2,100	5,100	3,600	2,153	5,167	GO
1472 Black grouper	Mycteroperca bonaci	15	800	6,400	3,600	2,268	18,140	S2
1473 Smoothmouth sea catfish	Arius heudelotii	2	1,300	5,800	3,600	300	1,300	GF
1474 Atlantic lizardfish	Synodus saurus	0.1	3,200	3,900	3,500	28	35	GF
1475 Greenspotted rockfish	Sebastes chlorostictus	1	3,200	3,900	3,500	334	409	GG
1476 Greenstriped rockfish	Sebastes elongatus	1	3,100	3,800	3,500	340	422	GG
1477 Banded rudderfish	Seriola zonata	24	2,400	4,500	3,400	5,290	9,904	GG
1478 Sharpchin rockfish	Sebastes zacentrus	1	3,000	3,700	3,400	337	418	GG
1479 Imperial blackfish	Schedophilus ovalis	6	1,800	4,700	3,200	1,350	3,522	GF
1480 Queen triggerfish	Balistes vetula	2	3,200	3,200	3,200	491	491	GG
1481 Cunner	Tautogolabrus adspersus	4	1,200	5,100	3,200	778	3,202	GF
1482 Longfin gurnard	Chelidonichthys obscurus	2	2,400	3,900	3,100	484	764	GG
1483 Eaton's skate	Bathyraja eatonii	15	2,500	3,700	3,100	4,035	5,932	GF[1]
1484 Thor's scaldfish	Arnoglossus thori	2	1,800	4,300	3,100	579	1,351	GO
1485 Pompano dolphinfish	Coryphaena equiselis	15	3,000	3,000	3,000	4,940	4,940	GG
1486 Black snapper	Apsilus dentatus	4	2,300	3,600	3,000	1,035	1,667	GF
1487 Atlantic weasel shark	Paragaleus pectoralis	22	2,700	3,100	2,900	6,846	7,895	GO
1488 Granulated catfish	Pterodoras granulosus	2	2,900	3,000	2,900	559	581	GO
1489 Kerguelen sandpaper skate	Bathyraja irrasa	11	2,000	3,800	2,900	2,850	5,420	GF[1]
1490 Chrysichthys platycephalus	Chrysichthys platycephalus	2	2,900	2,900	2,900	830	830	GG
1491 Balistes spp	Balistes spp	1	2,900	2,900	2,900	491	491	GG
1492 Rainbow wrasse	Coris julis	2	1,000	4,700	2,900	508	2,389	GF
1493 Pacific goliath grouper	Epinephelus quinquefasciatus	9	1,400	4,300	2,900	2,165	6,557	GG
1494 Shortspine African angler	Lophius vaillanti	20	2,400	3,200	2,800	6,238	8,345	GG
1495 Lings nei	Molva spp	13	2,100	3,500	2,800	3,793	6,430	GG
1496 Reticulated leatherjacket	Stephanolepis diaspros	0.5	2,500	3,000	2,800	161	195	GO
1497 Broomtail grouper	Mycteroperca xenarcha	50	1,800	3,700	2,700	13,608	27,216	S1
1498 Skilfish	Erilepis zonifer	8	1,800	3,600	2,700	2,268	4,536	GF
1499 Arctic skate	Raja hyperborea	4	2,200	3,200	2,700	1,201	1,731	S2
1500 Sickle pomfret	Taractichthys steindachneri	19	2,400	2,900	2,700	6,600	8,036	S1
1501 Canary dentex	Dentex canariensis	1	1,400	3,900	2,600	216	605	GG
1502 Thresher	Alopias vulpinus	312	2,500	2,800	2,600	113,500	124,000	S1
1503 Olive rockfish	Sebastes serranoides	1	2,300	2,800	2,600	321	384	GG
1504 Tanganyika lates	Lates angustifrons	4	820	4,300	2,600	938	4,947	GG
1505 Smalleye moray cod	Muraenolepis microps	1	1,200	3,800	2,500	178	542	GO
1506 Bull shark	Carcharhinus leucas	77	2,000	3,000	2,500	25,885	38,103	GG
1507 Warsaw grouper	Epinephelus nigritus	49	1,400	3,600	2,500	13,608	36,364	S2
1508 Common stingray	Dasyatis pastinaca	9	1,700	3,300	2,500	2,831	5,562	GO
1509 Zebra tilefish	Branchiostegus semifasciatus	7	1,400	3,500	2,500	2,000	5,000	GF
1510 Capro dory	Capromimus abbreviatus	3	1,700	3,200	2,400	800	1,500	GF
1511 Brycon spp	Brycon spp	9	2,100	2,800	2,400	3,160	4,300	GF
1512 Brycon orbignyanus	Brycon orbignyanus	9	2,100	2,800	2,400	3,160	4,300	GF
1513 Brown moray	Gymnothorax unicolor	2	700	4,100	2,400	515	2,998	GO
1514 Slender codling	Halargyreus johnsonii	1	1,200	3,600	2,400	308	932	GF
1515 Patagonian rockcod	Patagonotothen brevicauda	19	1,800	2,900	2,400	6,561	10,749	GF
1516 Pacific pomfret	Brama japonica	2	1,300	3,300	2,300	600	1,500	GG
1517 Lesser amberjack	Seriola fasciata	17	1,500	2,900	2,200	5,688	10,883	GG
1518 Sharptooth jobfish	Pristipomoides typus	3	1,800	2,600	2,200	1,103	1,618	GF
1519 Glacier lantern fish	Benthosema glaciale	0.1	1,400	3,000	2,200	34	70	GC
1520 Scalyfin weakfish	Cynoscion squamipinnis	7	2,100	2,200	2,200	3,450	3,495	GG
1521 Rock hind	Epinephelus adscensionis	7	920	3,300	2,100	2,143	7,757	GG
1522 Huchen	Hucho hucho	5	1,500	2,700	2,100	1,900	3,379	GF
1523 Barbeled houndshark	Leptocharias smithii	18	1,900	2,300	2,100	7,930	9,321	GO
1524 Spotback skate	Raja castelnaui	6	1,300	2,900	2,100	2,217	5,008	GG
1525 True tunas nei	Thunnus spp	15	480	3,600	2,000	4,132	30,829	GG
1526 Ticon cownose ray	Rhinoptera brasiliensis	6	1,200	2,800	2,000	2,153	5,167	GO
1527 Longbill spearfish	Tetrapturus pfluegeri	138	1,400	2,600	2,000	53,294	95,422	GG
1528 Sterlet sturgeon	Acipenser ruthenus	31	1,900	2,100	2,000	14,902	15,840	GG
1529 Nurse shark	Ginglymostoma cirratum	172	1,500	2,400	2,000	70,450	114,500	S1
1530 Ocean sunfish	Mola mola	279	280	3,700	2,000	76,400	1,000,000	S1
1531 Barrelfish	Hyperoglyphe perciformis	11	1,900	1,900	1,900	5,670	5,670	GG
1532 Giant guitarfish	Rhynchobatus djiddensis	156	1,900	1,900	1,900	80,000	80,000	S2
1533 Northern kingfish	Menticirrhus saxatilis	0.4	880	2,900	1,900	136	454	GG
1534 Smalltail shark	Carcharhinus porosus	59	1,600	2,200	1,900	26,791	36,002	GG
1535 Antarctic starry skate	Raja georgiana	10	1,800	2,000	1,900	5,000	5,400	S1
1536 Caspian shad	Alosa caspia	1	1,700	2,100	1,900	245	297	GG
1537 Pacific sleeper shark	Somniosus pacificus	3	490	3,300	1,900	844	5,669	GF
1538 Australian bonito	Sarda australis	3	1,500	2,100	1,800	1,267	1,701	GG
1539 Ocellated wedge sole	Dicologlossa hexophthalma	1	280	3,200	1,800	300	3,500	GF
1540 Thickback sole	Microchirus variegatus	1	280	3,200	1,700	300	3,500	GF
1541 Basketwork eel	Diastobranchus capensis	7	1,700	1,700	1,700	4,245	4,245	S2
1542 Volga pikeperch	Sander volgensis	3	1,300	2,100	1,700	1,299	2,159	GG
1543 Starry rockfish	Sebastes constellatus	1	1,400	1,800	1,600	338	414	GG
1544 Lemon shark	Negaprion brevirostris	25	1,500	1,700	1,600	14,524	16,766	GF
1545 Barracudinas nei	Notolepis spp	0.1	1,400	1,700	1,500	29	37	GO
1546 Fourbeard rockling	Enchelyopus cimbrius	0.4	640	2,400	1,500	166	622	GF
1547 Creole-fish	Paranthias furcifer	2	700	2,300	1,500	859	2,863	GF
1548 Bignose fanskate	Sympterygia acuta	5	950	2,100	1,500	2,285	5,026	GF
1549 Brazilian sharpnose shark	Rhizoprionodon lalandii	22	1,300	1,500	1,400	14,407	16,624	GF
1550 Oxydoras kneri	Oxydoras kneri	1	1,400	1,500	1,400	539	559	GO
1551 Sicklefin smooth-hound	Mustelus lunulatus	3	740	2,100	1,400	1,492	4,217	GG
1552 Copper shark	Carcharhinus brachyurus	43	1,200	1,700	1,400	25,917	37,166	GG
1553 Spanish ling	Molva macrophthalma	7	1,100	1,800	1,400	3,836	6,439	GG
1554 Camouflage grouper	Epinephelus polyphekadion	5	610	2,200	1,400	2,139	7,620	GG
1555 Dogtooth grouper	Epinephelus caninus	5	610	2,200	1,400	2,141	7,552	GG

1556	Clingfishes nei	Gobiesocidae	0.1	920	1,800	1,400	38	75	GC
1557	Deepbody boarfish	Antigonia capros	0.1	1,000	1,700	1,400	30	48	GF
1558	Muraena augusti	Muraena augusti	1	390	2,300	1,300	517	2,979	GO
1559	Antarctic escolar	Paradiplospinus antarcticus	3	880	1,800	1,300	1,500	3,000	GF
1560	Bigeye thresher	Alopias superciliosus	206	1,300	1,300	1,300	160,000	160,000	S1
1561	Finetooth shark	Carcharhinus isodon	39	1,100	1,500	1,300	26,731	35,490	GG
1562	Yellowfin notothen	Patagonotothen guntheri	11	970	1,600	1,300	6,873	11,160	GF
1563	Brown comber	Serranus hepatus	1	590	1,900	1,200	773	2,494	GF
1564	Daisy stingray	Dasyatis margarita	4	730	1,800	1,200	2,153	5,167	GO
1565	Bigeye grenadier	Macrourus holotrachys	4	1,200	1,200	1,200	3,153	3,153	S1
1566	Curlfin sole	Pleuronichthys decurrens	1	810	1,600	1,200	615	1,229	GF
1567	Spottail spiny turbot	Psettodes belcheri	1	1,200	1,200	1,200	1,200	1,200	GG
1568	Cadenat's sole	Pegusa cadenati	1	190	2,200	1,200	300	3,500	GF
1569	Longfin mako	Isurus paucus	37	1,200	1,200	1,200	31,300	31,300	GG
1570	Graysby	Cephalopholis cruentata	1	550	1,800	1,200	300	1,000	GG
1571	Blackfin icefish	Chaenocephalus aceratus	1	1,200	1,200	1,200	1,088	1,088	S2
1572	Pacific pompano	Peprilus simillimus	0.2	500	1,900	1,200	80	300	GG
1573	Hornyhead turbot	Pleuronichthys verticalis	1	780	1,600	1,200	576	1,157	GF
1574	Spinner shark	Carcharhinus brevipinna	38	890	1,400	1,200	26,149	42,413	GG
1575	Redbanded seabream	Pagrus auriga	1	910	1,400	1,100	892	1,325	GG
1576	Whiptail stingray	Dasyatis brevis	3	670	1,600	1,100	2,173	5,161	GO
1577	Mediterranean spearfish	Tetrapturus belone	76	810	1,400	1,100	52,905	93,621	GG
1578	Imperial scaldfish	Arnoglossus imperialis	1	650	1,500	1,100	579	1,351	GO
1579	Snaggletooth	Borostomias antarcticus	0.1	740	1,400	1,100	35	67	GC
1580	Leopard shark	Triakis semifasciata	7	510	1,700	1,100	3,993	12,955	GF
1581	Cardinal snapper	Pristipomoides macrophthalmus	1	840	1,300	1,100	1,061	1,654	GF
1582	California butterfly ray	Gymnura marmorata	4	680	1,400	1,000	2,727	5,481	GO
1583	Brazilian sandperch	Pinguipes brasilianus	2	1,000	1,000	1,000	1,820	1,877	GF
1584	Black bullhead	Ameiurus melas	1	1,000	1,000	1,000	500	500	GG
1585	Misty grouper	Epinephelus mystacinus	3	430	1,500	970	2,151	7,677	GG
1586	Red piranha	Pygocentrus nattereri	4	810	1,100	960	3,160	4,300	GF
1587	Creole wrasse	Clepticus parrae	1	330	1,600	960	440	2,147	GF
1588	Daggertooth	Anotopterus pharao	0.03	820	1,000	920	28	35	GO
1589	Caml grenadier	Macrourus caml	1	680	1,200	910	488	833	GF[1]
1590	Rosy rockfish	Sebastes rosaceus	0.3	840	990	910	303	357	GG
1591	Patagonian skate	Bathyraja macloviana	3	570	1,200	910	2,290	5,029	GF[1]
1592	Little sleeper shark	Somniosus rostratus	1	230	1,600	890	843	5,679	GF
1593	Norwegian skate	Raja nidarosiensis	3	560	1,200	870	2,401	5,129	GG
1594	Mako sharks	Isurus spp	27	870	870	870	31,300	31,300	GG
1595	Bonitos nei	Sarda spp	2	660	980	820	1,525	2,288	GG
1596	Auchenionchus microcirrhis	Auchenionchus microcirrhis	0.2	750	840	790	267	301	GO
1597	Polygon moray	Gymnothorax polygonius	1	220	1,300	750	517	2,979	GO
1598	Chionobathyscus dewitti	Chionobathyscus dewitti	1	750	750	750	1,088	1,088	GF
1599	Elongate tonguesole	Symphurus ligulatus	1	460	1,000	750	580	1,303	GO
1600	Stout beardfish	Polymixia nobilis	1	620	860	740	1,042	1,441	GO
1601	Pacu	Piaractus mesopotamicus	3	630	850	740	3,160	4,300	GF
1602	Dogfishes nei	Squalus spp	1	190	1,300	740	835	5,781	GG
1603	Ocean pout	Macrozoarces americanus	5	730	730	730	6,804	6,804	GO
1604	Bonnethead	Sphyrna tiburo	14	140	1,300	730	11,000	100,000	GG
1605	Grass porgy	Calamus arctifrons	0.3	590	800	700	311	423	GF
1606	Angola rockfish	Scorpaena angolensis	0.3	610	750	680	332	407	GF
1607	Great lanternshark	Etmopterus princeps	1	180	1,200	680	844	5,666	GF
1608	Dealfishes	Trachipterus spp	25	680	680	680	37,100	37,100	GO
1609	Beluga	Huso huso	35	350	1,000	670	35,000	100,000	S1
1610	Great hammerhead	Sphyrna mokarran	13	130	1,200	670	11,000	100,000	GG
1611	Alfonsinos, etc. nei	Berycidae	1	400	900	650	724	1,637	GF
1612	Black driftfish	Hyperoglyphe bythites	4	640	640	640	5,670	5,670	GG
1613	Wide-eyed flounder	Bothus podas	1	380	890	630	579	1,351	GO
1614	Marbled rockcod	Notothenia rossii	5	470	770	620	6,758	10,999	GF
1615	Fortune jack	Seriola peruana	3	420	780	600	3,621	6,804	S2
1616	Bluntnose sixgill shark	Hexanchus griseus	28	460	690	580	40,000	60,000	S1
1617	Iheringichthys labrosus	Iheringichthys labrosus	0.5	530	600	570	800	905	GF
1618	Spiny icefish	Chaenodraco wilsoni	1	510	510	510	1,088	1,088	GF
1619	Brown smooth-hound	Mustelus henlei	3	390	590	490	4,536	6,804	S2
1620	Broadnose sevengill shark	Notorynchus cepedianus	11	480	480	480	22,545	22,545	S3
1621	Spiny turbot	Psettodes bennettii	1	470	470	470	1,200	1,200	GG
1622	Darwin's slimehead	Gephyroberyx darwinii	1	460	460	460	1,400	1,400	GF
1623	Pigeye shark	Carcharhinus amboinensis	16	310	590	450	27,242	52,318	GG
1624	Giant blenny	Scartichthys gigas	0.1	400	450	430	266	299	GO
1625	Seahorses nei	Hippocampus spp	0.02	270	560	420	36	73	GC
1626	Speckled ray	Raja polystigma	1	250	580	410	2,155	5,002	GG
1627	Silver chimaera	Chimaera phantasma	0.4	410	410	410	852	852	GO
1628	Graery threadfin seabass	Cratinus agassizii	1	190	630	410	798	2,585	GF
1629	Plunket shark	Centroscymnus plunketi	1	110	710	410	844	5,663	GF
1630	Oplegnathus spp	Oplegnathus spp	0.05	220	560	390	88	221	GO
1631	Bramble shark	Echinorhinus brucus	1	95	640	370	856	5,763	GO
1632	Giant seabass	Stereolepis gigas	3	350	370	360	7,458	7,844	GF
1633	Pacific cownose ray	Rhinoptera steindachneri	1	220	490	350	2,272	5,132	GO
1634	Elephantfishes, etc. nei	Callorhinchidae	0.3	350	350	350	852	852	GF
1635	Painted eel	Echelus myrus	0.3	100	580	340	515	2,992	GO
1636	Devil fish	Mobula mobular	1	210	440	330	2,633	5,410	GO
1637	Common guitarfish	Rhinobatos rhinobatos	25	310	310	310	80,000	80,000	GF
1638	Brown wrasse	Labrus merula	0.2	100	500	300	401	2,000	GG
1639	Slender smooth-hound	Gollum attenuatus	2	270	320	300	6,192	7,296	GO
1640	Atlantic fanfish	Pterycombus brama	0.3	170	420	290	602	1,504	GF
1641	Polla drum	Umbrina xanti	0.2	280	300	290	766	822	GG
1642	Lagocephalus spp	Lagocephalus spp	0.04	260	310	280	115	141	GO
1643	Muraena spp	Muraena spp	0.3	84	480	280	517	2,978	GO
1644	Rough ray	Raja radula	1	170	400	280	2,151	5,004	GG
1645	Cowcod	Sebastes levis	0.1	250	310	280	326	403	GG
1646	Green sturgeon	Acipenser medirostris	3	250	290	270	11,303	12,962	GG
1647	Bathyraja rays nei	Bathyraja spp	2	250	250	250	6,500	6,500	M1
1648	Spotted ratfish	Hydrolagus colliei	0.2	230	230	230	852	852	GO
1649	Hawaiian ladyfish	Elops hawaiensis	0.3	180	280	230	907	1,361	GG
1650	Senegalese ladyfish	Elops senegalensis	0.3	180	280	230	907	1,361	GG
1651	Fantail flounder	Xystreurys liolepis	0.3	140	300	220	989	2,069	GF
1652	Island grouper	Mycteroperca fusca	1	78	360	220	1,819	8,506	GG
1653	Hooknose	Agonus cataphractus	0.1	200	230	220	426	506	GO
1654	Sticklebacks	Gasterosteus spp	0.01	150	290	220	38	76	GC
1655	Combtooth blennies	Blenniidae	0.1	200	220	210	233	250	GO
1656	Murray's skate	Bathyraja murrayi	1	150	260	200	3,162	5,575	GF[1]
1657	Marbled electric ray	Torpedo marmorata	1	130	260	200	3,713	7,376	GC
1658	Guinean barracuda	Sphyraena afra	0.3	38	360	200	874	8,294	GG
1659	Mediterranean dealfish	Trachipterus trachypterus	7	200	200	200	37,100	37,100	GO

1660	Whitespotted grouper	Epinephelus caeruleopunctatus	1	89	300	190	2,164	7,274	GG
1661	Straightnose rabbitfish	Rhinochimaera atlantica	0.2	180	180	180	852	852	GO
1662	Sailfin roughshark	Oxynotus paradoxus	0.3	43	290	170	853	5,780	GO
1663	Chola guitarfish	Rhinobatos percellens	13	160	160	160	80,000	80,000	GF
1664	Sharptooth houndshark	Triakis megalopterus	1	80	240	160	3,594	10,608	GF
1665	Ocellated icefish	Chionodraco rastrospinosus	0.2	150	150	150	1,088	1,088	GF
1666	Dusky shark	Carcharhinus obscurus	5	110	170	140	28,786	42,766	GG
1667	Fringebarbel sturgeon	Acipenser nudiventris	1	120	130	130	10,744	12,474	GG
1668	Chilean torpedo	Torpedo tremens	1	87	160	120	3,696	6,862	GC
1669	Humped rockcod	Notothenia gibberifrons	1	92	150	120	6,611	10,803	GF
1670	Combers nei	Serranus spp	0.2	51	170	110	871	2,936	GF
1671	Triangular rockcod	Notothenia acuta	1	80	130	110	6,753	10,999	GF
1672	Dogtooth grenadier	Cynomacrurus piriei	0.1	68	120	94	420	730	GF
1673	Yellowmouth grouper	Mycteroperca interstitialis	0.3	29	150	90	1,651	8,552	GG
1674	Dog snapper	Lutjanus jocu	0.1	42	130	86	772	2,361	GG
1675	Slender armorhead	Pseudopentaceros wheeleri	0.1	50	100	75	500	1,000	GF
1676	Cuckoo wrasse	Labrus mixtus	0.1	25	120	75	401	2,000	GG
1677	Sandpaper fish	Paratrachichthys trailli	0.1	71	71	71	1,400	1,400	GF
1678	Stolzmann's weakfish	Cynoscion stolzmanni	0.3	70	71	70	3,533	3,561	GG
1679	Bignose shark	Carcharhinus altimus	2	45	86	66	27,242	52,318	GG
1680	Black rockcod	Notothenia coriiceps	1	50	81	65	6,810	11,078	GF
1681	Round ray	Raja fyllae	0.2	41	90	65	2,284	5,043	GG
1682	Great white shark	Carcharodon carcharias	8	59	59	59	141,000	141,000	S1
1683	Smalleyed rabbitfish	Hydrolagus affinis	0.1	59	59	59	852	852	GO
1684	Nototheniops nybelini	Nototheniops nybelini	0.4	38	60	49	6,668	10,620	GF
1685	Sand tiger shark	Carcharias taurus	2	47	47	47	35,810	35,854	GO
1686	Blackspotted smooth-hound	Mustelus punctulatus	0.1	24	67	45	1,492	4,217	GG
1687	Plunderfish	Pogonophryne permitini	0.01	24	62	43	81	204	GO
1688	Chionodraco hamatus	Chionodraco hamatus	0.04	35	35	35	1,088	1,088	GF
1689	Roughskin dogfish	Centroscymnus owstoni	0.1	9	59	34	840	5,709	GF
1690	Scoophead	Sphyrna media	1	7	59	33	11,000	100,000	GG
1691	Roughtail stingray	Dasyatis centroura	0.1	20	45	32	2,203	5,116	GO
1692	King of herrings	Regalecus glesne	1	28	28	28	37,100	37,100	GO
1693	Conger eels nei	Conger spp	0.2	27	27	27	9,080	9,100	GG
1694	Crocodile shark	Pseudocarcharias kamoharai	1	20	20	20	34,767	34,808	GO
1695	Sharpnose sevengill shark	Heptranchias perlo	1	18	22	20	31,519	38,037	GF
1696	Roundscale spearfish	Tetrapturus georgii	1	14	25	20	53,539	96,579	GG
1697	Antarctic horsefish	Zanclorhynchus spinifer	0.01	17	21	19	410	495	GO
1698	Sevan trout	Salmo ischchan	0.1	13	25	19	1,991	3,976	GG
1699	Basking shark	Cetorhinus maximus	74	15	23	19	3,300,000	5,000,000	S1
1700	Painted notie	Nototheniops larseni	0.2	13	22	18	6,867	11,150	GF
1701	Marlins nei	Makaira spp	2	16	16	16	131,270	135,120	GG
1702	Pelagic stingray	Dasyatis violacea	0.1	10	22	16	2,270	5,096	GO
1703	Carcharhinus sharks nei	Carcharhinus spp	1	11	21	16	27,242	52,318	GG
1704	Madeiran ray	Raja maderensis	0.1	10	21	15	2,381	5,034	GG
1705	Yellowbelly rockcod	Notothenia neglecta	0.1	10	16	13	6,072	10,097	GF
1706	Bluespotted seabass	Cephalopholis taeniops	0.01	5	17	11	300	1,000	GG
1707	Psammobatis sand skates nei	Psammobatis spp	0.03	6	15	11	2,165	4,999	GF
1708	McCain's skate	Bathyraja maccaini	0.1	9	11	10	5,143	6,240	GF[1]
1709	Little gulper shark	Centrophorus uyato	0.01	2	15	9	845	5,656	GF
1710	Hammerhead sharks nei	Sphyrna spp	0.2	2	15	8	11,000	100,000	GG
1711	Parachaenichthys georgianus	Parachaenichthys georgianus	0.001	5	12	8	85	216	GO
1712	Southern opah	Lampris immaculatus	0.2	6	6	6	37,100	37,100	GG
1713	Black sole	Achlyopa nigra	0.003	1	10	5	300	3,500	GF
1714	Striped-eyed rockcod	Notothenia kempi	0.04	4	6	5	6,952	11,262	GF
1715	Dogfish sharks, etc. nei	Squaliformes	0.01	1	7	4	876	5,819	GO
1716	Toad notie	Nototheniops mizops	0.01	1	2	2	6,818	11,082	GF
1717	Houndsharks, smoothhounds nei	Triakidae	0.005	1	2	2	1,985	6,013	GF
1718	Rough longnose dogfish	Deania hystricosa	0.004	1	1	1	2,612	2,612	GG
1719	Smalleye hammerhead	Sphyrna tudes	0.02	0.2	2	1	11,000	100,000	GG
1720	Sand devil	Squatina dumeril	0.01	1	1	1	14,000	18,000	GG
1721	Antarctic armless flounder	Mancopsetta maculata	0.001	0.4	1	1	579	1,351	GO
1722	Centroscymnus spp	Centroscymnus spp	0.001	0.1	1	0.3	845	5,656	GF
1723	Spined pygmy shark	Squaliolus laticaudus	0.001	0.1	1	0.3	845	5,656	GF
1724	Smalltooth sand tiger	Odontaspis ferox	0.01	0.3	0.3	0.3	32,843	32,853	GO
1725	Striped rockcod	Pagothenia hansoni	0.002	0.2	0.3	0.3	6,471	10,627	GF
	**Total**		**77,348,260**	1,100,000,000,000	2,200,000,000,000	1,600,000,000,000			

[1] The GEMW type for this species category varies between years, due to changes in tonnages for related species categories.

Key for EMW/GEMW types

**EMW/ GEMW type**	**EMW/GEMW**	**Type**
S1	EMW	Single-species EMW - based on rank 1 data (average, mean or usual weights, including mature fishes caught in survey fishing)
S2	EMW	Single-species EMW - based on rank 2 data (common weights, simple weight ranges, other survey fishing mean weights)
S3	EMW	Single-species EMW - based on rank 3 data (fish lengths)
M1	EMW	Multi-species EMW - based on rank 1 data (average, mean or usual weights)
M2	EMW	Multi-species EMW - based on rank 2 data (common weights, simple weight ranges)
GG	GEMW	GEMW extrapolated from estimates for species in same genus.
GF	GEMW	GEMW extrapolated from estimates for species in same family.
GO	GEMW	GEMW extrapolated from estimates for species in same order.
GC	GEMW	GEMW extrapolated from estimates for species in same class.
GA	GEMW	Overall GEMW extrapolated from all EMW estimates.

This table shows estimated number ranges of finfishes caught from the wild, on average, each year between 2000 and 2019, totalling 1.1-2.2 trillion or 1.1-2.2 x 10^{12} individuals. Number ranges are calculated for each species category from the capture tonnage, reported by the FAO, and the estimated mean weight range (EMW) obtained in the present study. EMWs are based on fish size data for the species. Where no such data was obtained, generic mean weights (GEMWs) extrapolated from EMWs were used.

GEMWs are extrapolated from EMW estimates for other species in the same genus, where possible. Otherwise, where possible, for other species in the same family. Otherwise, where possible, for species in the same order or class. Otherwise from the overall GEMW, extrapolated from all EMWs. Source of capture tonnage: FAO (2021a).
