# Supplementary material for: Estimating global numbers of fishes caught from the wild annually from 2000 to 2019
Source: Anim Welf. 2024 Feb 8;33:e6. doi: 10.1017/awf.2024.7 (PMC10951671; doi:10.1017/awf.2024.7)
Supplement: Mood and Brooke supplementary material 4 — Mood and Brooke supplementary material [file S0962728624000071sup004.pdf]

Estimated wild-caught finfish numbers by year (2000-2019)

Year	Capture production (landings) (million tonnes)[1]	Estimated numbers in millions (2 significant figures)[1]			% of estimate based on specific species/genus weight data[2] (by tonnage)	Mean individual fish weight for year (g)[3]	
		Lower	Upper	Midpoint		Lower	Upper
1999	77.7	1,200,000	2,500,000	1,800,000	73%	31	66
2000	79.2	1,300,000	2,800,000	2,100,000	74%	28	61
2001	76.9	1,200,000	2,300,000	1,800,000	73%	33	67
2002	77.1	1,200,000	2,600,000	1,900,000	73%	29	63
2003	75.1	1,000,000	2,100,000	1,500,000	72%	36	74
2004	79.8	1,200,000	2,600,000	1,900,000	74%	31	68
2005	79.5	1,200,000	2,500,000	1,800,000	73%	32	68
2006	76.3	1,000,000	2,100,000	1,600,000	72%	36	75
2007	76.7	1,000,000	2,200,000	1,600,000	72%	35	74
2008	75.9	1,100,000	2,200,000	1,600,000	72%	34	71
2009	76.2	1,100,000	2,200,000	1,600,000	71%	34	71
2010	74.0	1,000,000	1,900,000	1,500,000	69%	39	74
2011	78.1	1,100,000	2,400,000	1,800,000	72%	32	68
2012	75.0	960,000	2,000,000	1,500,000	70%	38	78
2013	75.9	970,000	2,000,000	1,500,000	70%	38	78
2014	75.4	910,000	1,700,000	1,300,000	69%	44	83
2015	76.9	1,000,000	1,900,000	1,500,000	70%	40	77
2016	76.6	890,000	1,700,000	1,300,000	68%	45	86
2017	79.7	990,000	1,900,000	1,500,000	69%	41	80
2018	83.3	1,100,000	2,300,000	1,700,000	70%	37	76
2019	79.4	980,000	1,900,000	1,400,000	68%	41	81
Average 2000-2019	**77.3**	**1,100,000**	**2,200,000**	**1,600,000**	**71%**	**36**	**73**
Average 1999-2007	77.6	1,100,000	2,400,000	1,800,000	73%	32	68

Inter-year differences in millions (to 2 significant figures)	
Midpoint in lowest year (2016)	1,300,000
Midpoint in highest year (2000)	2,100,000
Difference	**780,000**
Difference by species:	
Anchoveta(=Peruvian anchovy)	540,000
Marine fishes nei	110,000
Sandeels(=sandlances) nei	66,000
Capelin	50,000
Others	3,600

This table shows estimated finfish numbers, by year between 2000-2019, for the main estimate (see text). The respective lowest and highest estimates, i.e. midpoints, were for 2016 and 2000, with a difference of 780 billion (7.8×10^{11}). This difference is mainly comprised of anchoveta, and to a lesser extent "Marine fishes nei", sandeels and capelin. "Others" includes all other finfish species. Estimates for each of these changed by between -24 and + 29 billion.

Notes

1. Source of capture production tonnage (landings): FAO (2021a). Source of estimated numbers: present study.

2. This column shows the percentage of the estimate for the year, by tonnage, that was based on estimated mean weights (EMWs) and/or generic estimated mean weights (GEMWs) calculated for the specific genus. Estimates that are largely based on such data, being based on data for the same or closely related species, are expected to be more reliable.

3. The mean individual fish weight for each year is back-calculated from the total capture tonnage and total estimated fish numbers.
