# Supplementary material for: Estimating global numbers of fishes caught from the wild annually from 2000 to 2019
Source: Anim Welf. 2024 Feb 8;33:e6. doi: 10.1017/awf.2024.7 (PMC10951671; doi:10.1017/awf.2024.7)
Supplement: Mood and Brooke supplementary material 5 — Mood and Brooke supplementary material [file S0962728624000071sup005.pdf]

Estimated average annual wild-caught finfish numbers (2000-2019) by country[1]

Country	Average annual capture production (landings) 2000-2019 (tonnes)[2]	Estimated numbers (2 significant figures)[2]			% of estimate based on specific species/genus weight data[3] (by tonnage)	Mean individual fish weight for country (g)[4]		Top species for country by estimated numbers (midpoint)[5]
		Lower	Upper	Midpoint		Lower	Upper	
Peru	6,192,074	200,000,000,000	570,000,000,000	390,000,000,000	98%	11	31	Anchoveta(=Peruvian anchovy)
China	10,230,292	130,000,000,000	220,000,000,000	170,000,000,000	56%	48	78	Marine fishes nei
Chile	2,838,559	58,000,000,000	150,000,000,000	110,000,000,000	97%	19	49	Anchoveta(=Peruvian anchovy)
Indonesia	4,905,646	62,000,000,000	140,000,000,000	100,000,000,000	67%	35	79	Stolephorus anchovies nei
India	3,743,288	55,000,000,000	100,000,000,000	78,000,000,000	30%	37	68	Cyprinids nei
Denmark	814,384	53,000,000,000	56,000,000,000	54,000,000,000	100%	15	15	Sandeels(=Sandlances) nei
Morocco	1,044,744	39,000,000,000	43,000,000,000	41,000,000,000	92%	24	27	European pilchard(=Sardine)
Myanmar	1,759,060	26,000,000,000	54,000,000,000	40,000,000,000	0.4%	33	67	Marine fishes nei
Viet Nam	1,916,265	26,000,000,000	53,000,000,000	39,000,000,000	4%	36	74	Marine fishes nei
Philippines	1,989,051	25,000,000,000	52,000,000,000	39,000,000,000	77%	38	80	Stolephorus anchovies nei
Japan	3,126,999	31,000,000,000	44,000,000,000	38,000,000,000	86%	70	100	Japanese anchovy
Thailand	1,791,092	24,000,000,000	48,000,000,000	36,000,000,000	41%	37	76	Marine fishes nei
Russian Federation	3,808,819	17,000,000,000	40,000,000,000	29,000,000,000	94%	95	219	European sprat
Norway	2,280,759	17,000,000,000	35,000,000,000	26,000,000,000	100%	65	135	Sandeels(=Sandlances) nei
Bangladesh	1,415,493	17,000,000,000	34,000,000,000	25,000,000,000	26%	41	84	Freshwater fishes nei
Turkey	408,880	12,000,000,000	38,000,000,000	25,000,000,000	92%	11	33	European anchovy
Malaysia	1,189,732	16,000,000,000	32,000,000,000	24,000,000,000	46%	37	77	Marine fishes nei
Iceland	1,403,123	9,500,000,000	29,000,000,000	19,000,000,000	100%	48	147	Capelin
South Africa	625,421	15,000,000,000	19,000,000,000	17,000,000,000	98%	33	43	Southern African anchovy
Korea, Republic of	1,170,189	14,000,000,000	19,000,000,000	16,000,000,000	84%	63	83	Japanese anchovy
United States of America	3,847,475	8,400,000,000	18,000,000,000	13,000,000,000	97%	213	457	Gulf menhaden
Mexico	1,202,796	9,900,000,000	16,000,000,000	13,000,000,000	83%	75	122	California pilchard
Sweden	229,610	11,000,000,000	12,000,000,000	12,000,000,000	100%	18	20	European sprat
Cambodia	481,430	7,100,000,000	15,000,000,000	11,000,000,000	0%	33	68	Freshwater fishes nei
Poland	190,558	9,000,000,000	10,000,000,000	9,600,000,000	91%	19	21	European sprat
Tanzania, United Rep. of	359,697	6,800,000,000	11,000,000,000	9,000,000,000	69%	32	53	Silver cyprinid
Ukraine	181,075	6,300,000,000	11,000,000,000	8,400,000,000	71%	17	29	European sprat
Uganda	383,170	6,300,000,000	9,900,000,000	8,100,000,000	79%	39	61	Silver cyprinid
Namibia	490,693	1,600,000,000	14,000,000,000	7,900,000,000	98%	34	301	Cape horse mackerel
Spain	866,156	4,800,000,000	10,000,000,000	7,400,000,000	86%	87	179	European pilchard(=Sardine)
Mauritania	338,907	5,500,000,000	9,000,000,000	7,300,000,000	78%	37	62	Sardinellas nei
Pakistan	458,393	4,400,000,000	8,500,000,000	6,400,000,000	40%	54	104	Freshwater fishes nei
Ghana	360,925	3,200,000,000	9,600,000,000	6,400,000,000	70%	38	114	European anchovy
Latvia	128,053	5,700,000,000	6,900,000,000	6,300,000,000	98%	19	22	European sprat
Iran (Islamic Rep. of)	496,800	4,800,000,000	7,500,000,000	6,100,000,000	85%	66	104	Black and Caspian Sea sprat
Egypt	351,056	3,700,000,000	8,500,000,000	6,100,000,000	71%	41	95	Silversides(=Sand smelts) nei
Sri Lanka	366,963	3,900,000,000	8,100,000,000	6,000,000,000	45%	45	95	Clupeoids nei
Italy	157,748	2,800,000,000	8,400,000,000	5,600,000,000	83%	19	56	European anchovy
Brazil	679,773	4,000,000,000	6,600,000,000	5,300,000,000	47%	103	168	Marine fishes nei
Kenya	147,203	3,900,000,000	6,600,000,000	5,200,000,000	91%	22	38	Silver cyprinid
Congo, Dem. Rep. of the	232,506	3,400,000,000	7,000,000,000	5,200,000,000	1%	33	69	Freshwater fishes nei
Angola	332,593	2,600,000,000	7,200,000,000	4,900,000,000	71%	47	126	Sardinellas nei
Nigeria	590,458	3,600,000,000	5,800,000,000	4,700,000,000	56%	102	164	Sardinellas nei
Estonia	77,668	4,400,000,000	4,700,000,000	4,600,000,000	99%	16	17	European sprat
Ecuador	506,253	3,200,000,000	5,500,000,000	4,300,000,000	90%	93	159	Anchoveta(=Peruvian anchovy)
Faroe Islands	523,289	2,100,000,000	6,100,000,000	4,100,000,000	99%	86	247	Blue whiting(=Poutassou)
Germany	231,605	3,400,000,000	4,700,000,000	4,000,000,000	93%	49	68	European sprat
Netherlands	413,001	2,500,000,000	5,200,000,000	3,800,000,000	99%	79	166	European pilchard(=Sardine)
Senegal	418,932	2,200,000,000	5,200,000,000	3,700,000,000	81%	80	187	Madeiran sardinella
Canada	517,178	1,600,000,000	5,700,000,000	3,700,000,000	93%	91	314	Capelin
Algeria	113,006	3,200,000,000	4,100,000,000	3,600,000,000	87%	28	36	European pilchard(=Sardine)
Mozambique	160,516	2,400,000,000	4,800,000,000	3,600,000,000	1%	33	66	Marine fishes nei
Taiwan Province of China	779,546	2,100,000,000	5,000,000,000	3,600,000,000	85%	155	363	Pacific saury
France	432,122	2,400,000,000	4,400,000,000	3,400,000,000	94%	99	180	European pilchard(=Sardine)
Portugal	183,751	2,900,000,000	3,600,000,000	3,200,000,000	94%	51	64	European pilchard(=Sardine)
United Kingdom	524,196	2,200,000,000	3,900,000,000	3,000,000,000	97%	134	239	European sprat
Lithuania	126,361	2,100,000,000	4,000,000,000	3,000,000,000	98%	32	60	European sprat
Korea, Dem. People's Rep	183,660	1,800,000,000	3,800,000,000	2,800,000,000	35%	48	102	Marine fishes nei
Finland	158,994	2,300,000,000	3,300,000,000	2,800,000,000	99%	49	70	European sprat
Panama	201,998	2,200,000,000	3,200,000,000	2,700,000,000	88%	63	92	Pacific anchoveta
Oman	210,680	2,200,000,000	3,200,000,000	2,700,000,000	63%	67	97	Indian oil sardine
China, Hong Kong SAR	141,248	1,700,000,000	3,400,000,000	2,600,000,000	19%	41	82	Marine fishes nei
Belize	156,832	1,300,000,000	3,500,000,000	2,400,000,000	97%	44	118	European anchovy
Croatia	49,316	1,900,000,000	2,900,000,000	2,400,000,000	95%	17	26	European pilchard(=Sardine)
Argentina	589,249	1,800,000,000	3,000,000,000	2,400,000,000	90%	194	333	Argentine anchovy
Georgia	48,027	910,000,000	3,800,000,000	2,300,000,000	97%	13	53	European anchovy
Ireland	227,576	1,600,000,000	3,000,000,000	2,300,000,000	97%	77	139	Boarfish
Australia	126,889	1,400,000,000	2,900,000,000	2,100,000,000	33%	44	92	Clupeoids nei
Greece	68,990	1,300,000,000	2,900,000,000	2,100,000,000	83%	24	54	European anchovy
Chad	92,063	1,400,000,000	2,800,000,000	2,100,000,000	0%	33	68	Freshwater fishes nei
Cameroon	161,158	1,500,000,000	2,600,000,000	2,100,000,000	53%	62	106	Freshwater fishes nei
Yemen	165,817	1,500,000,000	2,500,000,000	2,000,000,000	56%	66	109	Pelagic percomorphs nei
Turkmenistan	14,616	1,500,000,000	2,300,000,000	1,900,000,000	100%	6	10	Black and Caspian Sea sprat
Madagascar	106,095	1,200,000,000	2,500,000,000	1,900,000,000	8%	43	86	Marine fishes nei
Zambia	78,893	1,200,000,000	2,400,000,000	1,800,000,000	0%	33	64	Freshwater fishes nei

Guinea	163,539	1,400,000,000	2,200,000,000	1,800,000,000	58%	73	118	Freshwater fishes nei
Tunisia	87,368	1,300,000,000	2,000,000,000	1,700,000,000	82%	44	65	European pilchard(=Sardine)
Sierra Leone	162,494	1,200,000,000	1,800,000,000	1,500,000,000	78%	90	135	Bonga shad
Venezuela, Boliv Rep of	271,184	880,000,000	2,100,000,000	1,500,000,000	72%	129	307	Round sardinella
Malawi	99,269	1,300,000,000	1,500,000,000	1,400,000,000	61%	67	78	Lake Malawi sardine
United Arab Emirates	82,837	690,000,000	1,700,000,000	1,200,000,000	50%	48	120	Stolephorus anchovies nei
Congo	65,585	680,000,000	1,400,000,000	1,000,000,000	38%	47	96	Freshwater fishes nei
Lao People's Dem. Rep.	41,981	710,000,000	1,400,000,000	1,000,000,000	0%	31	59	Freshwater fishes nei
New Zealand	422,182	540,000,000	1,400,000,000	980,000,000	88%	296	781	Jack and horse mackerels nei
Saint Vincent/Grenadines	42,257	460,000,000	1,400,000,000	960,000,000	96%	29	91	European anchovy
Greenland	120,661	450,000,000	1,300,000,000	890,000,000	100%	91	266	Capelin
Colombia	98,285	580,000,000	960,000,000	770,000,000	56%	102	169	Pacific anchoveta
Côte d'Ivoire	67,983	490,000,000	960,000,000	720,000,000	66%	71	139	Marine fishes nei
Somalia	28,420	420,000,000	870,000,000	650,000,000	0%	32	67	Marine fishes nei
Togo	24,249	260,000,000	1,000,000,000	640,000,000	79%	24	95	European anchovy
Central African Republic	27,250	400,000,000	830,000,000	610,000,000	0%	33	68	Freshwater fishes nei
Mali	98,476	370,000,000	830,000,000	600,000,000	66%	119	265	Freshwater fishes nei
Sudan (former)	37,707	340,000,000	760,000,000	550,000,000	42%	50	110	Freshwater fishes nei
Guyana	26,465	360,000,000	740,000,000	550,000,000	6%	36	74	Marine fishes nei
Azerbaijan	4,256	400,000,000	630,000,000	510,000,000	95%	7	11	Black and Caspian Sea sprat
Iraq	34,590	360,000,000	660,000,000	510,000,000	37%	52	95	Cyprinids nei
Suriname	24,666	320,000,000	660,000,000	490,000,000	8%	38	77	Marine fishes nei
Bulgaria	5,940	470,000,000	500,000,000	490,000,000	97%	12	13	European sprat
Cyprus	24,939	190,000,000	740,000,000	460,000,000	98%	34	131	European anchovy
Nepal	20,337	300,000,000	620,000,000	460,000,000	0%	33	68	Freshwater fishes nei
Fiji	39,216	290,000,000	600,000,000	450,000,000	46%	65	136	Marine fishes nei
Niger	36,346	300,000,000	590,000,000	440,000,000	40%	62	121	Freshwater fishes nei
Papua New Guinea	230,923	280,000,000	570,000,000	420,000,000	93%	407	822	Freshwater fishes nei
Zanzibar	25,287	240,000,000	540,000,000	390,000,000	13%	46	105	Clupeoids nei
Bolivia (Plurinat.State)	6,741	160,000,000	570,000,000	370,000,000	1%	12	41	Silversides(=Sand smelts) nei
Uruguay	81,300	250,000,000	470,000,000	360,000,000	87%	172	320	Whitemouth croaker
Gambia	41,708	290,000,000	410,000,000	350,000,000	78%	103	146	Bonga shad
Libya	36,397	190,000,000	460,000,000	320,000,000	76%	79	193	Marine fishes nei
Rwanda	15,169	280,000,000	370,000,000	320,000,000	27%	41	55	Lake Tanganyika sardine
South Sudan	14,000	210,000,000	420,000,000	320,000,000	0%	33	68	Freshwater fishes nei
Zimbabwe	14,766	250,000,000	370,000,000	310,000,000	19%	40	60	Dagaas (=Kapenta)
Burundi	15,278	250,000,000	350,000,000	300,000,000	23%	43	60	Lake Tanganyika sprat
Benin	38,413	210,000,000	380,000,000	300,000,000	72%	100	181	Freshwater fishes nei
Saint Kitts and Nevis	20,370	180,000,000	420,000,000	300,000,000	93%	49	114	Jack and horse mackerels nei
Gabon	35,225	230,000,000	360,000,000	290,000,000	64%	99	153	Freshwater fishes nei
Maldives	139,727	180,000,000	400,000,000	290,000,000	90%	351	780	Marine fishes nei
Haiti	12,279	180,000,000	380,000,000	280,000,000	0%	33	67	Marine fishes nei
Saudi Arabia	50,928	180,000,000	360,000,000	270,000,000	53%	142	281	Spinefeet(=Rabbitfishes) nei
Comoros	21,489	160,000,000	340,000,000	250,000,000	80%	63	133	Sardinellas nei
Jamaica	10,905	160,000,000	340,000,000	250,000,000	0%	33	67	Marine fishes nei
Cuba	21,764	160,000,000	310,000,000	240,000,000	40%	70	136	Marine fishes nei
El Salvador	39,606	150,000,000	310,000,000	230,000,000	73%	126	266	Marine fishes nei
Trinidad and Tobago	13,133	130,000,000	280,000,000	210,000,000	26%	47	98	Marine fishes nei
Vanuatu	97,797	83,000,000	320,000,000	200,000,000	98%	308	1,176	Chilean jack mackerel
Solomon Islands	42,502	130,000,000	270,000,000	200,000,000	80%	157	333	Marine fishes nei
Kazakhstan	36,529	140,000,000	250,000,000	200,000,000	81%	147	256	Freshwater fishes nei
Equatorial Guinea	5,492	120,000,000	260,000,000	190,000,000	8%	21	44	Clupeoids nei
Ethiopia	27,811	140,000,000	220,000,000	180,000,000	83%	127	193	Cyprinids nei
Sudan	14,495	110,000,000	240,000,000	170,000,000	56%	60	137	Freshwater fishes nei
Micronesia, Fed.States of	51,739	99,000,000	220,000,000	160,000,000	88%	238	523	Marine fishes nei
Costa Rica	18,212	100,000,000	210,000,000	160,000,000	36%	87	179	Marine fishes nei
Other nei	62,023	88,000,000	200,000,000	150,000,000	98%	305	709	Sardinellas nei
Cabo Verde	20,681	91,000,000	190,000,000	140,000,000	72%	109	228	Pelagic percomorphs nei
Martinique	3,723	84,000,000	170,000,000	130,000,000	10%	21	44	Clupeoids nei
Liberia	12,256	87,000,000	170,000,000	130,000,000	56%	72	141	Freshwater fishes nei
Burkina Faso	15,348	92,000,000	160,000,000	130,000,000	55%	94	167	Freshwater fishes nei
Samoa	8,036	76,000,000	160,000,000	120,000,000	36%	52	106	Marine fishes nei
Syrian Arab Republic	6,227	72,000,000	140,000,000	110,000,000	25%	43	86	Freshwater fishes nei
French Polynesia	13,184	67,000,000	140,000,000	100,000,000	63%	94	196	Marine fishes nei
Kiribati	78,593	56,000,000	150,000,000	100,000,000	90%	537	1,414	Marine fishes nei
Romania	5,329	89,000,000	110,000,000	99,000,000	94%	49	60	European sprat
Paraguay	19,755	71,000,000	130,000,000	99,000,000	0%	157	277	Freshwater fishes nei
Albania	4,985	58,000,000	130,000,000	95,000,000	79%	38	87	European anchovy
Guatemala	18,899	53,000,000	110,000,000	83,000,000	79%	166	355	Freshwater fishes nei
Bahrain	6,898	55,000,000	110,000,000	82,000,000	27%	63	125	Spinefeet(=Rabbitfishes) nei
Guadeloupe	4,927	54,000,000	110,000,000	82,000,000	10%	45	92	Marine fishes nei
Dominican Republic	10,402	53,000,000	110,000,000	81,000,000	46%	95	195	Marine fishes nei
Eritrea	4,975	65,000,000	93,000,000	79,000,000	29%	53	77	Lizardfishes nei
Uzbekistan	12,015	53,000,000	98,000,000	76,000,000	76%	122	228	Freshwater fishes nei
Guinea-Bissau	6,388	49,000,000	98,000,000	73,000,000	22%	65	130	Marine fishes nei
Timor-Leste	3,165	47,000,000	97,000,000	72,000,000	0.1%	33	67	Marine fishes nei
Brunei Darussalam	4,059	46,000,000	93,000,000	69,000,000	28%	44	88	Marine fishes nei
Seychelles	88,669	37,000,000	100,000,000	69,000,000	96%	872	2,400	Marine fishes nei
Qatar	13,065	43,000,000	95,000,000	69,000,000	36%	138	302	Marine fishes nei
Honduras	5,881	45,000,000	93,000,000	69,000,000	49%	63	132	Marine fishes nei
Lebanon	3,426	37,000,000	94,000,000	65,000,000	24%	36	93	Clupeoids nei
Marshall Islands	60,440	36,000,000	93,000,000	64,000,000	97%	652	1,680	Marine fishes nei
Hungary	6,538	45,000,000	73,000,000	59,000,000	75%	90	146	Cyprinids nei

Belgium	21,811	37,000,000	78,000,000	58,000,000	90%	278	589	Pouting(=Bib)
Sao Tome and Principe	6,317	36,000,000	79,000,000	57,000,000	45%	80	174	Marine fishes nei
Mauritius	12,524	34,000,000	73,000,000	54,000,000	44%	171	368	Marine fishes nei
Nicaragua	19,128	23,000,000	51,000,000	37,000,000	80%	372	839	Marine fishes nei
Falkland Is.(Malvinas)	22,592	25,000,000	42,000,000	33,000,000	63%	543	897	Marine fishes nei
Slovenia	826	25,000,000	40,000,000	33,000,000	93%	21	33	European pilchard(=Sardine)
Palestine	2,080	22,000,000	41,000,000	32,000,000	68%	50	93	Sardinellas nei
Afghanistan	1,295	19,000,000	39,000,000	29,000,000	0%	33	68	Freshwater fishes nei
Tonga	2,138	19,000,000	39,000,000	29,000,000	32%	55	114	Marine fishes nei
Israel	2,823	19,000,000	38,000,000	29,000,000	45%	74	146	Marine fishes nei
Mayotte	9,165	18,000,000	38,000,000	28,000,000	86%	241	521	Marine fishes nei
China, Macao SAR	1,020	15,000,000	31,000,000	23,000,000	0%	32	67	Marine fishes nei
Serbia	2,392	16,000,000	29,000,000	23,000,000	69%	84	145	Freshwater fishes nei
Kuwait	3,082	15,000,000	25,000,000	20,000,000	71%	121	204	Marine fishes nei
Palau	1,453	13,000,000	27,000,000	20,000,000	38%	54	111	Marine fishes nei
Barbados	2,329	10,000,000	29,000,000	19,000,000	34%	81	227	Flyingfishes nei
Singapore	1,601	12,000,000	24,000,000	18,000,000	32%	66	133	Marine fishes nei
British Virgin Islands	1,148	12,000,000	24,000,000	18,000,000	29%	47	96	Marine fishes nei
Tuvalu	6,419	11,000,000	24,000,000	18,000,000	89%	263	582	Marine fishes nei
Montenegro	844	12,000,000	23,000,000	18,000,000	56%	37	69	European pilchard(=Sardine)
French Guiana	1,919	9,900,000	21,000,000	15,000,000	46%	92	193	Marine fishes nei
Serbia and Montenegro	512	7,200,000	16,000,000	12,000,000	33%	31	71	Freshwater fishes nei
Antigua and Barbuda	1,594	7,600,000	16,000,000	12,000,000	2%	101	211	Surgeonfishes nei
Jordan	639	7,400,000	15,000,000	11,000,000	21%	42	87	Freshwater fishes nei
Netherlands Antilles	8,082	6,100,000	15,000,000	10,000,000	96%	555	1,322	Marine fishes nei
Saint Lucia	1,588	6,100,000	13,000,000	9,600,000	71%	121	258	Marine fishes nei
Cook Islands	4,175	6,100,000	13,000,000	9,400,000	91%	329	682	Marine fishes nei
Curaçao	13,346	4,900,000	13,000,000	9,200,000	98%	994	2,731	Marine fishes nei
Anguilla	417	6,000,000	12,000,000	9,100,000	0%	34	70	Marine fishes nei
Djibouti	1,561	5,200,000	12,000,000	8,700,000	41%	128	299	Marine fishes nei
Switzerland	1,829	5,300,000	12,000,000	8,600,000	97%	153	343	Whitefishes nei
Tajikistan	668	5,900,000	11,000,000	8,600,000	48%	59	113	Freshwater fishes nei
Wallis and Futuna Is.	380	5,600,000	12,000,000	8,600,000	0%	33	68	Marine fishes nei
Dominica	817	5,600,000	11,000,000	8,500,000	54%	71	147	Marine fishes nei
Austria	358	5,300,000	11,000,000	8,100,000	0%	33	68	Freshwater fishes nei
Belarus	784	6,400,000	9,400,000	7,900,000	77%	83	123	Cyprinids nei
New Caledonia	3,010	4,900,000	10,000,000	7,600,000	80%	291	613	Marine fishes nei
Bosnia and Herzegovina	295	4,400,000	9,000,000	6,700,000	0%	33	68	Freshwater fishes nei
Malta	1,666	4,600,000	8,400,000	6,500,000	92%	198	363	Clupeoids nei
Nauru	2,107	4,000,000	8,800,000	6,400,000	88%	239	524	Marine fishes nei
US Virgin Islands	518	3,000,000	6,300,000	4,600,000	17%	82	173	Marine fishes nei
Channel Islands	630	4,200,000	4,700,000	4,500,000	61%	133	149	Sandeels(=Sandlances) nei
Grenada	2,348	3,000,000	5,300,000	4,200,000	88%	440	786	Bigeye scad
Czechia	4,155	3,000,000	5,000,000	4,000,000	98%	832	1,387	Freshwater fishes nei
Puerto Rico	914	2,300,000	5,100,000	3,700,000	55%	179	389	Marine fishes nei
Mongolia	161	2,400,000	4,900,000	3,600,000	0%	33	68	Freshwater fishes nei
Cayman Islands	125	1,900,000	3,800,000	2,900,000	0%	32	67	Marine fishes nei
Slovakia	1,782	2,200,000	3,500,000	2,900,000	97%	515	794	Common carp
Bahamas	1,049	1,700,000	3,900,000	2,800,000	81%	267	617	Marine fishes nei
Armenia	423	1,900,000	3,800,000	2,800,000	83%	113	228	Freshwater fishes nei
St. Pierre and Miquelon	2,914	1,500,000	3,900,000	2,700,000	92%	746	1,881	Atlantic cod
Sint Maarten	108	1,600,000	3,300,000	2,500,000	0%	32	67	Marine fishes nei
Réunion	3,022	1,400,000	3,500,000	2,400,000	90%	864	2,180	Marine fishes nei
Guam	510	1,400,000	3,100,000	2,300,000	76%	164	354	Marine fishes nei
North Macedonia	215	1,300,000	3,200,000	2,300,000	41%	67	162	Freshwater fishes nei
Bermuda	362	1,300,000	2,800,000	2,100,000	74%	128	272	Clupeoids nei
French Southern Terr	77	1,100,000	2,400,000	1,800,000	0%	32	67	Marine fishes nei
Bonaire/S.Eustatius/Saba	68	1,000,000	2,100,000	1,600,000	0%	32	67	Marine fishes nei
Saint Helena	452	980,000	2,100,000	1,500,000	85%	213	462	Marine fishes nei
Saint Barthélemy	65	970,000	2,000,000	1,500,000	0%	32	67	Marine fishes nei
Eswatini	61	890,000	1,800,000	1,400,000	0%	33	68	Freshwater fishes nei
Saint-Martin	59	870,000	1,800,000	1,300,000	0%	32	67	Marine fishes nei
Tokelau	117	850,000	1,800,000	1,300,000	50%	66	137	Marine fishes nei
Turks and Caicos Is.	49	730,000	1,500,000	1,100,000	0.3%	33	67	Marine fishes nei
Northern Mariana Is.	538	620,000	1,600,000	1,100,000	91%	341	863	Marine fishes nei
Aruba	155	720,000	1,500,000	1,100,000	42%	105	216	Marine fishes nei
American Samoa	4,257	390,000	1,700,000	1,100,000	99%	2,481	10,777	Albacore
Niue	150	590,000	1,200,000	910,000	55%	123	254	Marine fishes nei
Montserrat	35	520,000	1,100,000	800,000	0%	32	67	Marine fishes nei
Botswana	185	630,000	930,000	780,000	90%	199	294	Freshwater fishes nei
Lesotho	46	380,000	780,000	580,000	44%	59	120	Freshwater fishes nei
Bhutan	18	320,000	610,000	460,000	0%	30	57	Freshwater fishes nei
Kyrgyzstan	66	290,000	480,000	390,000	91%	138	227	Cyprinids nei
Moldova, Republic of	46	220,000	400,000	310,000	75%	115	213	Freshwater fishes nei
Isle of Man	37	120,000	220,000	170,000	69%	165	310	Finfishes nei
British Indian Ocean Ter	20	58,000	120,000	89,000	74%	162	336	Marine fishes nei
Pitcairn Islands	3	51,000	100,000	78,000	0%	32	67	Marine fishes nei
Monaco	2	23,000	48,000	35,000	0%	32	67	Marine fishes nei
Gibraltar	1	15,000	31,000	23,000	0%	32	67	Marine fishes nei
**All countries combined**	**77,348,260**	**1,100,000,000,000**	**2,200,000,000,000**	**1,600,000,000,000**	**71%**	**36**	**73**	**Anchoveta(=Peruvian anchovy)**

Notes

1. Countries are shown in descending order of estimated numbers (midpoint).

2. Source of capture production tonnage (landings): FAO (2021a). Source of estimated numbers: present study.

3. This column shows the percentage of the estimate for the country, by tonnage, that was based on estimated mean weights (EMWs) and/or generic estimated mean weights (GEMWs) calculated for the specific genus. Estimates that are largely based on such data, being based on data for the same or closely related species, are expected to be more reliable.

4. The mean individual fish weight for each country is back-calculated from the total capture tonnage and total estimated fish numbers.

5. The top species is the species category with the highest midpoint of estimated fish numbers. Note that this may represent only a small percentage of total numbers caught by a country, in cases where no single category of species dominates capture numbers. For scientific names of species, see Table S3 of the supplementary material.
