# Supplementary material for: Estimating global numbers of fishes caught from the wild annually from 2000 to 2019
Source: Anim Welf. 2024 Feb 8;33:e6. doi: 10.1017/awf.2024.7 (PMC10951671; doi:10.1017/awf.2024.7)
Supplement: Mood and Brooke supplementary material 6 — Mood and Brooke supplementary material [file S0962728624000071sup006.pdf]

Estimated finfish numbers destined for reduction to fishmeal and oil (2010)

**Species[1]**	**Scientific name**	**Percent of total capture destined for reduction[1] %**	**Capture destined for reduction ('000 tonnes)[2]**	**Lower estimated mean weight (EMW/ GEMW)[3] (g). GEMWs in italics**	**Upper estimated mean weight (EMW/ GEMW)[3] (g). GEMWs in italics**	**Lower estimated numbers (2 significant figures)[4]**	**Upper estimated numbers (2 significant figures)[4]**
Anchoveta(=Peruvian anchovy)	*Engraulis ringens*	28.9	4,803	10	29	170,000,000,000	480,000,000,000
South American pilchard	*Sardinops sagax*	3.7	615	*90*	*159*	3,900,000,000	6,800,000,000
Chilean jack mackerel	*Trachurus murphyi*	3.4	565	200	1,000	570,000,000	2,800,000,000
Capelin	*Mallotus villosus*	0.9	150	17	50	3,000,000,000	8,800,000,000
Atlantic herring	*Clupea harengus*	2.3	382	100	600	640,000,000	3,800,000,000
Gulf menhaden	*Brevoortia patronus*	2.5	415	95	127	3,300,000,000	4,400,000,000
Sandeels(=Sandlances) nei	*Ammodytes spp*	3	499	10	10	50,000,000,000	50,000,000,000
Blue whiting(=Poutassou)	*Micromesistius poutassou*	2	332	80	300	1,100,000,000	4,200,000,000
Japanese anchovy	*Engraulis japonicus*	4.2	698	20	22	32,000,000,000	35,000,000,000
Atlantic menhaden	*Brevoortia tyrannus*	1.6	266	162	400	660,000,000	1,600,000,000
**Total for above**		**53**	**8,725**			**260,000,000,000**	**600,000,000,000**
**Average of above[5]**				***15***	***33***		
Others[6]		47	7,894				
Others excluding krill[7,8]			7,746	*15*	*33*	230,000,000,000	530,000,000,000
**Total for reduction[9]**		**53**	**16,619**			**490,000,000,000**	**1,100,000,000,000**

1 Source: Cashion *et al.* (2017).

2 Calculated as the percentage shown for the species of the total capture used for reduction (16,619 thousand tonnes). For 'Others excluding krill', this is calculated as the tonnage for 'Others', less an estimated 147.82 thousand tonnes of krill used for reduction (see note 7).

3 Estimated mean weight (EMW/GEMW) range obtained in the present study for 2000-2019 (see text) for the species shown. An EMW is an estimated mean weight based on data for the same species, whereas a GEMW is an estimated mean weight extrapolated from data for other species.

4 The estimated number range is calculated from the capture tonnage destined for reduction and the estimated mean weight range (EMW/GEMW) for the species.

5 A GEMW (in italics) is calculated for the named species (i.e. the species named here). This is back-calculated from their total capture tonnage, and total estimated number range, destined for reduction. This GEMW is used to estimate fish numbers of unnamed species in 'Others excluding krill'.

6 According to Cashion *et al.* (2017), of the total fisheries capture used for reduction in 2010 (16,619 thousand tonnes), 47% was of 'other taxa'. This is assumed to include an estimated (see note 7) tonnage of Antarctic krill *(Euphausia superba),* since this crustacean is also partly used for reduction to meal and oil (Katevas 2014).

7 In 2010, 212 thousand tonnes of Antarctic krill were caught (FAO 2021a). On average, 70,750 tons (equating to 64,183 metric tonnes) of krill are used each year for fresh and frozen meats (Katevas 2014). Krill capture destined for reduction is here estimated to comprise the difference between these two figures, equating to 147.82 thousand tonnes.

8 It is assumed that fishes of unnamed species within 'Others excluding krill' have a similar overall mean weight as the fishes of named species. Their numbers are calculated from the GEMW for named species (see note 5). It is assumed that the tonnage shown for 'Others excluding krill' is entirely comprised of finfishes.

9 Source of total capture tonnage for reduction: Supplementary Table S2 in Cashion *et al.* (2017). This tonnage is based on 'reconstructed' marine catch, which includes capture missing from FAO capture statistics, though not discards. It therefore excludes any bycatch from reduction fisheries that was not so used, either discarded overboard or landed. It also excludes any freshwater species, such as silver cyprinid *(Rastrineobola argentea)* which is also used for fishmeal (Kubiriza *et al.* 2021). It excludes fishes used for reduction in the form of trimmings i.e. by-products from use as food (FAO 2020) and fishes used as direct feed or bait (FAO 2020). Capture for reduction, according to the FAO (2021c), totalled 14,985 thousand tonnes in 2010 and averaged 15,782 thousand tonnes annually for 2011-2019.

This table shows estimated numbers of wild-caught finfishes used for reduction to fish meal and oil (FMFO) in 2010. Numbers are calculated from fish tonnages destined for FMFO, calculated using data from Cashion *et al.* (2017), and estimated mean weights (EMWs/GEMWs) for wild-caught fishes obtained in the present study. Estimated numbers for 'Others excluding krill' are extrapolated from those for named species, based on the assumptions given in notes 6 and 8 above. For fishes excluded from the estimate, see note 9 above. The total estimated number of finfishes used for reduction in 2010 equals 490-1,100 billion, or 4.9×10^{11} - 1.1×10^{12}, individuals.
