# Supplementary material for: Estimating global numbers of fishes caught from the wild annually from 2000 to 2019
Source: Anim Welf. 2024 Feb 8;33:e6. doi: 10.1017/awf.2024.7 (PMC10951671; doi:10.1017/awf.2024.7)
Supplement: Mood and Brooke supplementary material 7 — Mood and Brooke supplementary material [file S0962728624000071sup007.pdf]

Estimated wild-caught finfish numbers destined for reduction to fishmeal and oil (2001-6)

Species[1]	Scientific name[1]	Country[1]	Average annual capture production (landings) 2001-2006 '000 tonnes[1]	Average annual capture production (landings) 2001-2006 '000 tonnes (FAO 2021a)[2]	Percent of total capture destined for reduction %[1]	Capture destined for reduction ('000 tonnes)[3]	Lower estimated mean weight (EMW/ GEMW)[4] (g). GEMWs in italics	Upper estimated mean weight (EMW/ GEMW)[4] (g). GEMWs in italics	Lower estimated numbers (2 significant figures)[5]	Upper estimated numbers (2 significant figures)[5]
Sandeels(=Sandlances) nei	*Ammodytes spp*	Denmark	388	388	100	388	10	10	39,000,000,000	39,000,000,000
Sandeels(=Sandlances) nei	*Ammodytes spp*	Faroe Islands	7	5	100	5	10	10	450,000,000	450,000,000
Sandeels(=Sandlances) nei	*Ammodytes spp*	Norway	92	77	100	77	10	10	7,700,000,000	7,700,000,000
Gulf menhaden	*Brevoortia patronus*	United States of America	479	479	100	479	95	127	3,800,000,000	5,000,000,000
Atlantic menhaden	*Brevoortia tyrannus*	United States of America	212	211	100	211	162	400	530,000,000	1,300,000,000
Norway pout	*Trisopterus esmarkii*	Norway	15	15	100	15	16	28	520,000,000	900,000,000
Norway pout	*Trisopterus esmarkii*	Denmark	36	36	100	36	16	28	1,300,000,000	2,200,000,000
Norway pout	*Trisopterus esmarkii*	Faroe Islands	1	1	100	1	16	28	42,000,000	72,000,000
Anchoveta(=Peruvian anchovy)	*Engraulis ringens*	Peru	7,200	7,202	98	7,058	10	29	250,000,000,000	710,000,000,000
Anchoveta(=Peruvian anchovy)	*Engraulis ringens*	Chile	1,268	1,268	98	1,242	10	29	43,000,000,000	120,000,000,000
Japanese anchovy	*Engraulis japonicus*	China	1,142	965	67	647	20	22	29,000,000,000	32,000,000,000
Japanese anchovy	*Engraulis japonicus*	Japan	425	427	50	214	20	22	9,700,000,000	11,000,000,000
European anchovy	*Engraulis encrasicolus*	South Africa	228	-	50	-	8	38	-	-
European anchovy	*Engraulis encrasicolus*	Morocco	19	18	50	9	8	38	240,000,000	1,100,000,000
Anchovies, etc. nei	*Engraulidae*	Thailand	155	155	50	78	*10*	*25*	3,200,000,000	7,900,000,000
Sardinellas nei	*Sardinella spp*	Thailand	128	126	50	63	*47*	*75*	840,000,000	1,300,000,000
Capelin	*Mallotus villosus*	Norway	229	229	50	114	17	50	2,300,000,000	6,700,000,000
Capelin	*Mallotus villosus*	Iceland	665	665	75	499	17	50	10,000,000,000	29,000,000,000
Capelin	*Mallotus villosus*	Faroe Islands	37	36	100	36	17	50	730,000,000	2,100,000,000
Capelin	*Mallotus villosus*	Canada	28	28	0	-	17	50	-	-
Blue whiting(=Poutassou)	*Micromesistius poutassou*	Norway	720	720	100	720	80	300	2,400,000,000	9,000,000,000
Blue whiting(=Poutassou)	*Micromesistius poutassou*	Iceland	359	359	95	341	80	300	1,100,000,000	4,300,000,000
Blue whiting(=Poutassou)	*Micromesistius poutassou*	Denmark	65	65	100	65	80	300	220,000,000	810,000,000
Blue whiting(=Poutassou)	*Micromesistius poutassou*	Faroe Islands	255	282	100	282	80	300	940,000,000	3,500,000,000
European sprat	*Sprattus sprattus*	Norway	5	6	100	6	9	9	650,000,000	650,000,000
European sprat	*Sprattus sprattus*	Denmark	258	257	100	257	9	9	30,000,000,000	30,000,000,000
Atlantic herring	*Clupea harengus*	Iceland	238	238	50	119	100	600	200,000,000	1,200,000,000
**Total for above**			**14,652**	**14,260**		**12,963**			**430,000,000,000**	**1,000,000,000,000**
**Mean individual weight of fishes destined for reduction[6]**							**13**	**30**		

Notes

1. Source: Wijkström (2012). Wijkström (2012) gives percentages of landings converted to fishmeal and fish oil, for species so used, for 14 countries with the largest fishmeal production in 2001-2006. Wijkström (2012) also gives average annual capture production (landings) of these species by these countries in 2001-2006, obtained from Péron et al. (2010) who obtained them from FAO databases. Of the species shown, Atlantic herring is a prime food fish (Wijkström 2012). According to Wijkström (2012), other high quality food fishes are also sometimes used for reduction (not shown or included) but percentages so used are not given by this author.

2. Average total annual capture production (landings) for the species and country shown, averaged for 2001-2006, as obtained from FishStatJ (FAO 2021a). In some cases, tonnages given by FishStatJ (FAO 2021a) differ from those given by Wijkström (2012), who obtained them from Péron et al. (2010). These differences are presumed to be due to rounding and FAO revisions. Note that if Wijkström's figures for total capture production were used, instead of FishStatJ capture production tonnages (FAO 2021a), a similar overall result of 440-1,000 billion fishes would have been obtained.

3. Capture destined for reduction is calculated as the percentage destined for reduction times the average annual capture production (2001-2006), obtained from FishStatJ (FAO 2021a).

4. Estimated mean weight (EMW/GEMW) range obtained in the present study for 2000-2019 (see text) for the species shown. An EMW is an estimated mean weight based on data for the same species, whereas a GEMW is an estimated mean weight extrapolated from data for other species.

5. The estimated number range is calculated from the capture tonnage destined for reduction and the estimated mean weight range (EMW/GEMW) for the species.

6. The mean individual fish weight is back-calculated from the total tonnage, and total estimated fish numbers, used for reduction.

This table shows estimated numbers of wild-caught finfishes caught for reduction to fishmeal and fish oil (FMFO), on average annually for 2001-2006. Capture tonnage destined for reduction is calculated from total capture production reported by the FAO (2021a) and percentages of total capture used for reduction, obtained from Wijkström (2012), which exclude some prime food fishes so used. Numbers are calculated from fish tonnages destined for FMFO and estimated mean weights (EMWs/GEMWs) for wild-caught fish species obtained in the present study.

Excluding some prime food fishes that were also used for reduction, a total of 13 million tonnes of finfish capture were used for FMFO annually in 2001-2006, comprising an estimated 430-1,000 billion, or 4.3×10^{11} - 1.0×10^{12}, individuals.
