# Supplementary material for: Estimating global numbers of fishes caught from the wild annually from 2000 to 2019
Source: Anim Welf. 2024 Feb 8;33:e6. doi: 10.1017/awf.2024.7 (PMC10951671; doi:10.1017/awf.2024.7)
Supplement: Mood and Brooke supplementary material 8 — Mood and Brooke supplementary material [file S0962728624000071sup008.pdf]

Estimated finfish numbers in wild capture certified by Friend of the Sea (FOS) (2014)

Species certified[1]	Species estimate based on, if different[2]	Scientific name	FOS certified capture (2014)[1] in tonnes	FAO capture production (landings) (2014)[3] in tonnes	Lower of FOS certified and FAO capture[4,5] in tonnes	Lower estimated mean weight (EMW/GEMW) (g) (GEMW in italics)[6]	Upper estimated mean weight (EMW/GEMW) (g) (GEMW in italics)[6]	Lower estimated numbers (2 significant figures)[7]	Upper estimated numbers (2 significant figures)[7]	Estimated numbers midpoint (2 significant figures)
Anchoveta(=Peruvian anchovy)[4b]		*Engraulis ringens*	6,100,000	3,140,029	3,140,029	10	29	110,000,000,000	310,000,000,000	210,000,000,000
European anchovy		*Engraulis encrasicolus*	337,300	282,066	282,066	8	38	7,400,000,000	34,000,000,000	21,000,000,000
Chilean jack mackerel		*Trachurus murphyi*	509,000	414,454	414,454	200	1,000	410,000,000	2,100,000,000	1,200,000,000
Gulf menhaden		*Brevoortia patronus*	87,000	385,022	87,000	95	127	690,000,000	920,000,000	800,000,000
Atlantic menhaden		*Brevoortia tyrannus*	174,000	174,032	174,000	162	400	440,000,000	1,100,000,000	750,000,000
Skipjack tuna		*Katsuwonus pelamis*	556,273	2,981,600	556,273	1,800	9,070	61,000,000	310,000,000	190,000,000
Argentine anchovy		*Engraulis anchoita*	3,000	13,955	3,000	15	26	120,000,000	200,000,000	160,000,000
Sockeye(=Red) salmon		*Oncorhynchus nerka*	135,000	191,154	135,000	2,300	3,600	38,000,000	59,000,000	48,000,000
Yellowfin tuna		*Thunnus albacares*	495,448	1,366,154	495,448	9,000	33,430	15,000,000	55,000,000	35,000,000
Bigeye tuna		*Thunnus obesus*	51,833	404,701	51,833	1,100	35,980	1,400,000	47,000,000	24,000,000
Atlantic cod		*Gadus morhua*	19,500	1,374,417	19,500	800	4,000	4,900,000	24,000,000	15,000,000
Haddock		*Melanogrammus aeglefinus*	15,000	288,747	15,000	900	1,800	8,300,000	17,000,000	13,000,000
Coho(=Silver) salmon		*Oncorhynchus kisutch*	126,000	37,368	37,368	5,400	5,400	6,900,000	6,900,000	6,900,000
Pink(=Humpback) salmon		*Oncorhynchus gorbuscha*	9,000	298,363	9,000	1,364	2,273	4,000,000	6,600,000	5,300,000
Chinook(=Spring=King) salmon		*Oncorhynchus tshawytscha*	126,000	12,755	12,755	1,814	8,341	1,500,000	7,000,000	4,300,000
Sole	Common sole	*Solea solea*	2,000	36,335	2,000	300	3,500	570,000	6,700,000	3,600,000
Gilthead seabream		*Sparus aurata*	3,000	6,721	3,000	1,009	1,009	3,000,000	3,000,000	3,000,000
Chum(=Keta=Dog) salmon		*Oncorhynchus keta*	9,000	337,587	9,000	3,182	8,182	1,100,000	2,800,000	2,000,000
European perch		*Perca fluviatilis*	1,750	33,097	1,750	1,200	1,200	1,500,000	1,500,000	1,500,000
Albacore		*Thunnus alalunga*	8,833	236,630	8,833	4,536	21,364	410,000	1,900,000	1,200,000
Pike-perch		*Sander lucioperca*	1,750	20,785	1,750	1,540	2,314	760,000	1,100,000	950,000
Blue cod	New Zealand blue cod	*Parapercis colias*	1,500	2,134	1,500	*1,820*	*1,877*	800,000	820,000	810,000
Swordfish		*Xiphias gladius*	4,350	120,355	4,350	13,738	60,000	73,000	320,000	190,000
Total for all species except Anchoveta			**2,676,537**	**9,018,432**	**2,324,880**			**9,200,000,000**	**39,000,000,000**	**24,000,000,000**
**Total**			**8,776,537**	**12,158,461**	**5,464,909**			**120,000,000,000**	**350,000,000,000**	**240,000,000,000**

Notes

1 Source: Potts *et al.* (2016).

2 With the exception of 'blue cod' and 'sole', the name of the species certified, according to Potts *et al.* (2016), exists as a category of species in capture production reported by the FAO (2021a). The scientific name and the estimated mean weight range are obtained for this FAO species category. Based on information obtained from the Friend of the Sea website (FOS 2023), 'blue cod' is assumed to mean New Zealand blue cod (*Parapercis colias*) and 'sole' is assumed to comprise common sole (*Solea solea*) and lemon sole (*Microstomus kitt*) for which EMWs are respectively 300-3,500g and 450-650g.

3 Total capture production (landings) tonnage, as reported by the FAO (2021a).

4 This estimate is based on FOS certified tonnages for 2014, obtained from Potts *et al.* (2016), and assumes these are generally correct. However, comparison of these tonnages with capture production reported by the FAO (2021a) suggests some may be anomalous, since they exceed the corresponding 2014 FAO tonnage. If the FAO tonnage for a species is correct and represents the whole catch, then the certified tonnage should not exceed it. This is adjusted for, as far as possible, by setting the certified tonnage to the lower of the reported certified tonnage, according to Potts *et al.* (2016), and the total 2014 tonnage reported by the FAO. The effect of these adjustments, for species other than anchoveta, is discussed in note 4a. Anchoveta is discussed in note 4b.

4a Comparison of FOS certified tonnages, reported by Potts *et al.* (2016), with FAO tonnages resulted in a downward adjustment of 13% from 2,677 to 2,325 thousand tonnes for species other than anchoveta. If the FOS certified tonnages reported by Potts *et al.* (2016) were used without any adjustment, for species other than anchoveta, then estimated numbers for non-anchoveta species certified by FOS would increase from a range of 9.2-39 (midpoint 24) billion to 11-47 (midpoint 29) billion.

4b Comparison of certified tonnage, reported by Potts *et al.* (2016), with FAO tonnage resulted in a downward adjustment of 49%, from 6,100 to 3,140 thousand tonnes for anchoveta. In the case of Anchoveta (also called Peruvian anchovy), the certified tonnage should nearly equal that reported by the FAO since virtually all capture was certified by FOS, according to Potts *et al.* (2016). Anchoveta landings each year are highly variable (FAO 2021a).

5 It is assumed there is no significant double certification and that fish numbers certified by FOS are in addition to those certified by MSC. Potts *et al.* (2016) believed multiple certification rates were negligible.

6 Estimated mean weights, i.e. EMWs or GEMWs, are as obtained in the present study for 2000-2019. EMWs are estimated mean weights based on fish weight data for the species. GEMWs are estimated mean weights extrapolated from data for other species.

7 Estimated numbers are calculated from the tonnage (i.e. the lower of reported certified tonnage and FAO tonnage), divided by the estimated mean weight (EMW/GEMW) range.

This table shows estimated finfish numbers in wild capture production tonnages certified by FOS, obtained from Potts *et al.* (2016). Based on certain assumptions (2, 4 & 5 above), this estimate shows the very large numbers that could potentially benefit from welfare standards within this scheme e.g. a requirement for humane slaughter methods. Estimated numbers are calculated from certified tonnages using estimated mean weights (EMWs or GEMWs) obtained in the present study. Estimated numbers total 120-350 (midpoint 240) billion, or 1.2-3.5 x 10^{11}, individuals including 110-310 (midpoint 210) billion anchoveta and 9.2-39 (midpoint 24) billion others.
