# Supplementary material for: Estimating global numbers of fishes caught from the wild annually from 2000 to 2019
Source: Anim Welf. 2024 Feb 8;33:e6. doi: 10.1017/awf.2024.7 (PMC10951671; doi:10.1017/awf.2024.7)
Supplement: Mood and Brooke supplementary material 9 — Mood and Brooke supplementary material [file S0962728624000071sup009.pdf]

Estimated finfish numbers in wild capture certified by Marine Stewardship Council (MSC) (2015)

Species certified[1]	Species estimate based on, if different[2]	Scientific name	MSC certified capture (2015)[1] in tonnes	FAO capture production (landings) (2015)[3] in tonnes	Lower of MSC certified and FAO capture[4,5] in tonnes	Lower estimated mean weight (EMW/GEMW) (g) (GEMW in italics)[6]	Upper estimated mean weight (EMW/GEMW) (g) (GEMW in italics)[6]	Lower estimated numbers (2 significant figures)[7]	Upper estimated numbers (2 significant figures)[7]	Estimated numbers midpoint (2 significant figures)
Alaska pollock(=Walleye poll.)		*Gadus chalcogrammus*	2,153,477	3,372,741	2,153,477	227	1,000	2,200,000,000	9,500,000,000	5,800,000,000
Herring	Atlantic herring	*Clupea harengus*	751,621	1,512,003	751,621	100	600	1,300,000,000	7,500,000,000	4,400,000,000
European pilchard(=Sardine)		*Sardina pilchardus*	22,313	1,176,338	22,313	20	20	1,100,000,000	1,100,000,000	1,100,000,000
Mackerel	Atlantic mackerel	*Scomber scombrus*	379,669	1,247,278	379,669	370	454	840,000,000	1,000,000,000	930,000,000
Atlantic cod		*Gadus morhua*	1,004,258	1,303,711	1,004,258	800	4,000	250,000,000	1,300,000,000	750,000,000
South American pilchard[4a]		*Sardinops sagax*	83,000	1,212	83,000	*90*	*159*	520,000,000	920,000,000	720,000,000
European anchovy		*Engraulis encrasicolus*	7,000	443,678	7,000	8	38	180,000,000	850,000,000	520,000,000
North Pacific hake		*Merluccius productus*	282,223	200,214	200,214	454	1,035	190,000,000	440,000,000	320,000,000
Smelts nei		*Osmerus spp, Hypomesus spp*	13,693	10,576	10,576	*20*	*107*	98,000,000	530,000,000	310,000,000
Yellowfin sole		*Limanda aspera*	164,944	123,067	123,067	300	700	180,000,000	410,000,000	290,000,000
Cape hakes		*Merluccius capensis, M.paradoxus*	129,810	294,182	129,810	*383*	*695*	190,000,000	340,000,000	260,000,000
Haddock		*Melanogrammus aeglefinus*	532,606	308,283	308,283	900	1,800	170,000,000	340,000,000	260,000,000
Skipjack tuna		*Katsuwonus pelamis*	688,993	2,810,493	688,993	1,800	9,070	76,000,000	380,000,000	230,000,000
Pacific cod		*Gadus macrocephalus*	229,912	458,693	229,912	1,200	4,545	51,000,000	190,000,000	120,000,000
Saithe(=Pollock)		*Pollachius virens*	267,078	293,643	267,078	2,200	4,500	59,000,000	120,000,000	90,000,000
Argentine anchovy		*Engraulis anchoita*	1,719	14,411	1,719	15	26	66,000,000	110,000,000	90,000,000
Blue grenadier		*Macruronus novaezelandiae*	121,748	177,788	121,748	1,500	1,500	81,000,000	81,000,000	81,000,000
Southern blue whiting		*Micromesistius australis*	38,107	58,389	38,107	400	600	64,000,000	95,000,000	79,000,000
Patagonian grenadier		*Macruronus magellanicus*	110,267	95,884	95,884	1,500	1,500	64,000,000	64,000,000	64,000,000
Golden redfish		*Sebastes marinus*	44,400	55,010	44,400	600	2,000	22,000,000	74,000,000	48,000,000
Pink(=Humpback) salmon		*Oncorhynchus gorbuscha*	81,645	444,829	81,645	1,364	2,273	36,000,000	60,000,000	48,000,000
European plaice		*Pleuronectes platessa*	29,887	107,550	29,887	598	1,100	27,000,000	50,000,000	39,000,000
Sockeye(=Red) salmon		*Oncorhynchus nerka*	92,339	189,065	92,339	2,300	3,600	26,000,000	40,000,000	33,000,000
Arrowtooth flounder		*Atheresthes stomias*	53,818	38,248	38,248	1,093	1,268	30,000,000	35,000,000	33,000,000
Vendace		*Coregonus albula*	1,746	6,648	1,746	40	120	15,000,000	44,000,000	29,000,000
Chum(=Keta=Dog) salmon		*Oncorhynchus keta*	81,652	351,959	81,652	3,182	8,182	10,000,000	26,000,000	18,000,000
Yellowtail flounder		*Limanda ferruginea*	17,000	7,319	7,319	500	600	12,000,000	15,000,000	13,000,000
Soles nei		*Soleidae*	61,759	7,271	7,271	*300*	*3,500*	2,100,000	24,000,000	13,000,000
Dover sole		*Microstomus pacificus*	11,156	6,179	6,179	*450*	*650*	9,500,000	14,000,000	12,000,000
Flathead sole		*Hippoglossoides elassodon*	20,083	11,921	11,921	*909*	*1,400*	8,500,000	13,000,000	11,000,000
European hake		*Merluccius merluccius*	3,215	135,483	3,215	174	1,400	2,300,000	18,000,000	10,000,000
Coho(=Silver) salmon		*Oncorhynchus kisutch*	73,632	26,855	26,855	5,400	5,400	5,000,000	5,000,000	5,000,000
Southern hake		*Merluccius australis*	12,544	27,748	12,544	2,000	9,000	1,400,000	6,300,000	3,800,000
Sablefish		*Anoplopoma fimbria*	11,060	18,655	11,060	2,268	4,536	2,400,000	4,900,000	3,700,000
Chinook(=Spring=King) salmon		*Oncorhynchus tshawytscha*	73,492	10,625	10,625	1,814	8,341	1,300,000	5,900,000	3,600,000
Shortspine thornyhead		*Sebastolobus alascanus*	1,190	1,273	1,190	*326*	*391*	3,000,000	3,600,000	3,300,000
Albacore		*Thunnus alalunga*	23,247	232,414	23,247	4,536	21,364	1,100,000	5,100,000	3,100,000
Lumpfish(=Lumpsucker)		*Cyclopterus lumpus*	3,860	13,120	3,860	836	2,892	1,300,000	4,600,000	3,000,000
Ling		*Molva molva*	17,946	44,375	17,946	6,800	6,800	2,600,000	2,600,000	2,600,000
Rock sole		*Lepidopsetta bilineata*	1,986	46,947	1,986	907	907	2,200,000	2,200,000	2,200,000
Petrale sole		*Eopsetta jordani*	1,679	2,644	1,679	667	1,175	1,400,000	2,500,000	2,000,000
Sole	Common sole	*Solea solea*	1,016	33,462	1,016	300	3,500	290,000	3,400,000	1,800,000

Picked dogfish		*Squalus acanthias*	2,239	15,725	2,239	835	5,781	390,000	2,700,000	1,500,000
Patagonian toothfish		*Dissostichus eleginoides*	11,016	22,938	11,016	6,000	10,000	1,100,000	1,800,000	1,500,000
Mackerel icefish		*Champsocephalus gunnari*	2,602	477	477	*305*	*611*	780,000	1,600,000	1,200,000
Halibut	Pacific halibut	*Hippoglossus stenolepis*	15,867	18,938	15,867	13,640	18,180	870,000	1,200,000	1,000,000
Yellowfin tuna		*Thunnus albacares*	10,896	1,385,976	10,896	9,000	33,430	330,000	1,200,000	770,000
English sole		*Pleuronectes vetulus*	340	319	319	*598*	*1,100*	290,000	530,000	410,000
Atlantic halibut		*Hippoglossus hippoglossus*	1,760	7,323	1,760	2,300	56,000	31,000	770,000	400,000
Widow rockfish		*Sebastes entomelas*	143	847	143	300	1,814	79,000	480,000	280,000
Swordfish		*Xiphias gladius*	4,885	120,065	4,885	13,738	60,000	81,000	360,000	220,000
Flounder	Kamchatka flounder	*Atheresthes evermanni*	220	293	220	1,093	1,268	170,000	200,000	190,000
Sea bass	European seabass	*Dicentrarchus labrax*	168	6,437	168	1,200	1,200	140,000	140,000	140,000
Longnosed skate		*Raja oxyrinchus*	715	419	419	*2,512*	*5,225*	80,000	170,000	120,000
Pike-perch		*Sander lucioperca*	180	18,809	180	1,540	2,314	78,000	120,000	97,000
Antarctic toothfish		*Dissostichus mawsoni*	2,153	4,846	2,153	28,000	28,000	77,000	77,000	77,000
Splitnose rockfish		*Sebastes diploproa*	55	15	15	*331*	*403*	37,000	45,000	41,000
Northern pike		*Esox lucius*	22	27,464	22	1,000	5,000	4,400	22,000	13,000
Chilipepper rockfish		*Sebastes goodei*	1	175	1	720	1,200	830	1,400	1,100
Alaska plaice		*Pleuronectes quadrituberculat.*	21,571	-	-	*598*	*1,100*	-	-	-
Rex sole		*Glyptocephalus zachirus*	2,874	-	-	*300*	*1,000*	-	-	-
Spotted spiny dogfish		*Squalus suckleyi*	799	-	-	*835*	*5,781*	-	-	-
Longspine thornyhead		*Sebastolobus altivelis*	1,050	-	-	*339*	*416*	-	-	-
**Total**			**7,782,346**	**17,353,249**	**7,185,339**			**7,800,000,000**	**26,000,000,000**	**17,000,000,000**

Notes

1 Source: Potts *et al.* (2016).

2 In most cases, the name of the species certified, according to Potts *et al.* (2016), also exists as a category of species in capture production tonnages reported by the FAO (2021a). The scientific name and the estimated mean weight range are obtained for this FAO species category. 'Herring', 'mackerel', 'halibut', 'sole', 'flounder' and 'sea bass' (for which there are no identically named FAO species categories) are assumed to comprise Atlantic herring, Atlantic mackerel, Pacific halibut, common sole, Kamchatka flounder and European seabass respectively (all of which are reported separately by the FAO), based on data on certified fisheries obtained from the MSC website (MSC 2023). Based on MSC (2023), 'rex sole' is assumed to mean *Glyptocephalus zachirus* and 'spotted spiny dogfish' is assumed to mean *Squalus suckleyi*, which are not reported separately in FAO capture production statistics.

3 Total capture production (landings) tonnage, as reported by the FAO (2021a).

4 This estimate is based on MSC certified tonnages for 2015, obtained from Potts *et al.* (2016), and assumes these are generally correct. However, comparison of these tonnages with capture production reported by the FAO (2021a) suggests some may be anomalous, since they exceed the corresponding FAO 2015 tonnage. If the FAO tonnage for a species is correct and represents the whole catch, then the certified tonnage should not exceed it. This is adjusted for, as far as possible, by setting the certified tonnage to the lower of the reported certified tonnage, according to Potts *et al.* (2016), and the 2015 tonnage reported by the FAO. There is one exception for this, where the certified tonnage was not adjusted despite exceeding the corresponding FAO tonnage, which is discussed in note 4a. The effect of these adjustments is discussed in 4b below. The effect of further adjustment, where the combined MSC and FOS certified tonnages exceed the FAO tonnage, is discussed in 4c.

4a The certified tonnage was not adjusted for South American Pilchard *(Sardinops sagax)*. It is assumed that certified tonnage for this species includes capture of other pilchards in the genus *Sardinops* (such as California pilchard *(Sardinops caeruleus)*), which are reported separately by the FAO but which FishBase (Froese & Pauly 2023) considers to be synonyms of *Sardinops sagax,* and which together had a reported capture production tonnage of 641 thousand tonnes in 2015.

4b Comparison of MSC certified tonnages, reported by Potts *et al.* (2016), with FAO tonnages resulted in a total downward adjustment of 8% from 7,782 to 7,185 thousand tonnes. If these reported certified tonnages were used without any adjustment - then the estimated number range for MSC would increase from 7.8-26 (midpoint 17) billion to 8.2-27 (midpoint 18) billion.

4c If the MSC certified tonnage had been further adjusted such that the combined FOS 2014 and MSC 2015 certified tonnage did not exceed the FAO 2015 tonnage, this would not have changed the estimate when rounded to the nearest billion.

5 It is assumed there is no significant double certification and that fish numbers certified by MSC are in addition to those certified by FOS. Potts *et al.* (2016) believed multiple certification rates were negligible.

6 Estimated mean weights, i.e. EMWs or GEMWs, are as obtained in the present study for 2000-2019. EMWs are estimated mean weights based on fish weight data for the species. GEMWs are estimated mean weights extrapolated from data for other species.

7 Estimated numbers are calculated from the tonnage (i.e. the lower of reported certified tonnage and FAO tonnage), divided by the estimated mean weight (EMW/GEMW) range.

This table shows estimated finfish numbers in wild capture production tonnages certified by MSC, obtained from Potts *et al.* (2016). Based on certain assumptions (notes 2, 4 & 5 above), this estimate shows the very large numbers that could potentially benefit from welfare standards within this scheme e.g. a requirement for humane slaughter methods. Estimated numbers are calculated from certified tonnages using estimated mean weights (EMWs or GEMWs) obtained in the present study. Estimated numbers total 7.8-26 (midpoint 17) billion, or 7.8×10^{9} - 2.6×10^{10}.
