# Supplementary material for: Estimating global numbers of fishes caught from the wild annually from 2000 to 2019
Source: Anim Welf. 2024 Feb 8;33:e6. doi: 10.1017/awf.2024.7 (PMC10951671; doi:10.1017/awf.2024.7)
Supplement: Mood and Brooke supplementary material 10 — Mood and Brooke supplementary material [file S0962728624000071sup010.pdf]

Table S10 Farmed finfish welfare law in the 30 top producing countries (by estimated numbers midpoint) for wild fish capture*

Rank in 2019	Country	Any law requiring welfare during slaughter of farmed fishes
1	Peru	Yes[1]
2	China	No[2]
3	Chile	Yes[3]
4	Indonesia	Yes[4,5]
5	India	Yes[6]
6	Denmark	Yes[7] [8]
7	Morocco	No[9]
8	Myanmar	No[10]
9	Vietnam	Yes[11]
10	Philippines	Yes[12]
11	Japan	No[13]
12	Thailand	No[14]
13	Russia	No[15]
14	Norway	Yes[16] [17]
15	Bangladesh	No[18]
16	Turkey	Yes[19]
17	Malaysia	Yes[20]
18	Iceland	Yes[21] [22]
19	South Africa	Yes[23]
20	South Korea	No[24]
21	USA	No[25]
22	Mexico	Yes[26]
23	Sweden	Yes[7] [27]
24	Cambodia	?[28]
25	Poland	Yes[7] [29]
26	Tanzania	No[30]
27	Ukraine	Yes[31]
28	Uganda	Yes[32]
29	Namibia	Yes[33]
30	Spain	Yes[7] [34]

* General notes.
This table indicates where national welfare law applies to fish slaughter, in the context of fish farming, in the top producing countries for wild-caught finfishes (Table 5 and Table S5 of the supplementary materials). This analysis is based on the wording of law and the authors' interpretation of it, and does not consider enforcement. Laws may protect fish specifically or as vertebrates, animals or living creatures. Most welfare laws for farmed fish slaughter comprise a requirement to avoid causing unnecessary suffering. Laws solely addressing malicious cruelty are not included in this analysis. Where necessary, texts were translated into English using Google Translate. All links were accessed on 27 October 2023.

---

[1] Animal Protection and Welfare Law 30407 (https://faolex.fao.org/docs/pdf/per157968.pdf (in Spanish)). Applies to vertebrates. Article 14 states that, for the purposes of the application of this law, any species of vertebrate animals kept in captivity are recognized as sentient beings. Article 5 concerns duty of care. Article 16 implies a general requirement for welfare of farm animals during rearing, transport and slaughter. The sacrifice must cause instant death or immediate animal unconsciousness.

[2] There is no nationwide animal protection law in China covering all animal species. Source: Animal Protection Index 2020 – People's Republic of China. World Society for the Protection of Animals https://api.worldanimalprotection.org/sites/default/files/api_2020_-_china_2.pdf

[3] Law 20380 on Animal Protection 2009 (https://faolex.fao.org/docs/pdf/chi90362.pdf (in Spanish)). Chile Government. Article 3 states that any person who has an animal, in whatever capacity, must take care of it. Article 11 states that, in the benefit and sacrifice of animals, rational methods should be used to avoid unnecessary suffering. It is assumed here that this law covers fish species since Article 13 indicates that the provisions of this law apply to aquatic animals and Article 19 refers to vertebrate animals.

[4] Husbandry and Animal Health Law Number 18 Year 2009 (https://faolex.fao.org/docs/pdf/ins98701.pdf), dated June 4, 2009. Indonesian Government. Applies to vertebrates and some invertebrates that can feel pain e.g. crabs. Article 66. Slaughtering and killing of animals shall be conducted properly so that the animals feel free from pain and fear.

[5] Regulation of the Government of the Republic of Indonesia Number 95 of 2012. Chapter III animal welfare. As with Law 18, vertebrates and also invertebrates that can feel pain are covered. The principle of freedom (definition based on the Five Freedoms) shall be applied to…maintenance and care, transportation… slaughtering and killing…. This includes slaughter by ways that do not cause fear and stress, and ending suffering as soon as possible. There are no fish-specific requirements http://www.flevin.com/id/lgso/translations/JICA%20Mirror/english/4936_PP_95_2012_e.html

[6] Cruelty to Animals Act 1960 (https://faolex.fao.org/docs/pdf/ind39976.pdf). Indian Government. Animal is defined as any living creature other than a human being. Treating animals cruelly includes subjecting them to unnecessary pain or suffering…Nothing in this section applies to killing an animal for food unless this was accompanied by the infliction of unnecessary pain or suffering.

[7] EU COUNCIL REGULATION (EC) No 1099/2009 of 24 September 2009 on the protection of animals at the time of killing. European Commission. Article 3.1, which gives a general requirement to spare animals avoidable pain, distress or suffering; covers fish species, but there are no detailed requirements for them https://eur-lex.europa.eu/legal-content/EN/ALL/?uri=CELEX:32009R1099

[8] EU COUNCIL REGULATION (EC) No 1099/2009 applies in Denmark (see note 7). Under the Danish Animal Welfare Act (133 of 2020) (https://faolex.fao.org/docs/pdf/den217597.pdf (in Danish)) Chapter 6, Section 25, whoever wants to kill an animal must ensure that the animal is killed as quickly and as painlessly as possible. This Act does not define 'animal', it is here interpreted as applying to all animals, since its stated purpose is to promote good animal welfare and respect for animals as living and sentient beings.

[9] Animal welfare law is extremely limited in Morocco. Source: Animal Protection Index 2020 – Kingdom of Morocco. World Society for the Protection of Animals https://api.worldanimalprotection.org/sites/default/files/morocco-animal-protection-index-report-2020.pdf

[10] The Animal Health and Development Law 1993 (https://faolex.fao.org/docs/pdf/mya36263.pdf). Myanmar Government. This Act provides some anti-cruelty protection for some animals. However, this Act was amended by Law No. 36 /2010 (https://faolex.fao.org/docs/pdf/mya213762.pdf) to define 'animals' as including only mammals, birds and honey bees.

[11] Law on Veterinary Medicine 2015 (https://faolex.fao.org/docs/pdf/vie168546.pdf). Vietnam Government. This law specifically includes fishes, crustaceans and other aquatic animals. Article 21 on treatment of animals states that organizations and individuals engaging in aquaculture have the responsibility to minimize pain and fear, and to treat them humanely in aquaculture, transportation, slaughter and destruction.

[12] The revised rules for implementing the Animal Welfare Act (AWA) of 1998 Republic Act 8485 as amended by RA 10631 (https://paws.org.ph/downloads/3.IRR of RA 8485 as amended by RA 10631.pdf). Philippines Government. 'Animal' is defined to include aquatic animals which are defined to include fishes and crustaceans. These rules shall be strictly construed in favour of protecting the Five Freedoms. Inhumane handling and improper stunning of animals for slaughter is defined as an act of cruelty.

[13] Act on Welfare and Management of Animals (Act No. 105 of October 1, 1973 https://www.env.go.jp/nature/dobutsu/aigo/1_law/files/aigo_kanri_1973_105_en.pdf). Article 40 of this Act states that, where an animal must be destroyed, a method that minimizes as much as possible the pain and distress to the animal shall be used. However, fishes are not included in the definition of 'protected animal' in Article 44. Government Standards relating to the Methods of Destruction of Animals (Notice of the Prime Minister's Office No. 40 of July 4, 1995 (https://www.env.go.jp/nature/dobutsu/aigo/2_data/laws/shobun_en.pdf)) state that the manager and destruction performer shall endeavour to use a method that does not cause pain or distress; the method of killing must minimise pain or distress as much as possible, or shall be any other normal method that is socially accepted. While it is stated that these shall be considered when killing any animal, the target species for these standards are the 'protected animals' listed in Article 44 of the Act, which are confined to mammals, birds and reptiles.

[14] The Cruelty Prevention and Welfare of Animals Act, B.E. 2557 (2014) (https://faolex.fao.org/docs/pdf/tha181025.pdf). Thailand Government. Under this law no person shall perform any act which is deemed an act of cruelty to animal without justification. Cruelty is an act, or failure to act, which causes an animal to suffer physically or mentally. The act covers animals used for food though it does not state the species covered and it is here assumed that fishes are included. Section 21 (1) states that killing an animal for food shall not be deemed cruelty The act appears to exclude slaughter from welfare provisions.

[15] The Russian Federal Law No. 498-FZ 'On responsible treatment of animals' of 2018 (https://faolex.fao.org/docs/pdf/rus183356.pdf) explicitly excludes farmed and wild animals from its scope. Article 245 of the Russian Penal Code https://www.imolin.org/doc/amlid/Russian_Federation_Criminal_Code.pdf) addresses cruelty to animals for malicious or mercenary motives. Besides this Article, there is no legislation protecting the welfare of farm animals during slaughter. Source: Animal Protection Index 2020 – Russian Federation. World Society for the Protection of Animals https://api.worldanimalprotection.org/sites/default/files/api_2020_-_russia.pdf

[16]Animal Welfare Act 2009. Norwegian Government. This law specifically includes fishes as well as decapods, squid and octopi. Farmed animals must be stunned prior to killing to ensure loss of consciousness, unless the killing method provides immediate unconsciousness https://www.regjeringen.no/en/dokumenter/animal-welfare-act/id571188/

[17] Decree No. 1250 of 2006 relative to slaughterhouses and processing facilities for aquaculture animals (https://faolex.fao.org/docs/pdf/nor67734.pdf (in Norwegian)). Norwegian Government. Fish must be anesthetized before or at the same time as killing and be anesthetized until death occurs. Anaesthesia should be done by a suitable method that does not cause the fish significant stress or pain.

[18] Animal Welfare Act, 2019 (Act No. 12 of 2019) (http://bdlaws.minlaw.gov.bd/act-1292.html?hl=1 (in Bengali)). This act excludes fishes (http://bdlaws.minlaw.gov.bd/act-1292/section-49029.html (in Bengali)). It does, however, permit any animals not specifically included in the act to be subsequently included by Government declaration

[19] Animal Protection Law no: 5199, 2004 (http://www.lawsturkey.com/law/5199-animal-protection-law). Turkish Government. This law covers animals slaughtered for food purposes. It includes vertebrates and invertebrates in the definition of wild animals, which is the only place where species are defined in Article 3 which explains the terms. This law states that all animals have a right to life within the provisions of the law. When slaughtered for food, this must be done in the least painful manner taking into account the special conditions required by religious rules.

[20] Animal Welfare Act 2015 (https://faolex.fao.org/docs/pdf/mal176896.pdf). Under Section 24, owners have a duty to take reasonable steps to fulfil the needs of animals, including protection from pain and suffering. Nothing in this section applies to the lawful destruction of any animal for any reasonable purposes in an appropriate and humane manner. The Act includes any living creature other than a human, including aquatic animals but excluding wildlife.

[21]Act on animal welfare 2013 No 55, 8 April (https://www.government.is/lisalib/getfile.aspx?itemid=d1718344-68cf-11e8-9429-005056bc4d74). Icelandic legislation. Article 2 Scope. This Act shall apply to vertebrates as well as to decapods, squid and honey bees…The Act shall not apply to regular fishing activities nor to the catching of wild fish. Article 21 Killing. Animals must be killed swiftly and painlessly, and where possible without other animals becoming aware of the killing…Animals in human custody must be stunned unconscious before being killed, except where the killing method used produces immediate loss of consciousness... The Minister shall issue a Regulation containing more detailed provisions regarding the persons authorised to kill animals, the killing methods to be used, and prohibited means of killing.

[22] Regulation No. 300 on aquatic animal welfare, disease prevention and health inspections of fishing farms (https://faolex.fao.org/docs/pdf/ice209893.pdf (in Icelandic). Article 12 Killing of fish. Farmed fish must be deprived of consciousness before bleeding and the unconsciousness must last at least from the beginning of bleeding until death. Equipment for the deprivation of consciousness must be used correctly and without causing unnecessary distress to farmed fish. Deprivation of consciousness shall be carried out by a blow to the head, drug anaesthesia or another approved method.

[23] The Animal Protection Act of 1962 does not explicitly state that fish are covered (https://www.gov.za/sites/default/files/gcis_document/201505/act-71-1962.pdf) but has been interpreted to include fishes as 'other… animal' by the NSPCA (Bilchitz DI and Wilson AP 2022 Key animal law in South Africa. In *Routledge Handbook of Animal Welfare* pp 426-435. Routledge. https://api.taylorfrancis.com/content/chapters/edit/download?identifierName=doi&identifierValue=10.4324/9781003182351-38&type=chapterpdf). The NSPCA has the statutory power to institute private prosecutions in this country (https://nspca.co.za/about-us). Under this Act, it is an offence to, by wantonly or unreasonably or negligently doing or omitting to do any act or causing or procuring the commission or omission of any act, cause any unnecessary suffering to any animal. No exception to this requirement is given, relating to slaughter for food or otherwise.

[24] Article 13 of the Protection of Animals Act (https://faolex.fao.org/docs/pdf/kor107509.pdf) states that no one shall slaughter an animal in a cruel or revolting manner, or cause unnecessary pain, fear, or stress during the slaughtering process. However, under Article 2, the Act applies only to mammals, birds and animals prescribed by Presidential Decree. According to Enforcement Decree of the Protection of Animals Act, given on the Korean Law Information Centre website (https://www.law.go.kr/LSW/eng/engLsSc.do?y=0&x=0&menuId=1&query=Animal+Protection+Decree#liBgcolor3), 'Animals prescribed by Presidential Decree' in Article 2 means reptiles, amphibia, and fish: provided that those kept for the purpose of human consumption shall be excluded.

[25] Currently, there are no federal or state laws protecting the welfare of farmed fish (source: Levenda K 2013 Legislation to protect the welfare of fish. *Animal L 20:* 119 https://www.animallaw.info/sites/default/files/lralvol20_1_119.pdf. The Humane Slaughter Act 1978 (https://uscode.house.gov/view.xhtml?path=/prelim@title7/chapter48&edition=prelim and https://uscode.house.gov/statviewer.htm?volume=92&page=1069) which is a federal law, does not cover fishes.

[26] Mexican Official STANDARD NOM-033-SAG/ZOO-2014, Methods for killing domestic and wild Animals (https://faolex.fao.org/docs/pdf/mex148241.pdf (in Spanish)) requires all farm animals to be stunned before slaughter. This federal standard covers vertebrates and invertebrates.

[27] EU COUNCIL REGULATION (EC) No 1099/2009 applies in Sweden (see note 7). The Animal Welfare Act (2018: 1192) (https://faolex.fao.org/docs/pdf/swe186517.pdf (in Swedish) states that animals must be spared unnecessary suffering and discomfort when slaughtered. An animal that is slaughtered or otherwise euthanized by bleeding must be stunned. Before the animal is dead, no other measures may be taken. The Animal Protection Ordinance (2019:66) (https://faolex.fao.org/docs/pdf/swe186518.pdf (in Swedish) states that when the slaughter or killing is carried out using a method that requires that the animal must first be stunned, the stunning must be administered so that the animal quickly becomes unconscious. Consciousness must not return. Neither the Act or the Ordinance define animal, which is therefore interpreted here to mean all animals. Also, the reference to fish in the Ordinance implies these are covered by the term 'animal'.

[28] The Law on Animal Health and Production (No. NS/RKM/0116/003), dated 2016, has animal welfare as one of its objectives. Source FAOLEX database http://www.fao.org/faolex/results/details/en/c/LEX-FAOC173984/. An English translation was not obtained.

[29] EU COUNCIL REGULATION (EC) No 1099/2009 applies in Poland (see note 7). The Animal Protection Act of 1997 (https://faolex.fao.org/docs/pdf/pol129621.pdf (in Polish)) states that every animal requires humane treatment (Article 5) and that a vertebrate animal in a slaughterhouse may be killed only after being rendered unconscious by persons with appropriate qualifications (Article 34).

[30] The Tanzanian ANIMAL WELFARE ACT of 2008 (https://faolex.fao.org/docs/pdf/tan85327.pdf) has welfare requirements for slaughtering animals (instantaneous killing or instantaneously rendering an animal unconscious) and the term 'animal' includes any vertebrate or invertebrate other than a human being. However, the Act does not apply to animals governed by the Fisheries Act (https://faolex.fao.org/docs/pdf/tan53024.pdf), which covers aquaculture and fishing, nor the Wildlife Conservation Act (see https://faolex.fao.org/docs/pdf/tan214436.pdf). Neither of these Acts appears to cover welfare.

[31] Under Article 17 (also Article 4) of the Ukrainian Law on the Protection of Animals from Cruelty (2006) (https://faolex.fao.org/docs/pdf/ukr176335.pdf (in Ukrainian)), translated by Google Translate, animals are protected during killing, including killing for economically useful products. Killing must be carried out by methods that avoid animal suffering, and it is forbidden to use inhumane methods of killing animals which lead to death by suffocation, or by electric shock, … overheating and other painful methods. Processing of animals is allowed only after they are killed. This law covers farmed and wild animals, though it does not specify the species covered. The law is here assumed to cover fishes since it does not state otherwise and Article 299 of the Criminal Code (https://www.wipo.int/wipolex/en/text/438635 (in Ukrainian)) on cruel treatment of animals covers vertebrates.

[32] Under Section 2 of the Animals (Prevention of Cruelty) Act (https://ulii.org/akn/ug/act/ord/1957/25/eng@2000-12-31), it is an offence to kill any animal in an unnecessarily cruel manner. Nothing in this section applies to the destruction of any animals as food for mankind, unless the destruction or the preparation was accompanied by the infliction of unnecessary suffering. Since the Act does not define 'animal' it is assumed here to include fishes.

[33] Under the ANIMALS PROTECTION ACT 71 OF 1962 (https://faolex.fao.org/docs/pdf/nam126907.pdf), it is an offence to, by wantonly or unreasonably or negligently doing or omitting to do any act or causing or procuring the commission or omission of any act, cause any unnecessary suffering to any animal. No exception to this requirement is given, relating to slaughter for food or otherwise. Although fishes are not listed in the definition of 'animal', it is else reported that the government considers the law applies to vertebrates (website of The Namibian daily newspaper on 26 September 2019 https://namibian.com.na/new-animal-welfare-bill-on-the-cards/) and it is here assumed that a fish would be covered as an 'other…animal'.

[34] EU COUNCIL REGULATION (EC) No 1099/2009 applies in Spain (see note 7), as stated in Law 37 of 2014 (https://faolex.fao.org/docs/pdf/spa130834.pdf (in Spanish)). Article 6 of Law 32/2007 on the Care of Animals, which covers vertebrate production animals, states that standards on construction, facilities, equipment and operation of slaughterhouses shall prevent unnecessary agitation, pain or suffering (https://faolex.fao.org/docs/pdf/spa76384.pdf (in Spanish). Law 7/2023 Protection of the Rights and Welfare of Animals does not cover production animals (https://faolex.fao.org/docs/pdf/spa216878.pdf (in Spanish)).
